# LC3B Mediated SETDB1‐Accounted Alcoholic Steatohepatitis via Lipidation‐Dependent LAP and Lipidation‐Independent Nuclear Stabilization

**DOI:** 10.1002/advs.202513189

**Published:** 2026-05-19

**Authors:** Yi Zhang, Tan Wei, Jiahang Wu, Chuixu Lin, Dongbo Zhu, Yanhui Li, Shuting Shi, Shishun Huang, Leiming Jiang, Hongzhi Wang, Meiqi Song, Pengfei Gao, Xu Wu, Mingjian Fan, Chaofeng Wei, Qian Wang, Lihui Qu, Zhigang Wang

**Affiliations:** ^1^ KingMed School of Laboratory Medicine Guangzhou Medical University Guangzhou China; ^2^ Engineering Technology Research Center of Intelligent Diagnosis For Infectious Diseases in Guangdong Province Guangzhou China; ^3^ Guangdong Provincial Engineering Research Center For Early Warning and Diagnosis of Respiratory Infectious Diseases Guangzhou China; ^4^ Guangzhou Key Laboratory For Clinical Rapid Diagnosis and Early Warning of Infectious Diseases Guangzhou China; ^5^ Guangzhou KingMed Diagnostics Group Co., Ltd Guangzhou China; ^6^ Department of Kinesiology and Nutrition University of Illinois Chicago Chicago Illinois USA; ^7^ Departments of Laboratory Diagnosis The Fifth Affiliated Hospital of Harbin Medical University Daqing China; ^8^ School of Basic Medical Sciences Guangzhou Medical University Guangzhou China

**Keywords:** alcohol‐associated liver disease, cGAS‐STING pathway, LC3‐associated phagocytosis, nuclear stability, SETDB1

## Abstract

Alcohol‐associated liver disease (ALD) progresses from steatosis to steatohepatitis, but the underlying mechanisms remain unclear. Here, we investigated SETDB1's role in ALD progression involving LC3B‐mediated LC3‐associated phagocytosis (LAP). SETDB1 expression was progressively downregulated in livers of alcohol‐fed mice and ethanol‐treated hepatocytes, correlating with disease severity. *Setdb1* HKO mice exhibited accelerated ALD progression, developing severe steatosis, inflammation, and fibrosis even under pair‐fed conditions, indicating SETDB1 deficiency enhances disease susceptibility to nutritional stressors. Mechanistically, SETDB1 acted as a transcriptional cofactor for ERG to promote *Map1lc3b* transcription. SETDB1 deficiency impaired LAP by disrupting Rubicon membrane localization, causing defective lipid droplet clearance and enhanced cGAS‐STING activation. The ATG16L1 WD40 domain was essential for this LAP‐mediated protection. LC3B restoration in *Setdb1* HKO mice ameliorated steatosis, inflammation, and liver injury. Notably, the lipidation‐deficient LC3B‐G120A mutant failed to rescue steatosis but partially suppressed inflammation, revealing a lipidation‐independent LC3B function. We demonstrate lipidated LC3B mediates cytoplasmic LAP‐dependent lipid clearance, while non‐lipidated LC3B translocates to the nucleus, reducing R‐loop accumulation, preserving genomic stability, and restraining cGAS‐STING‐driven inflammation. Collectively, these findings define a protective SETDB1‐ERG‐LC3B axis restraining ALD progression and reveal dual LC3B functions, offering mechanistic insight and a potential therapeutic strategy for intercepting steatosis‐to‐steatohepatitis transition.

## Graphical abstract

SETDB1 is progressively downregulated in ALD, correlating with disease severity. SETDB1 deficiency impairs LAP by disrupting Rubicon membrane localization, leading to defective lipid droplet clearance. Concurrently, loss of SETDB1 reduces nuclear LC3B, causing R‐loop accumulation and cGAS‐STING‐driven inflammation. Lipidated LC3B mediates LAP‐dependent lipid clearance, whereas non‐lipidated LC3B translocates to the nucleus to preserve genomic stability.

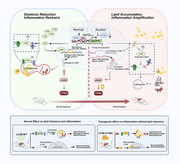

**Abbreviations**
AAV8: adeno‐associated virus serotype 8
AF: alcohol‐fed
ALD: alcohol‐associated liver disease
ALT: alanine aminotransferase
AST: aspartate aminotransferase
BAF1: bafilomycin A1
ChIP: chromatin immunoprecipitation
Co‐IP: co‐immunoprecipitation
DAMP: damage‐associated molecular pattern
DRIP‐qPCR: DNA‐RNA immunoprecipitation quantitative PCR
ELISA: enzyme‐linked immunosorbent assay
ERG: ETS‐related gene
FBS: fetal bovine serum
GO: gene Ontology
H&E: hematoxylin and eosin
H3K9me3: histone H3 lysine 9 trimethylation
HKO: hepatocyte‐specific knockout
HRP: horseradish peroxidase
IL: interleukin
ITS‐G: insulin‐transferrin‐selenium
LAP: LC3‐associated phagocytosis
LC3B: microtubule‐associated protein 1 light chain 3 beta
Map1lc3b: microtubule‐associated protein 1 light chain 3 beta (gene)
MOI: multiplicity of infection
MPO: myeloperoxidas
NAS: NAFLD activity score
NC: negative control
NES: nuclear export signal
NLS: nuclear localization signal
PE: phosphatidylethanolamine
PF: pair‐fed
SETDB1: SET domain bifurcated 1
TC: total cholesterol
TG: triglyceride
WD40: WD40 repeat domain

## 1 Introduction

Alcohol‐associated liver disease (ALD) is recognized as a significant public health concern globally, characterized by a spectrum of liver injuries resulting from excessive alcohol consumption [1, 2]. The pathophysiological progression of ALD typically encompasses several stages, including steatosis, inflammation, fibrosis, and ultimately cirrhosis [3, 4]. Hepatosteatosis is often one of the earliest manifestations of ALD and is marked by the accumulation of lipids within hepatocytes [2]. Oxidative stress [5], intestinal dysbiosis [6], disordered lipid metabolism [7], and toxic lipid deposition [8] are considered core triggers for the progression from alcoholic hepatosteatosis to steatohepatitis. Clinical research suggests that simple steatosis can manifest as a clinically stable state that does not advance to inflammation [9, 10]. However, it is the pathological stimuli that initiate this transformation, while the molecular mechanisms that uphold this phenotypic stability threshold remain to be elucidated.

SETDB1, also known as SET domain bifurcated 1, is a histone methyltransferase that specifically catalyzes histone H3 at lysine 9 trimethylation (H3K9me3) [11]. This post‐translational modification is crucial for the establishment of heterochromatin, leading to transcriptional repression of target genes [12]. Structurally, SETDB1 contains three critical functional domains that coordinate its epigenetic regulatory roles. The SET domain functions as a histone methyltransferase catalyzing H3K9me3 to mediate transcriptional repression and heterochromatin formation [13]. The Methyl‐CpG Binding domain enables sequence‐specific DNA binding, anchoring SETDB1 to methylated CpG regions to establish localized heterochromatin [14]. The Tudor domain mediates protein‐protein interactions by recognizing methylated lysine residues on partner proteins [15], facilitating recruitment of chromatin remodelers like HDAC complexes [16]. Our prior studies revealed that SETDB1 expression is downregulated in ALD and promotes hepatic steatosis development [17]. Nonetheless, the role of SETDB1 in facilitating the transition from alcoholic steatosis to steatohepatitis is still ambiguous. Furthermore, it remains to be determined if SETDB1 plays a part in additional transcriptional regulatory mechanisms beyond its epigenetic modification capabilities.

Microtubule‐associated protein 1 light chain 3 beta (*Map1lc3b*) plays dual roles in both canonical autophagy [18] and LC3‐associated phagocytosis (LAP) [19]. In canonical autophagy, cytosolic LC3B‐I is conjugated to phosphatidylethanolamine (PE) via ATG7 and ATG3, forming lipidated LC3B‐II that associates with autophagosomal membranes to mediate cargo recognition and degradation [20, 21, 22]. Beyond classical autophagy, LC3B is also recruited to single‐membrane phagosomes in LAP, a process triggered by pattern recognition receptors that requires Rubicon and NADPH oxidase‐generated ROS but is independent of the ULK1 complex [19, 23, 24, 25]. In the context of ALD, LAP in Kupffer cells serves as a critical immune‐metabolic checkpoint [19, 26, 27]. Upon engulfment of apoptotic hepatocytes, the recruitment of Rubicon and the lipidation of LC3B promote the rapid maturation of the phagosome into a degradative LAPosome [24, 25]. This process ensures the efficient clearance of apoptotic debris and, crucially, sequesters and degrades intracellular damage‐associated molecular patterns (DAMPs), such as mitochondrial and nuclear DNA [26, 27]. By preventing DNA leakage into the cytosol, functional LAP in Kupffer cells restrains the activation of the cGAS‐STING pathway, thereby limiting the production of type I interferons and pro‐inflammatory cytokines [24, 26, 28]. Additionally, metabolites derived from degraded apoptotic cells can promote the M2 anti‐inflammatory polarization of Kupffer cells, further resolving hepatic inflammation [26]. Impaired LAP function results in the accumulation of uncleared apoptotic debris, exacerbating hepatic inflammation and accelerating the transition from simple steatosis to steatohepatitis [26]. Thus, LC3B‐mediated LAP serves as a molecular checkpoint linking apoptotic cell clearance to innate immune homeostasis, and its dysfunction may represent a key determinant in the pathogenic switch from alcoholic fatty liver to progressive inflammatory liver disease. However, whether SETDB1 regulates the progression of alcoholic liver disease through modulating LAP remains largely unknown.

In this study, we aimed to elucidate the role of SETDB1 in the transition from alcoholic steatosis to steatohepatitis and to investigate whether this function is mediated by the LAP pathway. Using a combination of hepatocyte‐specific *Setdb1* knockout mice, cellular models of ethanol‐induced lipotoxicity, and AAV‐mediated gene delivery, we demonstrate that SETDB1 deficiency significantly exacerbates alcohol‐induced liver injury. Our findings reveal a previously unrecognized mechanism by which SETDB1, acting as a transcriptional cofactor, controls the expression of *Map1lc3b* and the subsequent functionality of LAP. This work identifies a critical SETDB1‐LC3B axis that links epigenetic regulation to innate immune homeostasis, offering new insights into the pathogenesis of ALD and highlighting potential therapeutic targets.

## 2 Materials and Methods

### 2.1 Cell Culture

The AML12 (RRID: CVCL_0140) and HEK293T (RRID: CVCL_0063) cell lines were obtained from Procell Life Science and Technology Co., Ltd. (Wuhan, China; Cat No. CL‐0602 and CL‐0005, respectively). All cell lines were authenticated by STR profiling and confirmed mycoplasma‐free. The AML12 cells were maintained in DMEM/F12 medium enriched with 10% fetal bovine serum and 1% ITS‐G (containing insulin, transferrin, and selenium; final concentrations: 5 µg/mL insulin, 5 µg/mL transferrin, and 5 ng/mL selenous acid). HEK293T cells were cultured in DMEM supplemented with 10% FBS and 1% penicillin–streptomycin. All cells were incubated at 37°C in a humidified atmosphere containing 5% CO_2_.

### 2.2 Construction of Knockdown and Overexpression *Setdb1* AML12 Cells

HEK293T cells were seeded in 10 cm dishes and cultured until ∼60% confluence. Cells were co‐transfected with either sh*Setdb1* (pLKO.1‐puro, see Table S1 for Sequence information of *Setdb1* shRNA) or oe*Setdb1* (pCDH‐CMV‐*Setdb*1) plasmids, along with packaging plasmids psPAX2 and pMD2.G using PEI reagent. At 72 h post‐transfection, viral supernatant was collected, filtered (0.45 µm), and concentrated by ultracentrifugation. For infection, AML12 hepatocytes were incubated with viral particles in the presence of 8 µg/mL polybrene for 1 h at 37°C, then reseeded into T25 flasks. Selection began 24 h later using 2 µg/mL puromycin (7‐day treatment). Stable pools were validated by qPCR (for *Setdb1* knockdown/overexpression) and western blot (for SETDB1 protein levels).

### 2.3 Animal

All animal experiments received approval from the Institutional Animal Care and Use Committee at Guangzhou Medical University (approval number: G2024‐852) and adhered to the National Institutes of Health's Guide for the Care and Use of Laboratory Animals, conducted in accordance with the ARRIVE 2.0 guidelines [29].

Eight‐week‐old male C57BL/6N mice were purchased from the Guangdong Medical Laboratory Animal Center, China. Lieber‐DeCarli ALD model was established as described previously [30]. Mice were fed with the Lieber‐DeCarli alcohol liquid diet (alcohol‐fed, AF) or isocaloric maltose dextrin control liquid diet (pair‐fed, PF) for 4 or 8 weeks following a one‐week acclimation period. During the acclimation week, AF mice were gradually introduced to increasing concentrations of ethanol until reaching the final concentration, while PF mice received a standard control diet. The amount of food given to the PF mice was the same as the AF mice consumed the previous day. Lieber‐DeCarli ALD model diets were purchased from Trophic Animal Feed High‐Tech Co., Ltd. (Nantong, China; alcohol‐fed diets TP4030B: 19% carbohydrates, 35% fat, 18% protein, 28% alcohol; pair‐fed diets TP4030C: 47% carbohydrates, 35% fat, 18% protein). A fresh diet was supplied once daily, and diet consumption was quantified throughout the experiment. Body weights were monitored every five days.

Hepatocyte‐specific *Setdb1* knockout (*Setdb1* HKO) mice on a C57BL/6N background were generated by a commercial supplier (Cyagen Biosciences, Suzhou, China) [17]. *Setdb1* Flox littermates served as the control cohort. A total of twenty‐four male *Setdb1* HKO mice were randomly distributed into four groups (*n* = 6 per group), and twelve *Setdb1* Flox mice were allocated into two groups (*n* = 6 per group). The six experimental groups were: *Setdb1* Flox + PF, *Setdb1* Flox + AF, *Setdb1* HKO + PF, *Setdb1* HKO + AF, *Setdb1* HKO + AF + AAV8‐null, and *Setdb1* HKO + AF + AAV8‐*Map1lc3b*. All mice were maintained on their respective diets for 8 weeks. For AAV‐treated groups, mice received a single tail vein injection of either AAV8‐null or AAV8‐*Map1lc3b* (1 × 10^1^ ^1^ PFU in 100 µL; TransheepBio, Shanghai, China) at week 2, with feeding continuing until the end of the study.

To evaluate the role of LC3B lipidation in vivo, an additional cohort of *Setdb1* HKO mice was generated. Twelve male *Setdb1* HKO mice were randomly divided into three groups (*n* = 4 per group): *Setdb1* HKO + AF + AAV8‐null, *Setdb1* HKO + AF + AAV8‐*Map1lc3b*, and *Setdb1* HKO + AF + AAV8‐*Map1lc3b‐G120A*. All mice were fed an alcohol diet for 8 weeks and received a single tail vein injection of the respective AAV8 vectors (1 × 10^1^ ^1^ PFU in 100 µL; TransheepBio, Shanghai, China) at week 2.

At the end of the feeding period, mice were fasted for 4 h prior to euthanasia. Anesthesia was induced with 1%–1.5% isoflurane in oxygen, and butorphanol tartrate was administered for analgesia. Blood samples were collected via the inferior vena cava, and liver tissues were harvested for further analysis.

### 2.4 Histological Analysis

### 2.5 Hematoxylin and Eosin (H&E) Staining

Fresh liver tissue was fixed in 4% formaldehyde in PBS for 24 h, embedded in paraffin, and sectioned into 5 µm slices. Sections were stained with H&E following standard protocols. Images were captured using a Motic EasyScan system (Motic Medical Diagnostic Systems Co., Ltd.).

### 2.6 Oil Red O Staining and Quantification

Oil Red O staining was performed to visualize lipid droplets in frozen liver sections or AML12 cells. Samples were washed twice with PBS, fixed in 4% paraformaldehyde for 15 min, and incubated with saturated Oil Red O solution (B1094, Applygen, Beijing, China) for 20 min. After washing with 60% isopropanol, nuclei were counterstained with hematoxylin (C1411, Applygen) for 30 s. Samples were mounted using glycerol gelatin solution (C1213, Applygen). Images were captured using a Motic EasyScan system. For quantification, five random fields per sample were analyzed at 200×magnification using ImageJ software (NIH, Bethesda, MD, USA), and the positively stained area was expressed as a percentage of total area.

### 2.7 Immunohistochemistry and Quantification

Immunohistochemistry was performed on 5 µm paraffin‐embedded liver sections. After deparaffinization and rehydration, antigen retrieval was performed by boiling in citrate buffer (pH 6.0) for 15 min. Endogenous peroxidase activity was blocked with 3% H_2_O_2_, and sections were incubated with primary antibodies against F4/80 (1:200, GB113373, Servicebio, Wuhan, China), MPO (1:200, GB150006, Servicebio, Wuhan, China), or Collagen I (1:200, GB11022, Servicebio, Wuhan, China) overnight at 4°C. Following incubation with HRP‐conjugated secondary antibodies, signals were visualized using DAB substrate. For quantification, positively stained cells (F4/80, MPO) or positively stained areas (collagen) were counted/measured in five random fields per sample at 400× magnification using ImageJ software.

### 2.8 Sirius Red Staining and Quantification

Liver sections were stained with Picro‐Sirius Red solution (0.1% Sirius Red in saturated picric acid) for 1 h at room temperature. After washing with acidified water, sections were dehydrated and mounted. Sirius Red‐positive areas were quantified in five random fields per sample at 200× magnification using ImageJ software and expressed as a percentage of total area.

### 2.9 TUNEL Assay

Apoptosis in liver sections was detected using the TMR (red) TUNEL Cell Apoptosis Detection Kit (G1502, Servicebio, Wuhan, China) according to the manufacturer's instructions. Briefly, paraffin‐embedded liver sections were deparaffinized, rehydrated, and permeabilized with proteinase K. After incubation with TUNEL reaction mixture at 37°C for 60 min, nuclei were counterstained with DAPI. TUNEL‐positive cells were counted in five random fields per sample at 400× magnification and expressed as the number per field.

### 2.10 Biochemical Assays

### 2.11 Serum Markers

Levels of serum triglycerides (TG), total cholesterol (TC), alanine aminotransferase (ALT), and aspartate aminotransferase (AST) were quantified using a Beckman AU5800 Chemistry System Analyzer.

### 2.12 Tissue and Cellular Lipid Measurement

Liver tissue (∼30 mg) or cultured AML12 cells were homogenized in Applygen Lysis Buffer (E1025, Applygen Technologies Inc., Beijing, China) following the manufacturer's guidelines. TG and TC levels were quantified using Applygen Assay Kits (E1025 for TG, E1026 for TC) with absorbance measured at 570 nM after 37°C incubation. Cellular TG/TC data were normalized to total protein concentration, while liver tissue values were adjusted by wet weight.

### 2.13 Cytokine ELISA

Serum levels of IL‐1β, IL‐6, CCL2, and TNF‐α were measured using commercial ELISA kits (IL‐1β: SYP‐M0026, IL‐6: SYP‐M0031QX, CCL2: SYP‐M0352, TNF‐α: SYP‐M0036, UpingBio Technology, Hangzhou, China) according to the manufacturer's instructions. Absorbance was measured at 450 nM using a microplate reader. Cytokine concentrations were calculated from standard curves generated with recombinant proteins provided in the kits.

### 2.14 RT‐qPCR

Total RNA was extracted from liver tissue and AML12 cells using RNAiso Plus reagent (9109, Takara, Otsu, Shiga, Japan). RNA concentration was measured using a Nanodrop One micro UV spectrophotometer (Thermo, San Jose, CA, USA). Reverse transcription was performed using a reverse transcription kit (D0401, HaiGene, Harbin, China) to generate cDNA. Quantitative PCR was performed using SYBR Green Realtime PCR Master Mix (QPK‐201, TOYOBO, Japan) on a QuantStudio3 Real‐Time PCR System (Thermo Fisher Scientific, Waltham, MA, USA). Primer sequences are listed in Table S2. 18S rRNA served as the internal reference. Relative gene expression was calculated using the 2^−^ΔΔCt method.

### 2.15 Western Blot Analysis

Liver tissue and AML12 cells were lysed using RIPA lysis buffer containing protease inhibitors (04693159001, Roche, Mannheim, BW, Germany) and phosphatase inhibitors (4906845001, Roche). Protein concentration was determined using the BCA protein assay kit. Equal amounts of protein were separated by 8%–12% SDS‐PAGE at 80 V and transferred to nitrocellulose membranes at 200 mA. Membranes were blocked with 5% skim milk in TBST for 1 h at room temperature, then incubated with primary antibodies (listed in Table S3) overnight at 4°C. After washing, membranes were incubated with fluorescently labeled secondary antibodies (LI‐COR) for 1 h at room temperature. Protein signals were detected using an Odyssey Infrared Imager (LI‐COR) and quantified using Image Studio software.

### 2.16 Immunoprecipitation

Immunoprecipitation was performed using the Co‐Immunoprecipitation Kit (P2177S, Beyotime Biotechnology) following the manufacturer's protocol. AML12 cells were lysed in RIPA buffer containing protease inhibitor cocktail. Cell lysates were incubated with SETDB1 antibody (11231‐1‐AP, 1:100, ProteinTech Group), ERG antibody (ab92513, 1:100, Abcam), or control IgG antibody overnight at 4°C with rotation. Protein A/G magnetic beads were added and incubated for 2 h at room temperature. After washing, bound proteins were eluted with 1× loading buffer by heating at 95°C for 5 min and analyzed by western blot.

### 2.17 Subcellular Fractionation

### 2.18 Membrane‐Cytosol Fractionation

Membrane and cytosolic fractions were prepared using the Membrane and Cytosol Protein Extraction Kit (P0033, Beyotime Biotechnology) according to the manufacturer's instructions. Briefly, cells were harvested, washed, and permeabilized with a detergent‐containing buffer. After centrifugation at 14000 × *g* for 15 min at 4°C, the supernatant containing cytosolic proteins was collected. The pellet was solubilized in membrane solubilization buffer and centrifuged to obtain the membrane fraction. ATP1A1 and α‐Tubulin were used as markers for membrane and cytosolic fractions, respectively.

### 2.19 Nuclear‐Cytosolic Fractionation

Nuclear and cytoplasmic fractions were prepared using the Nuclear and Cytoplasmic Protein Extraction Kit (P0028, Beyotime Biotechnology) according to the manufacturer's instructions. Cells were harvested, washed with PBS, and lysed in cytoplasmic extraction buffer on ice for 15 min. After centrifugation at 12000 × *g* for 5 min at 4°C, the supernatant was collected as the cytoplasmic fraction. The pellet was resuspended in nuclear extraction buffer, vortexed vigorously for 30 s every 10 min for 40 min, and centrifuged at 12000 × *g* for 10 min at 4°C to obtain the nuclear fraction. α‐Tubulin and Histone H3 were used as markers for cytoplasmic and nuclear fractions, respectively.

### 2.20 Autophagic Flux Analysis Using mCherry‐GFP‐LC3B Adenovirus

Cells were infected with Ad‐mCherry‐GFP‐LC3B adenovirus (Hanbio Biotechnology, Shanghai, China) at an MOI of 50 for 24 h. Cells were then treated with or without 100 nM Bafilomycin A1 (BAF1, S1413, Selleck Chemicals) for 6 h. After fixation with 4% paraformaldehyde for 15 min, coverslips were mounted, and images were acquired using a Laser Scanning Confocal Microscope ZEISS 800. For quantification, autolysosomes (red puncta) and autophagosomes (yellow puncta) were counted in at least 15 cells per group using ImageJ software. Puncta counts were normalized to mean fluorescence intensity to account for variations in infection efficiency.

### 2.21 Lipid Droplet Degradation Assay

For lipid droplet degradation analysis, AML12 cells were loaded with 100 µm oleic acid conjugated to BSA for 24 h, then washed and cultured in fresh medium for indicated time points (0, 3, 6, 12, 24 h). At each time point, cells were harvested, stained with 1 µg/mL BODIPY 493/503 (D3922, Invitrogen, Carlsbad, CA, USA) for 15 min at 37°C, and analyzed by flow cytometry. The mean fluorescence intensity at each time point was normalized to the 0 h time point to calculate the percentage of lipid droplet degradation.

### 2.22 Immunofluorescence and γH2AX Foci Quantification

Cells grown on coverslips were fixed with 4% paraformaldehyde for 15 min, permeabilized with 0.2% Triton X‐100 for 10 min, and blocked with 5% BSA for 1 h at room temperature. Cells were then incubated with anti‐γH2AX antibody (1:400, 9718, Cell Signaling Technology), anti‐Plin2 antibody (1:400, 15294‐1‐AP, ProteinTech Group), or anti‐LAMP2A antibody (1:400, ab18528, Abcam) overnight at 4°C, followed by incubation with Alexa Fluor 488 (1:500, A0428, Beyotime Biotechnology, Shanghai, China) or 594‐conjugated secondary antibodies (1:500, A0516, Beyotime Biotechnology, Shanghai, China) for 1 h at room temperature. Nuclei were counterstained with DAPI. Images were captured using a Laser Scanning Confocal Microscope ZEISS 800. For γH2AX quantification, foci were counted in at least 30 nuclei per group from three independent experiments using ImageJ software, and results were expressed as average foci per nucleus.

### 2.23 Cell Viability Assay

Cell proliferation was assessed using the CCK‐8 Kit (K1018, Apexbio, Guangzhou, China). Cells were seeded in 96‐well plates at a density of 5000 cells per well and cultured for 96 h. At indicated time points, 10 µL of CCK‐8 solution was added to each well and incubated for 2 h at 37°C. Absorbance was measured at 450 nM using a microplate reader. OD450 values were normalized to day 1 values, and relative cell viability was calculated by comparison with control groups.

### 2.24 Apoptosis Assay by Flow Cytometry

AML12 cells were seeded in 6‐well plates at a density of 1 × 10^6^ cells per well for 48–72 h. Cells were harvested using EDTA‐free trypsin, washed with PBS, and resuspended in 1× Binding Buffer. Cells were stained with 5 µL of Annexin V‐FITC and 5 µL of propidium iodide (PI) using the Annexin V/PI Apoptosis Detection Kit (K2003, Apexbio, Guangzhou, China) for 30 min in the dark. Stained cells were analyzed using a flow cytometer, and data were processed using FlowJo software.

### 2.25 Chromatin Immunoprecipitation (ChIP) Assay

ChIP analysis was performed using the ChIP Assay Kit (P2078, Beyotime Biotechnology, Shanghai, China) according to the manufacturer's instructions. AML12 cells were seeded in 10 cm dishes for 72 h. Formaldehyde was added directly to the medium to a final concentration of 1% and incubated at 37°C for 10 min to cross‐link proteins to DNA. Glycine was added to stop the fixation. Chromatin was sonicated to fragment DNA into 200–1000 bp fragments. Protein‐DNA complexes were immunoprecipitated using an ERG antibody (ab92513, Abcam) or normal mouse IgG (VD293456, Invitrogen, Carlsbad, CA, USA). After washing, cross‐links were reversed, and DNA was purified using a DNA purification kit (D0033, Beyotime Biotechnology, Shanghai, China). Purified DNA was analyzed by qPCR using primers targeting the *Map1lc3b* promoter region (listed in Table S2). Data were normalized to input chromatin.

### 2.26 Dual‐Luciferase Reporter Assay

The interaction between ERG and the *Map1lc3b* promoter was assessed using dual‐luciferase reporter assays. Wild‐type or mutant *Map1lc3b* promoter fragments were cloned into the pGL3‐basic vector upstream of the firefly luciferase gene. AML12 or HEK293T cells were seeded in 24‐well plates and co‐transfected with the reporter construct, an ERG expression vector, and a Renilla luciferase control vector using Lipofectamine 3000. After 48 h, cells were lysed, and luciferase activities were measured using the Dual‐Luciferase Reporter Assay Kit (E1980, Promega, Madison, WI, USA) on a Promega Glomax Multi Plus Detection Plate Reader. Firefly luciferase activity was normalized to Renilla luciferase activity.

### 2.27 DRIP‐qPCR

Genomic DNA was extracted from cultured cells using the Qiagen Blood & Cell Culture DNA Kit (13323, Qiagen, Hilden, Germany) with RNase A omitted to preserve native RNA:DNA hybrids. Ten micrograms of purified high‐molecular‐weight DNA were digested overnight at 37°C with a cocktail of restriction enzymes containing 10 µL each of SspI‐HF (F5576S), EcoRI‐HF (F5536S), XbaI (F5581S), and HinfI (F5593S) (LabLead, Beijing, China) in 100 µL of 1× CutSmart Buffer. Digested DNA was purified by phenol‐chloroform extraction and ethanol precipitation. For immunoprecipitation, 5 µg of digested DNA was incubated overnight at 4°C with 5 µg of S9.6 antibody (65983, Active Motif, Carlsbad, CA, USA) or normal mouse IgG (VD293456, Invitrogen, Carlsbad, CA, USA) as a negative control, followed by capture with protein A/G magnetic beads (88802, Thermo Fisher Scientific, Waltham, MA, USA). After washing, bound DNA was eluted, purified using the QIAquick PCR Purification Kit (28104, Qiagen, Hilden, Germany), and subjected to qPCR using primers targeting the *Actb*, *Polr3a*, and *Rbm19* loci (listed in Table S2). As a specificity control, parallel samples were treated with 5 U of RNase H (M0297S, New England Biolabs, Ipswich, MA, USA) at 37°C for 2 h prior to immunoprecipitation. Data were calculated as %Input and presented as enrichment fold over IgG.

### 2.28 Immuno‐Electron Microscopy

shNC and sh*Setdb1* AML12 cells were treated with culture medium containing 200 mM ethanol for 24 h, then fixed with immuno‐electron microscopy fixative (Servicebio, G1124) at 4°C for 2 h. After washing with 0.1 M phosphate buffer (pH 7.4), cells were embedded in 2% low‐melting agarose, dehydrated through a graded ethanol series, and infiltrated with LR White resin. Polymerization was performed at −20°C under UV light for 48 h. Ultrathin sections (70–80 nM) were cut using an ultramicrotome (Leica UC7) and collected on nickel grids. Sections were blocked with 1% BSA in TBS, then incubated with anti‐LC3B antibody (Novus Biologicals, NB600‐1384, 1:100) overnight at 4°C, followed by incubation with 12 nM gold‐conjugated secondary antibody (Jackson ImmunoResearch, 1:50) for 1 h at 37°C. Sections were stained with 2% uranyl acetate and examined under a transmission electron microscope (Hitachi HT7800). LC3B‐positive single‐membrane vesicles were quantified as the number of gold particle‐labeled single‐membrane structures per field at 8000× magnification.

### 2.29 Statistical Analysis

All data are expressed as mean ± standard deviation (SD). Statistical analyses were performed using GraphPad Prism 8.0 software (GraphPad Software, San Diego, CA, USA). For comparisons between two groups, Student's *t*‐test or Welch's *t*‐test was used depending on variance homogeneity. For non‐normally distributed data, the Mann‐Whitney test was applied. For multiple group comparisons, one‐way ANOVA was performed. For comparisons of multiple treatment groups against a single control group, Bonferroni's post‐hoc test was applied. For pairwise comparisons among all groups, Tukey's post‐hoc test was used. For comparisons with unequal variances, Tamhane's T2 post‐hoc test was applied. For time‐course experiments, two‐way ANOVA with Bonferroni's post‐hoc test or Tukey's post‐hoc test was performed. A *p*‐value of less than 0.05 was considered statistically significant.

## 3 Results

### 3.1 SETDB1 is Downregulated in ALD Mice Model and Protects Against Ethanol‐Induced Steatosis and Apoptosis

**FIGURE 1:**
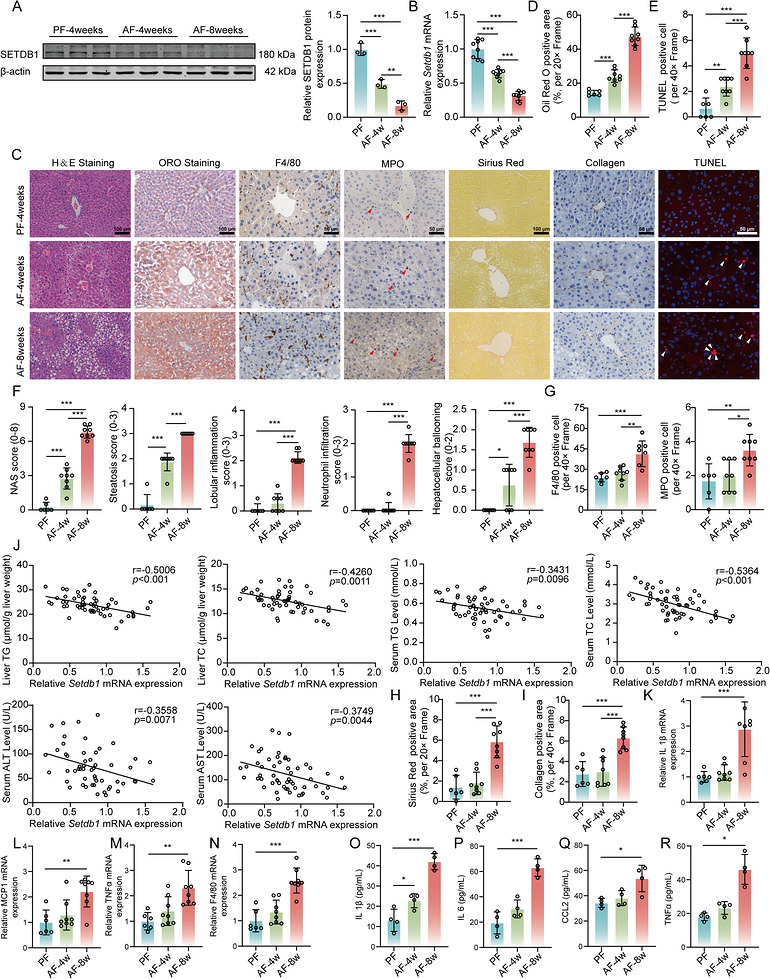
SETDB1 expression is progressively downregulated during ALD progression and correlates with liver injury. (A) Representative Western blot images and quantitative analysis of SETDB1 protein levels in liver tissues from PF‐4 W, AF‐4 W, and AF‐8 W mice. β‐actin served as loading control (*n* = 3 per group). (B) Relative mRNA expression of *Setdb1* in liver tissues from the indicated groups, determined by qPCR (*n* ≥ 6 per group). (C) Representative images of H&E staining, Oil Red O staining, F4/80 immunohistochemistry, MPO immunohistochemistry, Sirius Red staining, collagen immunohistochemistry, and TUNEL staining (red) of liver sections from PF‐4 W, AF‐4 W, and AF‐8 W mice. Scale bars, 100 µm (H&E, Oil Red O, Sirius Red) or 50 µm (F4/80, MPO, collagen, TUNEL). (D) Quantification of Oil Red O‐positive areas (% per 20× field, *n* ≥ 6 per group). (E) Quantification of TUNEL‐positive cells (per 40× field, *n* ≥ 6 per group). (F) Histological scoring of liver sections, including NAS score, steatosis score (0–3), lobular inflammation score (0–3), neutrophil infiltration score (0–3), and hepatocellular ballooning score (0–2), (*n* ≥ 6 per group). (G) Quantification of F4/80‐positive and MPO‐positive cells (per 40× field, *n* ≥ 6 per group). (H) Quantification of Sirius Red‐positive areas (% per 20× field, *n* ≥ 6 per group). (I) Quantification of collagen‐positive areas (% per 40× field, *n* ≥ 6 per group). (J) Correlation analyses between hepatic *Setdb1* mRNA levels and hepatic TG, hepatic TC, serum TG, serum TC, serum ALT, and serum AST levels in alcohol‐fed mice, Pearson's correlation coefficient was used for analysis (*n* = 56 per group). (K–N) Relative mRNA expression of *IL‐1β*, *MCP‐1*, *TNF‐α*, and *F4/80* in liver tissues from the indicated groups, determined by qPCR (*n* = 6 per group). (O–R) Serum levels of IL‐1β, IL‐6, CCL2, and TNF‐α measured by ELISA (*n* = 4 per group). All data are presented as mean ± SD. For comparisons between two groups, Student's *t*‐test was used. For multiple group comparisons, one‐way ANOVA followed by Tukey's post‐hoc test (A, D, E, F, G, H, I, K, L, M, N, O, P, Q, R), Tamhane's T2 post‐hoc test (B) was applied. *p < 0.05, **p < 0.01, ***p < 0.001; ns, not significant.

To investigate the role of SETDB1 in alcohol‐associated hepatic lipid deposition and inflammation, we employed a chronic alcohol feeding model by feeding C57BL/6N mice with the Lieber‐DeCarli liquid ethanol diet for 4 weeks (AF‐4 W) or 8 weeks (AF‐8 W), with an isocaloric pair‐fed group (PF‐4 W) serving as control. Compared with the PF group, the AF groups showed a time‐dependent downregulation of both hepatic SETDB1 protein abundance and mRNA expression, with AF‐8 W mice exhibiting the most pronounced reduction (Figure 1A,B), consistent with our previous findings [17].

Histological analysis revealed progressive liver injury upon alcohol feeding. Representative images of H&E, Oil Red O, F4/80 immunohistochemistry, MPO immunohistochemistry, Sirius Red staining, collagen immunohistochemistry, and TUNEL staining are shown in Figure 1C. Quantitative analysis demonstrated that Oil Red O‐positive areas (Figure 1D) and TUNEL‐positive cell numbers (Figure 1E) increased progressively across all groups, with statistical significance in each comparison. NAS scoring confirmed this trend (Figure 1F), with steatosis, hepatocellular ballooning, and total NAS scores showing significant differences among all groups. Notably, while lobular inflammation and neutrophil infiltration scores were comparable between PF‐4 W and AF‐4 W groups, they became significantly elevated after 8 weeks of alcohol feeding. Similarly, F4/80 and MPO immunostaining quantification revealed that macrophage and neutrophil infiltration were not increased at 4 weeks but were markedly elevated at 8 weeks compared to both PF‐4 W and AF‐4 W groups (Figure 1G). Fibrosis assessment by Sirius Red staining and collagen immunohistochemistry showed no significant differences between PF‐4 W and AF‐4 W groups. However, AF‐8 W mice exhibited a modest but statistically significant increase in fibrotic areas compared to both control groups (Figure 1H,I).

Correlation analysis in alcohol‐fed mice revealed that hepatic *Setdb1* mRNA levels were significantly inversely correlated with key pathological features of ALD, including hepatic TG and TC content, serum TG and TC levels, as well as serum ALT and AST activities (Figure 1J), indicating that *Setdb1* downregulation is closely associated with the severity of alcohol‐induced liver injury.

We next examined the inflammatory response in this model. qPCR analysis showed that hepatic mRNA levels of pro‐inflammatory cytokines and the macrophage marker F4/80 were not significantly upregulated at 4 weeks but became markedly elevated after 8 weeks of alcohol feeding compared to both PF‐4 W and AF‐4 W groups (Figure 1K–N). Consistently, serum levels of IL‐1β, IL‐6, CCL2, and TNF‐α measured by ELISA exhibited a similar pattern: no significant changes at 4 weeks but robust elevation at 8 weeks (Figure 1O–R). These results indicate that prolonged alcohol consumption leads to progressive downregulation of SETDB1, which correlates with aggravated hepatic steatosis, inflammation, and injury, suggesting a close association between SETDB1 downregulation and ALD progression.

**FIGURE 2:**
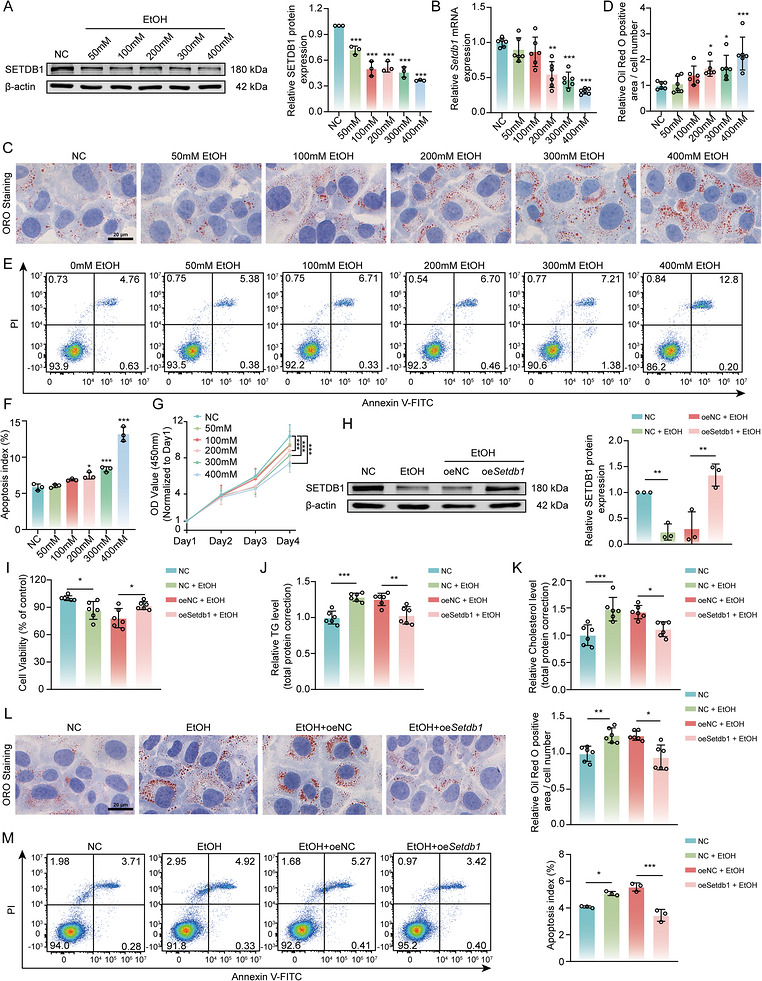
SETDB1 is downregulated by ethanol in AML12 cells and its overexpression alleviates ethanol‐induced lipotoxicity. (A) Representative Western blot images and quantitative analysis of SETDB1 protein levels in AML12 cells treated with indicated concentrations of ethanol for 72 h. β‐actin served as loading control (*n* = 3 per group). (B) Relative mRNA expression of *Setdb1* in AML12 cells treated with indicated concentrations of ethanol for 72 h, determined by qPCR (*n* = 6 per group). (C) Representative images of Oil Red O staining of AML12 cells treated with indicated concentrations of ethanol for 72 h. Scale bar, 20 µm. (D) Quantification of Oil Red O‐positive areas (*n* = 5 per group). (E) Flow cytometry analysis of apoptosis in AML12 cells treated with indicated concentrations of ethanol for 72 h. (F) Quantification of apoptotic cell percentage (*n* = 3 per group). (G) Cell viability assessed by CCK‐8 assay in AML12 cells treated with indicated concentrations of ethanol over 4 days (*n* = 6 per group). (H) Representative Western blot images and quantitative analysis of SETDB1 protein levels in stable oe*Setdb1* AML12 cell or empty vector (oeNC) prior to 200 mM ethanol treatment for 72 h. (I) Cell viability assessed by CCK‐8 assay in stable AML12 cells from the indicated groups (*n* = 3 per group). (J, K) Quantification of cellular TG and TC content in stable AML12 cells from the indicated groups (*n* = 6 per group). (L) Representative images and quantification of Oil Red O staining in stable AML12 cells from the indicated groups. Scale bar, 20 µm (*n* = 5 per group). (M) Flow cytometry analysis and quantification of apoptosis in stable AML12 cells from the indicated groups (*n* = 3 per group). All data are presented as mean ± SD. For multiple group comparisons, one‐way ANOVA followed by Bonferroni's post‐hoc test (A, D, F), Tukey's post‐hoc test (I, J, K, L, M), Tamhane's T2 post‐hoc test (B) or two‐way ANOVA for time‐course experiments (G) was applied. *p < 0.05, **p < 0.01, ***p < 0.001; ns, not significant.

To further validate the clinical relevance of SETDB1 downregulation in ALD and to establish an in vitro model for mechanistic studies, we treated AML12 murine hepatocytes with increasing concentrations of ethanol (50–400 mM) for 72 h. Western blot analysis revealed that SETDB1 protein levels decreased in a dose‐dependent manner upon ethanol exposure, with a significant reduction observed even at the lowest concentration of 50 mM (Figure 2A). Similarly, *Setdb1* mRNA levels exhibited a dose‐dependent decline, although statistical significance was reached only at concentrations of 200 mM and above (Figure 2B), suggesting that ethanol may regulate SETDB1 at both transcriptional and post‐transcriptional levels.

Consistent with the in vivo findings, ethanol treatment induced lipid accumulation in AML12 cells, as evidenced by Oil Red O staining. Quantification showed that Oil Red O‐positive areas became significantly increased at ethanol concentrations of 200 mM and higher (Figure 2C,D). Concurrently, flow cytometry analysis demonstrated that ethanol‐induced apoptosis was also evident from 200 mM onward (Figure 2E,F). Cell viability assessed by CCK‐8 assay over four days revealed a time‐ and dose‐dependent reduction, with significant decreases observed at 200 mM ethanol and above compared to control cells (Figure 2G).

Based on these findings, we selected 200 mM ethanol as the optimal concentration for subsequent gain‐of‐function experiments. Stable AML12 cell lines overexpressing *Setdb1* (oe*Setdb1*) or carrying empty vector (oeNC) were established using lentiviral transduction. Western blot analysis confirmed that *Setdb1* overexpression effectively reversed the ethanol‐induced reduction of SETDB1 protein levels (Figure 2H), validating the efficiency of our overexpression system. Functionally, CCK‐8 assay revealed that while ethanol exposure significantly impaired cell viability, *Setdb1* overexpression markedly attenuated this cytotoxic effect (Figure 2I). Moreover, ethanol‐induced accumulation of TG and TC was significantly ameliorated by *Setdb1* overexpression (Figure 2J,K), which was further corroborated by Oil Red O staining (Figure 2L). Consistently, flow cytometry analysis demonstrated that the elevated apoptotic rate caused by ethanol treatment was also reversed upon SETDB1 restoration (Figure 2M). Collectively, these results indicate that *Setdb1* overexpression protects hepatocytes from ethanol‐induced steatosis and apoptosis, positioning SETDB1 as a critical protective factor against alcoholic lipotoxicity.

### 3.2 SETDB1 Regulates Hepatic Steatosis, Apoptosis, and Cell Viability in AML12 Cells Under Ethanol‐Induced Stress

**FIGURE 3:**
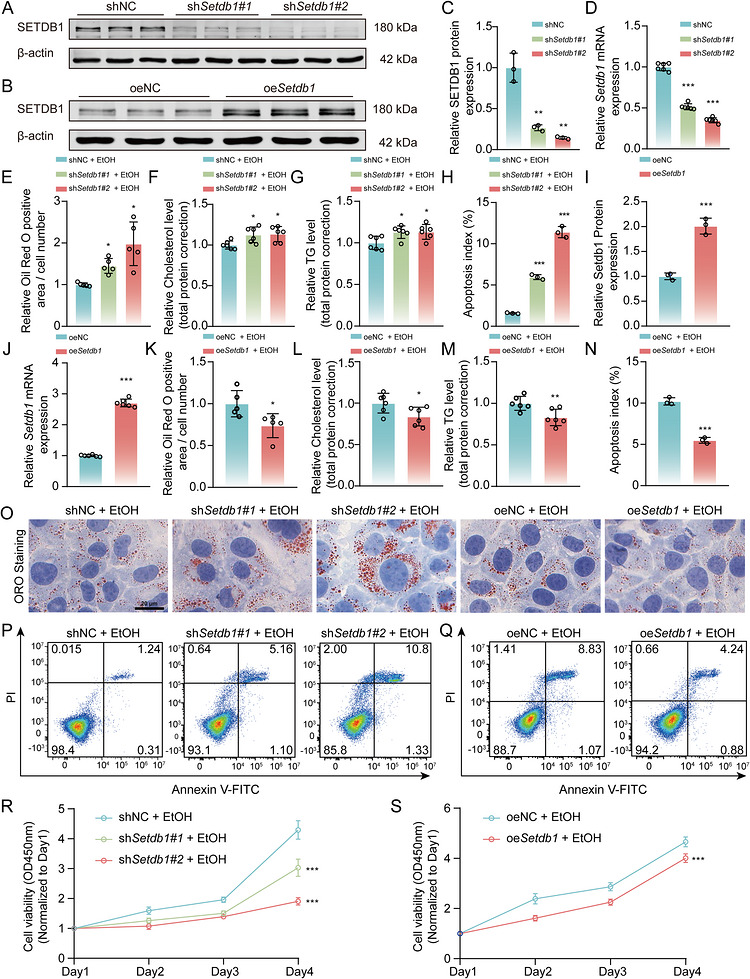
SETDB1 regulates hepatic steatosis, apoptosis, and cell viability in AML12 cells under ethanol‐induced stress. (A) Representative Western blot images and (C) quantitative analysis of SETDB1 protein levels in sh*Setdb1* AML12 cell lines (sh*Setdb1#1* and sh*Setdb1#2*) and control cells (shNC) under basal conditions. β‐actin served as loading control (*n* = 3 per group). (B) Representative Western blot images and (I) quantitative analysis of SETDB1 protein levels in stable oe*Setdb1* AML12 cell lines and control cells (oeNC), (*n* = 3 per group). (D) Relative mRNA expression of *Setdb1* in shNC and sh*Setdb1* cells under basal conditions, determined by qPCR (*n* = 6 per group). (J) Relative mRNA expression of *Setdb1* in oeNC and oe*Setdb1* cells (*n* = 6 per group). For functional analyses, all cells were treated with 200 mM ethanol for 24 h prior to assessment. (E, K) Quantification of Oil Red O‐positive areas in the indicated stable cell lines (*n* = 5 per group). (F, G) Quantification of cellular TG and TC content in shNC and sh*Setdb1* cells (*n* = 6 per group). (L, M) Quantification of cellular TG and TC content in oeNC and oe*Setdb1* cells (*n* = 6 per group). (H, N) Quantification of apoptotic cell percentage by flow cytometry in the indicated stable cell lines (*n* = 3 per group). (O) Representative images of Oil Red O staining in shNC, sh*Setdb1*, oeNC, and oe*Setdb1* cells. Scale bar, 20 µm. (P, Q) Representative flow cytometry plots of apoptosis in the indicated stable cell lines. (R, S) Cell viability assessed by CCK‐8 assay over 4 days in shNC versus sh*Setdb1* cells and oeNC versus oe*Setdb1* cells (*n* = 6 per group). All data are presented as mean ± SD. For comparisons between two groups, Student's *t*‐test (I, K, L, M, N) or Welch's *t*‐test (H, J) was used. For multiple group comparisons, one‐way ANOVA followed by Bonferroni's post‐hoc test (C, D, F, G, H), Tamhane's T2 post‐hoc test (E) or two‐way ANOVA for time‐course experiments (R, S) was applied. *p < 0.05, **p < 0.01, ***p < 0.001; ns, not significant.

To investigate the role of SETDB1 under ALD‐relevant pathological conditions, we established stable *Setdb1* knockdown (sh*Setdb1#1* and sh*Setdb1#2*) and overexpression (oe*Setdb1*) AML12 cell lines. Western blot and qPCR analyses confirmed successful modulation of SETDB1 expression at both protein and mRNA levels under basal conditions (Figure 3A–D,I–J), validating the efficiency of our stable cell lines.

We then examined the functional role of SETDB1 under ethanol‐induced stress. While our previous work demonstrated that SETDB1 regulates lipid accumulation under basal conditions [17], we herein sought to explore its role in the context of the “multiple‐hit” hypothesis, where alcohol serves as a secondary insult. Cells were treated with ethanol prior to functional analyses. *Setdb1* knockdown significantly exacerbated ethanol‐induced lipid accumulation, as evidenced by increased Oil Red O staining (Figure 3E,O) and elevated cellular TG and TC content (Figure 3F,G). Conversely, *Setdb1* overexpression markedly attenuated these effects (Figure 3K–M,O). Flow cytometry analysis demonstrated that *Setdb1* knockdown enhanced ethanol‐induced apoptosis (Figure 3H,P), while its overexpression reduced apoptotic cell numbers (Figure 3N,Q). Consistently, CCK‐8 assays showed that *Setdb1* knockdown impaired cell viability under ethanol stress, whereas its overexpression promoted cell survival (Figure 3R,S). Collectively, these results demonstrate that SETDB1 protects hepatocytes against alcohol‐induced lipotoxicity, supporting its role as a key modulator in ALD progression.

### 3.3 SETDB1 Modulates LAP Through Rubicon Membrane Translocation to Regulate Hepatic Lipid Accumulation and Apoptosis

**FIGURE 4:**
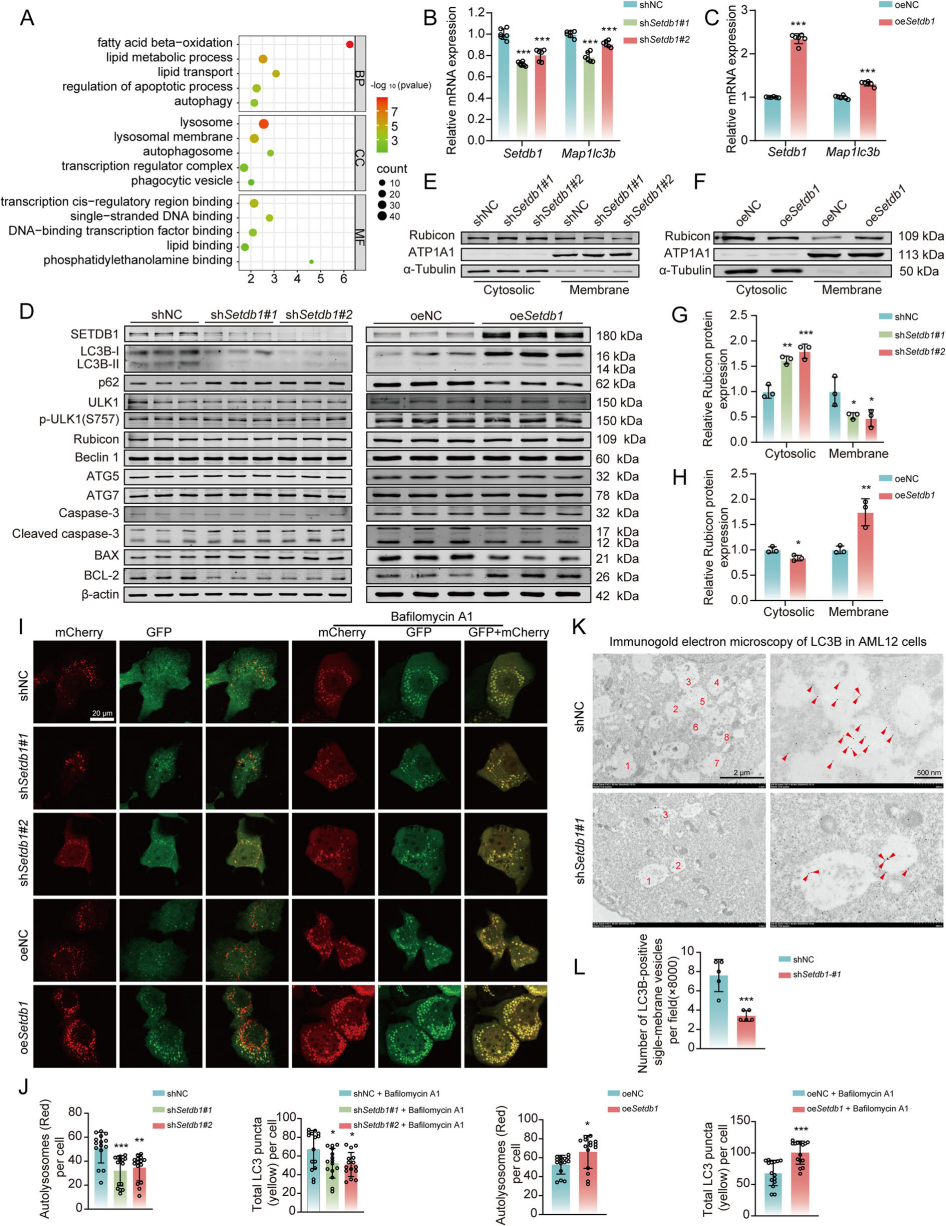
SETDB1 positively regulates *Map1lc3b* expression independent of H3K9me3‐mediated promoter silencing and modulates LAP through Rubicon membrane translocation. (A) Gene Ontology (GO) enrichment analysis of differentially expressed genes from sequencing data of si*Setdb1*‐treated AML12 cells compared to siNC controls (GSE225723). (B) Relative mRNA expression of *Setdb1* and *Map1lc3b* in shNC and sh*Setdb1* AML12 cells, determined by qPCR (*n* = 6 per group). (C) Relative mRNA expression of *Setdb1* and *Map1lc3b* in oeNC and oe*Setdb1* AML12 cells (*n* = 6 per group). (D) Representative Western blot images of SETDB1, LC3B‐I, LC3B‐II, and p62 in shNC, sh*Setdb1*, oeNC, and oe*Setdb1* AML12 cells. β‐actin served as loading control. (E, F) Representative Western blot images of Rubicon in membrane and cytosolic fractions isolated from shNC versus sh*Setdb1* cells (E) and oeNC versus oe*Setdb1* cells (F). ATP1A1 and α‐Tubulin served as markers for membrane and cytosolic fractions, respectively. (G, H) Quantitative analysis of Rubicon distribution in membrane and cytosolic fractions from the indicated cell lines (*n* = 3 per group). (I) Representative confocal images of AML12 cells infected with Ad‐mCherry‐GFP‐LC3B, treated with or without Bafilomycin A1 (BAF1, 100 nM, 6 h). Scale bar, 20 µm. (J) Quantification of autolysosomes (red puncta) per cell in the absence of BAF1 and total LC3 puncta (yellow puncta) per cell upon BAF1 treatment, normalized to shNC control (*n* = 15 per group). (K, L) Representative immuno‐EM images of LC3B localization in ethanol‐treated AML12 cells. Both shNC control cells (K) and sh*Setdb1#1* knockdown cells (L) were exposed to 200 mM ethanol treatment. LC3B‐conjugated gold particles (black dots, arrows) were enriched in single‐membrane phagosomes containing lipid droplets in control cells. *Setdb1* knockdown significantly reduced the number of LC3B‐positive single‐membrane structures. LC3B‐positive single‐membrane vesicles were counted per field at 8000× magnification (*n* = 5 per group). Scale bars: 2 µm (overview) and 500 nM (enlarged view). All data are presented as mean ± SD. For comparisons between two groups, Student's *t*‐test (C, H, J, L) or Welch's *t*‐test (J) was used. For multiple group comparisons, one‐way ANOVA followed by Bonferroni's post‐hoc test (B, G, J) or Tamhane's T2 post‐hoc test (J) was applied. *p < 0.05, **p < 0.01, ***p < 0.001; ns, not significant.

To investigate the molecular mechanisms underlying SETDB1‐mediated regulation of lipid deposition and apoptosis in ALD, we performed GO enrichment analysis on differentially expressed genes identified from sequencing data of si*Setdb1*‐treated AML12 cells compared to siNC controls (GSE225723). This analysis revealed that SETDB1 downregulation significantly affected multiple pathways, including autophagy and apoptosis (Figure 4A). Notably, our previous work [17] has systematically characterized the roles of classical lipid metabolism pathways in SETDB1‐mediated hepatic steatosis. Building upon these findings, we herein focused on the autophagy pathway, which emerged as a core functional cluster in our GO analysis and plays critical roles in ALD progression [31, 32].

To identify autophagy‐related genes regulated by SETDB1, we examined a panel of core autophagy components in stable *Setdb1* knockdown and overexpression AML12 cells. Among all genes examined, *Map1lc3b* was the only one exhibiting consistent and significant regulation by SETDB1, with its expression positively correlated with SETDB1 levels (Figure S1A,B). This regulatory relationship was further validated by qPCR analysis, demonstrating that *Setdb1* knockdown significantly reduced while overexpression markedly enhanced *Map1lc3b* mRNA levels (Figure 4B,C). Consistently, Western blot analysis revealed that SETDB1 positively regulated LC3B‐I and LC3B‐II protein levels (Figure 4D).

To determine whether this regulation occurs through SETDB1's canonical H3K9me3‐mediated transcriptional repression, we analyzed the enrichment of H3K9me3 in the promoter regions of autophagy‐related genes using the GSE141104 dataset. Interestingly, while promoters of *Atg5*, *Atg16L1*, *Atg12*, *Atg9b*, and *Lamp2* displayed varying degrees of H3K9me3 enrichment, the *Map1lc3b* promoter showed negligible H3K9me3 signals (Figure S2), suggesting that SETDB1 regulates *Map1lc3b* through a mechanism distinct from its conventional H3K9me3‐dependent transcriptional silencing.

Quantitative analysis of multiple autophagy‐related proteins (Figure 4D,S3A,B) showed that SETDB1 manipulation did not affect the expression of ULK1, p‐ULK (S757), Rubicon, Beclin1, ATG5, or ATG7. Notably, while the LC3B‐II/LC3B‐I ratio remained unchanged, the LC3B‐II/p62 ratio was significantly decreased in sh*Setdb1* cells and increased in oe*Setdb1* cells (Figure 4D,S3A,B), indicating that SETDB1 modulates LC3B lipidation and p62 turnover. Given that core autophagy machinery components were unaffected, we hypothesized that SETDB1 might regulate a non‐canonical autophagy pathway. Apoptosis‐related markers further confirmed that SETDB1 negatively regulates apoptotic cell death, with cleaved caspase‐3 and BAX levels reduced and BCL‐2 increased upon SETDB1 overexpression, with opposite effects observed upon knockdown (Figure 4D,S3A,B).

To further characterize the impact of SETDB1 on LC3B lipidation and p62 turnover, we performed Western blot analysis in the presence of Bafilomycin A1 (BAF1), a lysosomal inhibitor. In sh*Setdb1* cells, BAF1 treatment revealed that *Setdb1* knockdown significantly impaired the accumulation of LC3B‐II upon lysosomal blockade, while further increasing p62 levels (Figure S4A,C,D). Consistently, the LC3B‐II/LC3B‐I ratio remained unchanged across groups, whereas the LC3B‐II/p62 ratio was significantly reduced in sh*Setdb1* cells both with and without BAF1 treatment (Figure S4C,D). Similar results were obtained in oe*Setdb1* cells, where *Setdb1* overexpression enhanced LC3B‐II accumulation upon BAF1 treatment and reduced p62 levels, leading to an increased LC3B‐II/p62 ratio (Figure S4B,E,F). These findings indicate that SETDB1 modulates LC3B lipidation and p62 degradation in a lysosome‐dependent manner, consistent with the regulation of an autophagy‐related process.

Given that Rubicon total protein expression was unchanged, yet the LC3B‐II/p62 ratio was significantly altered by SETDB1 manipulation, we hypothesized that SETDB1 might affect Rubicon subcellular localization—a key feature of LAP but not canonical autophagy. To test this hypothesis, we examined Rubicon distribution by membrane‐cytosol fractionation. Remarkably, membrane‐cytosol fractionation revealed that *Setdb1* knockdown significantly reduced Rubicon levels in the membrane fraction while increasing its cytosolic distribution (Figure 4E,G). Conversely, *Setdb1* overexpression promoted Rubicon membrane translocation (Figure 4F,H). These results demonstrate that SETDB1 regulates Rubicon membrane localization without affecting its total protein expression. While our analysis reflects total membrane fractions, these changes are likely to influence the pool of Rubicon available for recruitment to nascent phagosomes, thereby potentially contributing to LAP regulation.

To functionally assess the impact of SETDB1 on LAP, we performed autophagic flux assays using dual‐labeled recombinant adenovirus (Ad‐mCherry‐GFP‐LC3B) with or without BAF1 treatment. Representative images are shown in Figure 4I. After normalizing for infection efficiency, we found that SETDB1 knockdown significantly reduced both red puncta (acidic compartments) under basal conditions and total LC3‐positive puncta (yellow puncta) upon BAF1 treatment (Figure 4J). Conversely, *Setdb1* overexpression markedly increased both parameters (Figure 4J). To further confirm that SETDB1 regulates LAP rather than canonical autophagy, we performed immuno‐electron microscopy to visualize LC3B‐associated membrane structures. As shown in Figure 4K,L, immuno‐electron microscopy (immuno‐EM) analysis revealed that in ethanol‐treated shNC control cells, LC3B gold particles were predominantly associated with single‐membrane phagosomes surrounding lipid droplets or other cytoplasmic contents. Double‐membrane autophagosomes were only occasionally detected under our experimental conditions. Importantly, under the same ethanol treatment, *Setdb1* knockdown (sh*Setdb1#1*) significantly reduced the number of LC3B‐positive single‐membrane structures. Together, these findings indicate that SETDB1 regulates Rubicon membrane localization and LC3B‐positive single‐membrane structure formation, consistent with its role in modulating LAP activity.

Having established that SETDB1 regulates LAP activity, we next asked whether LAP mediates the protective effects of SETDB1 against ethanol‐induced lipotoxicity. To determine whether these protective effects are dependent on lysosomal function (a hallmark of LAP), we performed rescue experiments using BAF1 in oe*Setdb1* cells exposed to ethanol. Oil Red O staining revealed that *Setdb1* overexpression significantly attenuated ethanol‐induced lipid accumulation, while co‐treatment with BAF1 abolished this protective effect (Figure S5A,B). Consistently, flow cytometry analysis demonstrated that *Setdb1* overexpression reduced ethanol‐induced apoptosis, which was also reversed by BAF1 treatment (Figure S5C,D). Moreover, quantification of cellular TG and TC content confirmed that the reduction in lipid accumulation conferred by *Setdb1* overexpression was abrogated upon BAF1 treatment (Figure S5E,F). Collectively, these results demonstrate that SETDB1 protects against ethanol‐induced steatosis and apoptosis in a lysosome‐dependent manner, further supporting its role in regulating LAP‐mediated lipid droplet degradation and apoptotic cell clearance.

### 3.4 LC3B Restoration Rescues SETDB1 Deficiency‐Induced Lipotoxicity and Apoptosis in a LAP‐Dependent Manner

**FIGURE 5:**
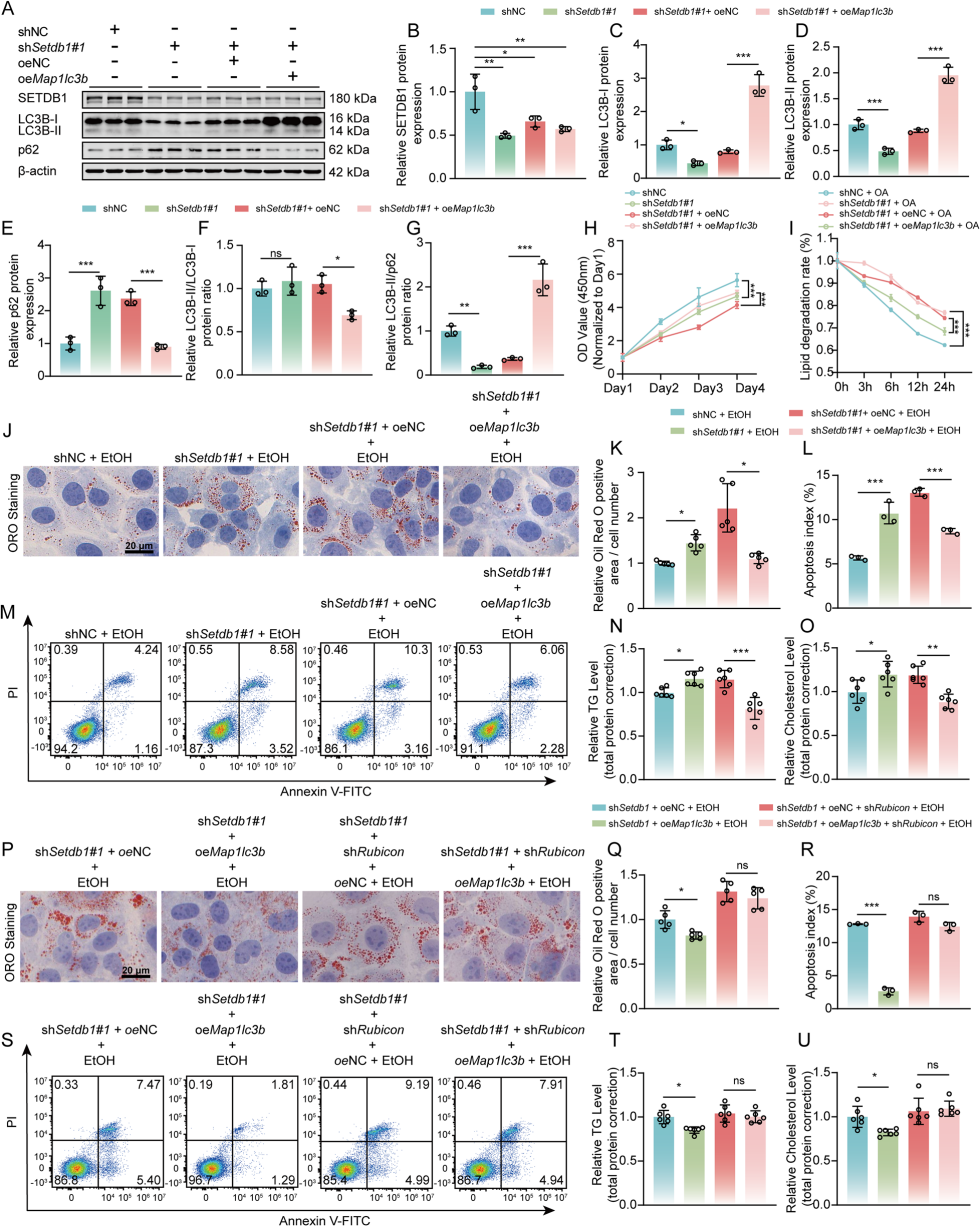
LC3B restoration rescues SETDB1 deficiency‐induced lipotoxicity and apoptosis in a Rubicon‐dependent manner. (A) Representative Western blot images of SETDB1, LC3B‐I, LC3B‐II, and p62 in the indicated groups: shNC, sh*Setdb1#1*, sh*Setdb1#1* + oeNC, and sh*Setdb1#1* + oe*Map1lc3b*. β‐actin served as loading control. (B–E) Quantitative analysis of SETDB1 (B), LC3B‐I (C), LC3B‐II (D), and p62 (E) protein levels (*n* = 3 per group). (F, G) Quantification of LC3B‐II/LC3B‐I ratio (F) and LC3B‐II/p62 ratio (G) (*n* = 3 per group). (H) Cell viability assessed by CCK‐8 assay over 4 days in the indicated groups (*n* = 6 per group). (I) Kinetics of lipid droplet degradation following oleic acid loading, assessed by BODIPY staining and flow cytometry, normalized to 0 h time point (*n* = 6 per group). (J) Representative images of Oil Red O staining in the indicated groups. Scale bar, 20 µm. (K) Quantification of Oil Red O‐positive areas (*n* = 5 per group).  (L) Quantification of apoptotic cell percentage (*n* = 3 per group). (M) Representative flow cytometry plots of apoptosis. (N, O) Quantification of cellular TG and total cholesterol TC content (*n* = 6 per group). (P) Representative images of Oil Red O staining in the indicated groups with or without *Rubicon* knockdown. Scale bar, 20 µm. (Q) Quantification of Oil Red O‐positive areas (*n* = 5 per group). (R) Quantification of apoptotic cell percentage (n = 3 per group). (S) Representative flow cytometry plots of apoptosis. (T, U) Quantification of cellular TG and TC content (*n* = 6 per group). All data are presented as mean ± SD. For multiple group comparisons, one‐way ANOVA followed by Tukey's post‐hoc test (B, C, D, E, F, G, L, N, O, Q, R, T, U), Tamhane's T2 post‐hoc test (K) or two‐way ANOVA by Tukey's post‐hoc test (H, I) for time‐course experiments was applied. *p < 0.05, **p < 0.01, ***p < 0.001; ns, not significant.

To determine whether LC3B mediates the protective effects of SETDB1 against ethanol‐induced lipotoxicity, we performed rescue experiments in sh*Setdb1* AML12 cells. Western blot analysis confirmed successful *Setdb1* knockdown and *Map1lc3b* overexpression in the indicated groups (Figure 5A). Quantitative analysis showed that *Setdb1* knockdown significantly reduced LC3B‐I and LC3B‐II levels while increasing p62 accumulation, all of which were reversed upon *Map1lc3b* overexpression (Figure 5B–E). Notably, while the LC3B‐II/LC3B‐I ratio remained unchanged upon *Setdb1* knockdown, it was significantly decreased upon *Map1lc3b* overexpression (Figure 5F), suggesting that exogenous LC3B primarily exists in its non‐lipidated form or that lipidation efficiency is limited by the availability of upstream LAP machinery components. In contrast, the LC3B‐II/p62 ratio was significantly reduced by *Setdb1* knockdown and restored by *Map1lc3b* overexpression (Figure 5G), indicating improved LAP‐mediated cargo degradation.

Under ethanol treatment, functional assays revealed that *Setdb1* knockdown impaired cell viability, which was rescued by *Map1lc3b* overexpression (Figure 5H). Using BODIPY staining coupled with flow cytometry to monitor lipid droplet clearance over time, we found that *Setdb1* knockdown significantly inhibited lipid degradation following oleic acid loading, an effect that was reversed upon LC3B restoration (Figure 5I). Consistently, Oil Red O staining (Figure 5J,K), apoptosis analysis (Figure 5L,M), and quantification of cellular TG and TC content (Figure 5N,O) all demonstrated that *Map1lc3b* overexpression effectively rescued SETDB1 deficiency‐induced lipid accumulation and apoptosis.

To confirm that these protective effects are independent of canonical autophagy, we employed the ULK1 inhibitor SBI‐0206965. In *Setdb1* knockdown cells exposed to ethanol, *Map1lc3b* overexpression significantly reduced lipid accumulation and apoptosis regardless of SBI‐0206965 treatment (Figure S6A–F), indicating that the observed effects are mediated through ULK1‐independent pathways consistent with LAP.

We next investigated whether Rubicon, a key LAP regulator, is required for LC3B‐mediated rescue. In *Setdb1* knockdown cells, *Map1lc3b* overexpression significantly reduced lipid accumulation and apoptosis, but these protective effects were completely abolished upon *Rubicon* knockdown (Figure 5P–U), demonstrating that Rubicon is essential for LC3B function in this context.

**FIGURE 6:**
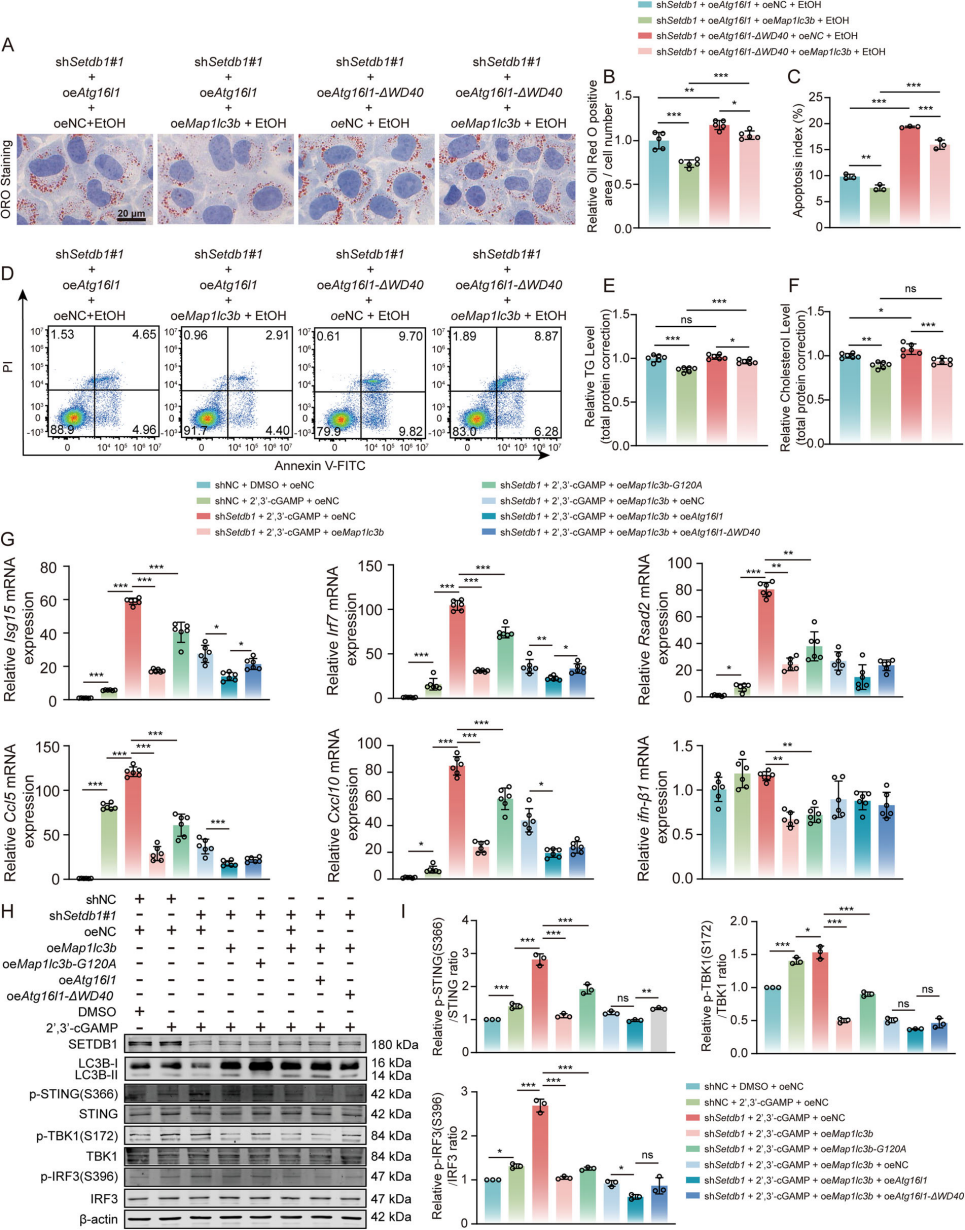
SETDB1 deficiency exacerbates cGAS‐STING activation through impaired LAP. (A) Representative images of Oil Red O staining in the indicated groups: sh*Setdb1#1* + oe*Atg16l1* + oeNC, sh*Setdb1#1* + oe*Atg16l1* + oe*Map1lc3b*, sh*Setdb1#1* + oe*Atg16l1‐ΔWD40* + oeNC, and sh*Setdb1#1* + oe*Atg16l1‐ΔWD40* + oe*Map1lc3b*, all treated with 200 mM ethanol. Scale bar, 20 µm. (B) Quantification of Oil Red O‐positive areas (*n* = 5 per group). (C) Quantification of apoptotic cell percentage (n = 3 per group). (D) Representative flow cytometry plots of apoptosis. (E, F) Quantification of cellular TG and TC content (*n* = 6 per group). (G) qPCR analysis of cGAS target gene expression (*Isg15*, *Irf7*, *Rsad2*, *Ccl5*, *Cxcl10*, *Ifnβ1*) in eight experimental groups following 2′3′‐cGAMP stimulation. 18S served as internal control (*n* = 6 per group). (H) Representative Western blot images of SETDB1, LC3B‐I, LC3B‐II, p‐STING (S366), STING, p‐TBK1 (S172), TBK1, p‐IRF3 (S396), IRF3, and β‐actin in the indicated groups. (I) Quantification of p‐STING/STING, p‐TBK1/TBK1, and p‐IRF3/IRF3 ratios (*n* = 6 per group). All data are presented as mean ± SD. For multiple group comparisons, one‐way ANOVA followed by Tukey's post‐hoc test (B, C, E, F, G, I) or Tamhane's T2 post‐hoc test (G) was applied. *p < 0.05, **p < 0.01, ***p < 0.001; ns, not significant.

Given that ATG16L1 and its WD40 domain are critical for LAP [33], we examined whether the WD40 domain is required for LC3B‐mediated rescue. Overexpression of wild‐type ATG16L1 enhanced the protective effects of LC3B, whereas the WD40 deletion mutant (ATG16L1‐ΔWD40) failed to do so (Figure 6A–F). Notably, cells expressing ATG16L1‐ΔWD40 exhibited increased lipid accumulation and apoptosis compared to those expressing wild‐type ATG16L1, even in the presence of exogenous LC3B (Figure 6A–F), indicating that the WD40 domain is essential for LC3B‐mediated LAP function downstream of SETDB1.

### 3.5 SETDB1 Deficiency Exacerbates cGAS‐STING Activation Through Impaired LAP

To explore the impact of SETDB1 on inflammatory signaling, we examined cGAS‐STING pathway activation in response to 2′3′‐cGAMP stimulation across eight experimental groups. qPCR analysis revealed that while *Ifn‐β1* mRNA levels remained unchanged across groups, other cGAS target genes—including *Isg15*, *Irf7*, *Rsad2*, *Ccl5*, and *Cxcl10—*were significantly upregulated upon 2′3′‐cGAMP stimulation (Figure 6G). Notably, *Setdb1* knockdown dramatically exacerbated this response, with *Ccl5* and *Irf7* expression increasing over 100‐fold compared to controls (Figure 6G), highlighting the potent anti‐inflammatory role of SETDB1.

*Map1lc3b* overexpression significantly attenuated the hyper‐inflammatory response in SETDB1‐deficient AML12 cells (Figure 6G). Interestingly, the lipidation‐deficient LC3B‐G120A mutant also partially rescued inflammation, albeit to a lesser extent than wild‐type LC3B (Figure 6G), suggesting that LC3B exerts anti‐inflammatory effects through both lipidation‐dependent and independent mechanisms. Furthermore, wild‐type ATG16L1 co‐overexpression enhanced the rescue effect of LC3B, while the WD40 deletion mutant abolished this enhancement (Figure 6G), indicating that the WD40 domain is required for optimal suppression of cGAS‐STING signaling.

Western blot analysis of STING pathway phosphorylation confirmed these findings (Figure 6H,I). *Setdb1* knockdown significantly increased p‐STING, p‐TBK1, and p‐IRF3 levels upon 2′3′‐cGAMP stimulation, all of which were attenuated by *Map1lc3b* overexpression. The LC3B‐G120A mutant showed partial rescue with variable statistical significance across phosphorylation sites. Wild‐type ATG16L1 co‐overexpression enhanced suppression, reaching statistical significance for p‐IRF3, while the WD40 deletion mutant tended to reverse this effect without achieving statistical significance for most comparisons (Figure 6H,I). Quantification of SETDB1, LC3B‐I, and LC3B‐II levels confirmed successful manipulation of these proteins across groups (Figure S7A–C), with the LC3B‐G120A mutant showing no detectable LC3B‐II, confirming its lipidation deficiency.

Collectively, these results demonstrate that SETDB1 deficiency exacerbates cGAS‐STING activation. LC3B suppresses this hyper‐inflammatory response through both lipidation‐dependent and lipidation‐independent mechanisms, as evidenced by the partial rescue effect of the lipidation‐deficient LC3B‐G120A mutant. The lipidation‐dependent function requires the ATG16L1 WD40 domain, confirming the involvement of LAP.

### 3.6 SETDB1 Regulates *Map1lc3b* Transcription by Functioning as a Transcriptional Cofactor of ERG

**FIGURE 7:**
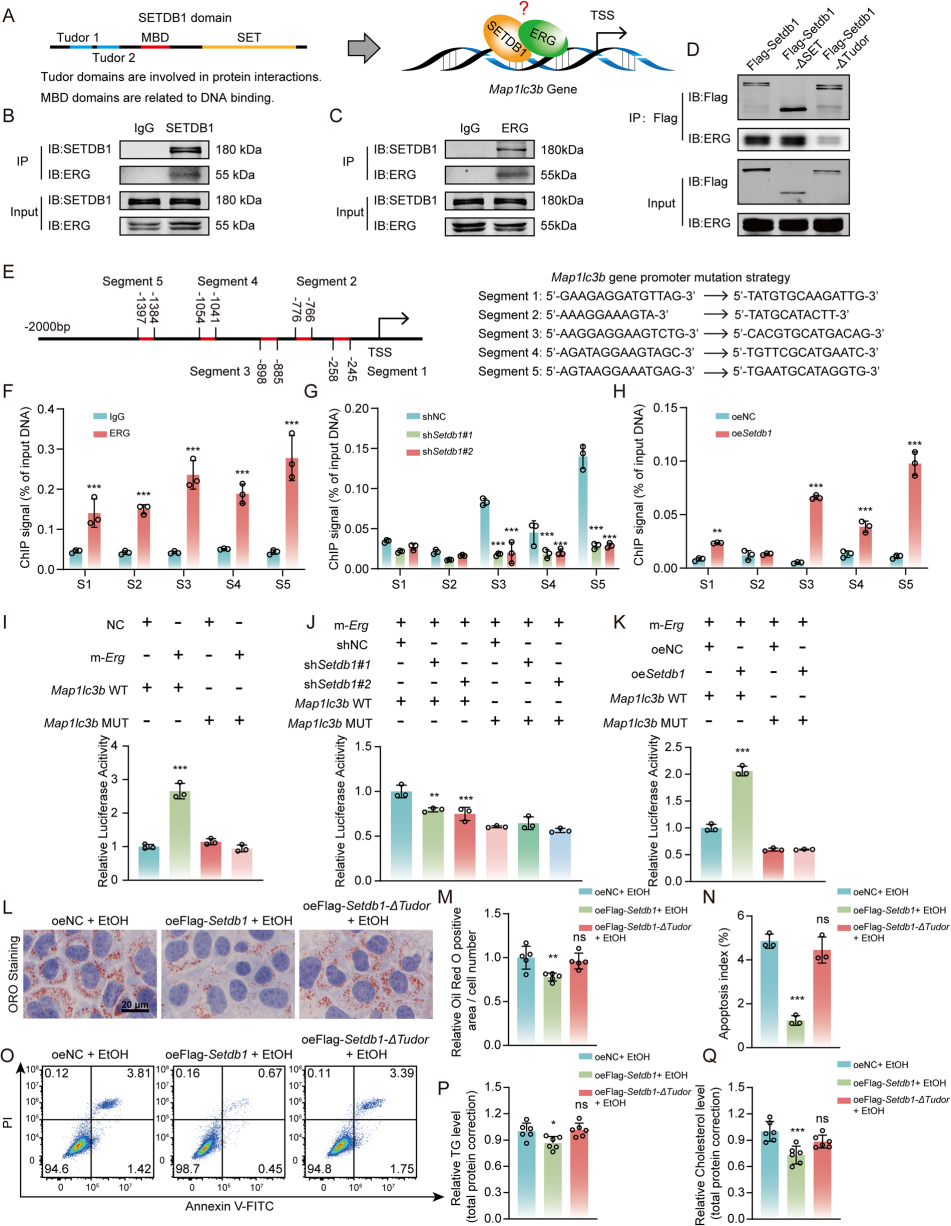
SETDB1 regulates *Map1lc3b* transcription by functioning as a transcriptional cofactor of ERG. (A) Schematic diagram depicting the hypothesis that SETDB1 acts as a transcriptional cofactor for ERG to regulate *Map1lc3b* transcription. (B, C) Co‐immunoprecipitation assays demonstrating the endogenous interaction between SETDB1 and ERG in AML12 cells. (D) Exogenous co‐IP assays using Flag‐tagged SETDB1 full‐length, ΔSET, and ΔTudor mutants, showing that deletion of the Tudor domain abolishes the SETDB1‐ERG interaction. (E) Schematic representation of the mouse *Map1lc3b* promoter region with predicted ERG‐binding sites (segments 1–5) selected for ChIP‐qPCR analysis and the mutation strategy for dual‐luciferase reporter assays. (F) ChIP‐qPCR analysis of ERG enrichment at the five indicated sites within the *Map1lc3b* promoter in AML12 cells, compared to IgG controls (*n* = 3 per group). (G, H) ChIP‐qPCR analysis of ERG enrichment at the *Map1lc3b* promoter in sh*Setdb1* (G) and oe*Setdb1* (H) AML12 cells (*n* = 3 per group). (I) Dual‐luciferase reporter assays in HEK293T cells transfected with ERG and wild‐type or mutant *Map1lc3b* promoter constructs. (J, K) Dual‐luciferase reporter assays in sh*Setdb1* (J) and oe*Setdb1* (K) AML12 cells transfected with ERG and wild‐type or mutant *Map1lc3b* promoter constructs (*n* = 3 per group). (L) Representative images of Oil Red O staining in AML12 cells treated with 200 mM ethanol and transfected with empty vector, Flag‐*Setdb1*, or Flag‐*Setdb1*‐ΔTudor. Scale bar, 20 µm. (M) Quantification of Oil Red O‐positive areas (*n* = 5 per group). (N) Quantification of apoptotic cell percentage (*n* = 3 per group). (O) Representative flow cytometry plots of apoptosis in the indicated groups. (P, Q) Quantification of cellular TG and TC content (*n* = 6 per group). All data are presented as mean ± SD. For comparisons between two groups, Student's *t*‐test (F, H) was used. For multiple group comparisons, one‐way ANOVA followed by Bonferroni's post‐hoc test (G), Tukey's post‐hoc test (I, J, K, M, P, Q) or Tamhane's T2 post‐hoc test (N) was applied. *p < 0.05, **p < 0.01, ***p < 0.001; ns, not significant.

Structural analysis revealed that SETDB1 contains a methyl‐CpG‐binding domain, indicating its DNA‐binding capability, although SETDB1 itself does not function as a traditional transcription factor. Given our findings that SETDB1 regulates *Map1lc3b* at both mRNA and protein levels (Figure 4B–D), and existing evidence showing SETDB1 can interact with the transcription factor ERG to participate in gene regulation [34, 35], we hypothesized that SETDB1 modulates *Map1lc3b* transcription by serving as a transcriptional cofactor for ERG (Figure 7A).

To test this hypothesis, we first confirmed the physical interaction between endogenous SETDB1 and ERG in AML12 hepatocytes through co‐immunoprecipitation assays (Figure 7B,C). To map the domain required for this interaction, we generated two SETDB1 deletion mutants lacking the SET domain (ΔSET) or the Tudor domain (ΔTudor). Exogenous co‐IP experiments revealed that deletion of the Tudor domain, but not the SET domain, abolished the SETDB1‐ERG interaction (Figure 7D), identifying the Tudor domain as critical for binding to ERG.

Bioinformatic analysis using the JASPAR database predicted multiple ERG‐binding elements in the *Map1lc3b* promoter region, from which we selected the top five conserved sites for experimental validation (Figure 7E and Table S4). ChIP‐qPCR assays in AML12 cells revealed significant ERG enrichment at multiple loci within the *Map1lc3b* promoter compared to IgG controls, particularly at segments 3, 4, and 5 (Figure 7F). Notably, ERG binding was markedly attenuated at these sites in sh*Setdb1* cells (Figure 7G), while *Setdb1* overexpression enhanced ERG recruitment to the *Map1lc3b* promoter (Figure 7H), demonstrating that SETDB1 facilitates ERG occupancy at its target sites.

To further elucidate the functional relevance of this interaction, we generated mutations in all five ERG‐binding sites within the *Map1lc3b* promoter and performed dual‐luciferase reporter assays. In HEK293T cells, *Erg* overexpression significantly enhanced *Map1lc3b* promoter activity, an effect that was completely abolished by mutation of the ERG‐binding sites (Figure 7I). In AML12 cells, ERG‐mediated transcriptional activation of *Map1lc3b* was markedly attenuated upon *Setdb1* knockdown (Figure 7J) and enhanced by *Setdb1* overexpression (Figure 7K). Importantly, these regulatory effects were abrogated when the ERG‐binding sites were mutated (Figure 7J,K), confirming that SETDB1 modulates *Map1lc3b* transcription specifically through ERG‐dependent mechanisms.

To further validate the role of ERG in regulating *Map1lc3b* expression, we established stable *Erg* knockdown and overexpression AML12 cell lines. Western blot and qPCR analyses confirmed that ERG positively regulates both LC3B protein and *Map1lc3b* mRNA levels (Figure S8A–D), consistent with its function as a transcription factor upstream of *Map1lc3b*. Functional assays revealed that *Erg* knockdown exacerbated ethanol‐induced lipid accumulation and apoptosis, while *Erg* overexpression protected against these effects (Figure S8E–J), further supporting the importance of the SETDB1‐ERG axis in regulating cellular responses to ethanol stress.

We next investigated whether the Tudor domain‐mediated SETDB1‐ERG interaction is functionally relevant for SETDB1's protective effects against ethanol‐induced lipotoxicity. Oil Red O staining demonstrated that wild‐type *Setdb1* overexpression attenuated ethanol‐induced lipid accumulation, whereas the ΔTudor mutant showed no such rescue (Figure 7L,M). Apoptosis analysis revealed that while wild‐type *Setdb1* overexpression significantly reduced apoptosis, the Tudor domain deletion mutant (ΔTudor) failed to exert this protective effect (Figure 7N,O). Consistently, quantification of cellular TG and TC content confirmed that the protective effects of SETDB1 were lost upon deletion of the Tudor domain (Figure 7P,Q), indicating that the SETDB1‐ERG interaction is essential for its function in mitigating alcoholic lipotoxicity.

### 3.7 LC3B Ameliorates Alcoholic Hepatic Steatosis and Injury in *Setdb1* HKO Mice

**FIGURE 8:**
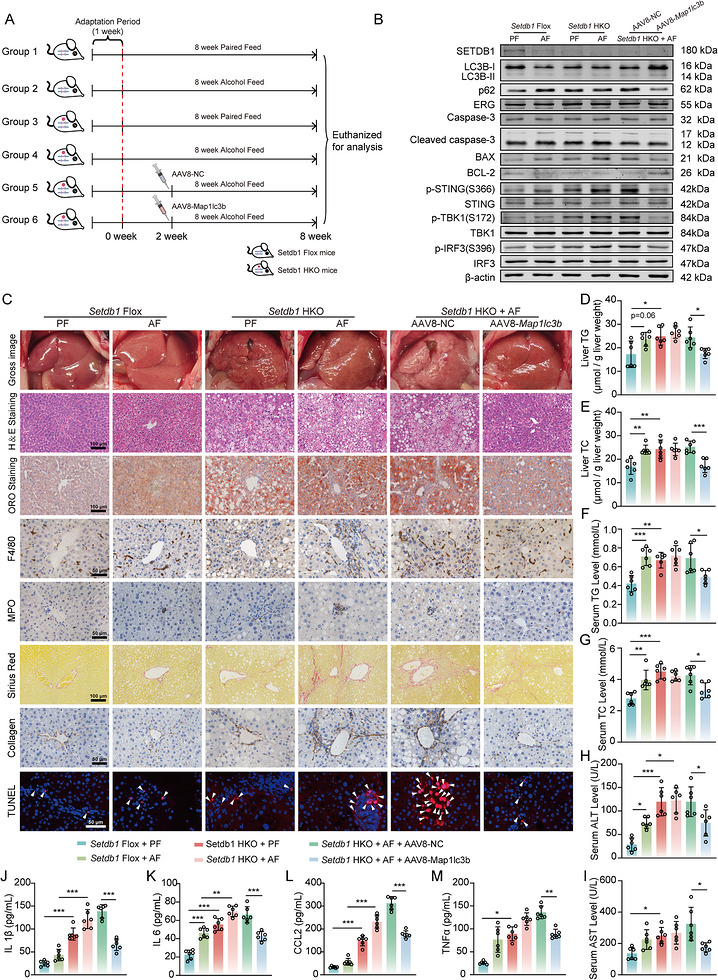
*Map1lc3b* overexpression ameliorates alcoholic liver injury in *Setdb1* HKO mice. (A) Schematic diagram of the experimental design showing the six groups: *Setdb1* Flox+PF, *Setdb1* Flox+AF, *Setdb1* HKO+PF, *Setdb1* HKO+AF, *Setdb1* HKO+AF+AAV8‐null, and *Setdb1* HKO+AF+AAV8‐*Map1lc3b*. (B) Representative Western blot images of SETDB1, LC3B‐I, LC3B‐II, p62, BAX, BCL‐2, caspase‐3, cleaved caspase‐3, p‐STING, STING, p‐TBK1, TBK1, p‐IRF3, IRF3 and ERG in liver tissues from the indicated groups. β‐actin served as loading control. (C) Representative images of gross liver morphology, H&E staining, Oil Red O staining, F4/80 immunohistochemistry, MPO immunohistochemistry, Sirius Red staining, collagen immunohistochemistry, and TUNEL staining of liver sections from the six groups. (D‐G) Quantification of hepatic and serum TG and TC levels (*n* = 6 per group). (H, I) Serum ALT and AST activities (*n* = 6 per group). (J–M) Serum levels of IL‐1β, IL‐6, CCL2, and TNF‐α measured by ELISA (*n* = 6 per group). All data are presented as mean ± SD. For multiple group comparisons, one‐way ANOVA followed by Tukey's post‐hoc test (D, E, F, G, H, I, J, K, L) or Tamhane's T2 post‐hoc test (M) was applied. *p < 0.05, **p < 0.01, ***p < 0.001; ns, not significant.

To investigate the therapeutic potential of LC3B in SETDB1 deficiency‐associated alcoholic liver disease, we utilized hepatocyte‐specific *Setdb1* knockout (*Setdb1* HKO) mice and their *Setdb1* Flox littermate controls. Mice were subjected to pair‐fed (PF) or Lieber‐DeCarli ethanol diet (AF) feeding, and *Setdb1* HKO mice with established ethanol‐induced liver injury were further treated with AAV8‐null or AAV8‐*Map1lc3b* via tail vein injection (Figure 8A). Western blot analysis confirmed successful *Setdb1* knockout and *Map1lc3b* overexpression, along with changes in p62, apoptosis markers, and ERG levels across the six experimental groups (Figure 8B and Figure S9A). Quantitative analysis revealed that SETDB1 protein was significantly reduced in all *Setdb1* HKO groups compared to *Setdb1* Flox PF controls (Figure S9A). LC3B‐I and LC3B‐II levels were decreased in *Setdb1* HKO mice and restored upon AAV8‐*Map1lc3b* treatment. Notably, while the LC3B‐II/LC3B‐I ratio was only reduced in *Setdb1* Flox AF mice, the LC3B‐II/p62 ratio was significantly decreased in all *Setdb1* HKO groups and rescued by *Map1lc3b* overexpression (Figure S9A). Apoptosis‐related markers, including BAX, BCL‐2, and cleaved caspase‐3/caspase‐3 ratio, confirmed that SETDB1 deficiency promotes apoptosis, which was attenuated by LC3B restoration (Figure S9A).

We next examined whether SETDB1 deficiency activates the cGAS‐STING pathway in vivo and whether LC3B restoration suppresses this response. Western blot analysis revealed that SETDB1 deficiency markedly increased the phosphorylation of STING, TBK1, and IRF3 compared to *Setdb1* Flox PF controls, indicating sustained pathway activation. Alcohol feeding further exacerbated this effect. Notably, AAV8‐*Map1lc3b* treatment significantly attenuated the phosphorylation levels of these key signaling molecules in alcohol‐fed *Setdb1* HKO mice, whereas AAV8‐null treatment had no effect (Figure 8B and Figure S9A).

Gross morphological examination, H&E staining, Oil Red O staining, F4/80 and MPO immunohistochemistry, Sirius Red staining, collagen immunohistochemistry, and TUNEL assays were performed to assess liver pathology (Figure 8C). Quantitative analysis demonstrated that *Setdb1* Flox AF mice exhibited moderate hepatic steatosis and inflammation compared to *Setdb1* Flox PF controls, whereas *Setdb1* HKO mice developed pronounced lipid accumulation, inflammatory infiltration, and hepatocellular damage even under PF dietary conditions (35% fat), which were further exacerbated by alcohol feeding (Figure S9B–H). Notably, unlike the mild fibrosis observed in wild‐type mice after 8 weeks of alcohol feeding (Figure 1), *Setdb1* HKO mice displayed markedly increased hepatic fibrosis under PF conditions alone, with additional aggravation upon alcohol challenge (Figure 8C and Figure S9F,G). These findings indicate that SETDB1 deficiency dramatically enhances disease susceptibility to nutritional stressors, accelerating the progression from simple steatosis to steatohepatitis and fibrosis in ALD.

Importantly, AAV8‐*Map1lc3b* treatment significantly ameliorated hepatic steatosis, inflammation, apoptosis, and fibrosis in alcohol‐fed *Setdb1* HKO mice, as evidenced by reduced Oil Red O‐positive areas, decreased F4/80 and MPO‐positive cell infiltration, attenuated TUNEL‐positive cells, and diminished collagen deposition (Figure 8C and Figure S9C–H). Consistently, hepatic and serum TG and TC levels were elevated in *Setdb1* HKO mice and reduced upon *Map1lc3b* overexpression (Figure 8D–G). Serum ALT and AST activities, markers of liver injury, followed a similar pattern, with significant increases in *Setdb1* HKO mice and partial rescue by AAV8‐*Map1lc3b* treatment (Figure 8H,I).

We next examined the inflammatory response by measuring serum cytokine levels. ELISA analysis revealed that IL‐1β, IL‐6, CCL2, and TNF‐α were significantly elevated in *Setdb1* HKO mice compared to *Setdb1* Flox PF controls, and these elevations were markedly attenuated by *Map1lc3b* overexpression (Figure 8J–M). Notably, IL‐1β and CCL2 showed significant increases specifically in *Setdb1* HKO groups regardless of dietary condition, highlighting the critical role of SETDB1 in restraining hepatic inflammation.

Collectively, these results demonstrate that SETDB1 deficiency accelerates ALD progression from simple steatosis to steatohepatitis and fibrosis, and that *Map1lc3b* overexpression effectively ameliorates this exacerbated liver injury, supporting the therapeutic potential of targeting the SETDB1‐LC3B axis in ALD.

### 3.8 LC3B Protects Against ALD Through Lipidation‐Dependent LAP and Lipidation‐Independent Nuclear Stabilization

**FIGURE 9:**
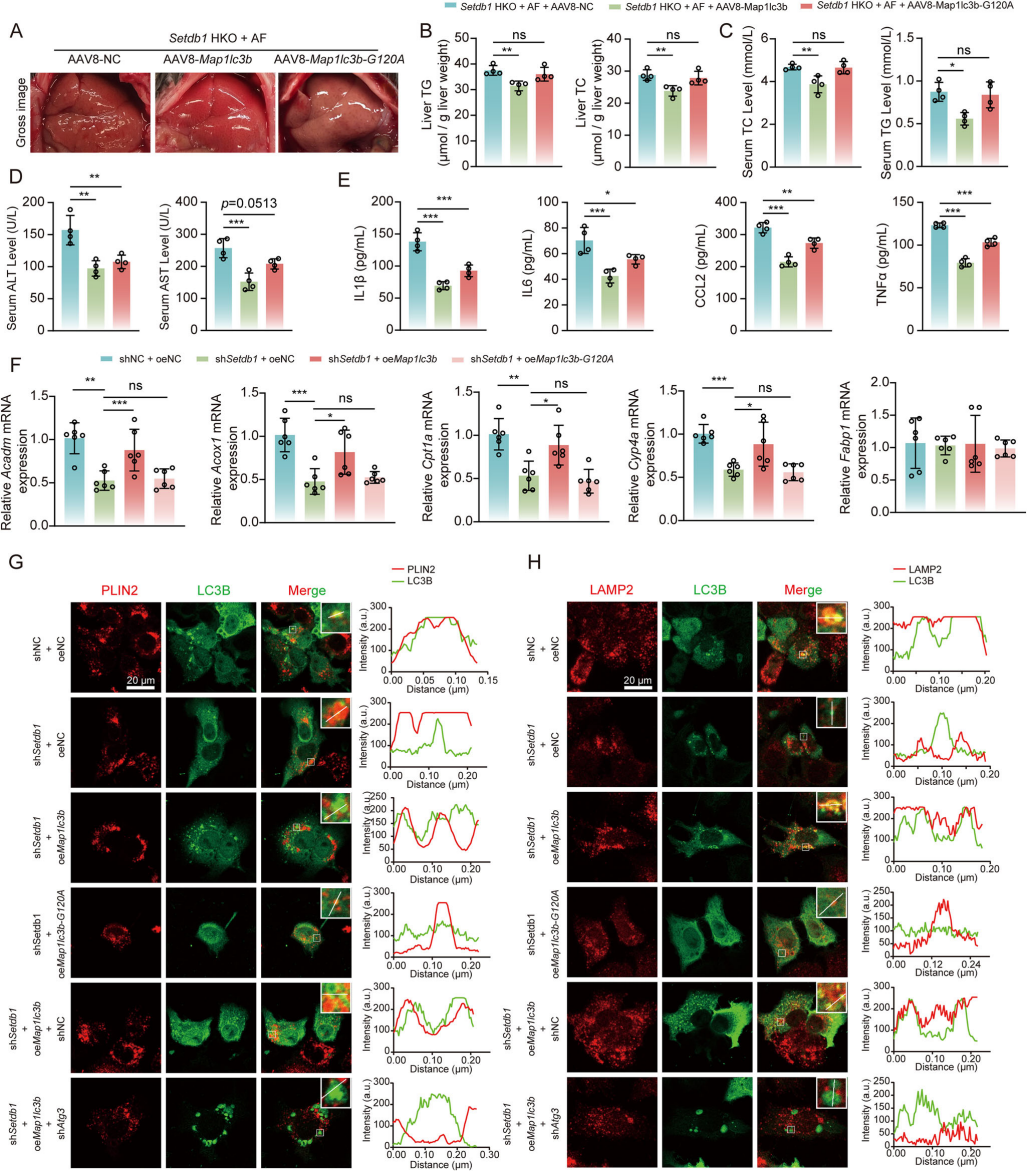
LC3B‐G120A mutant partially rescues inflammation but not steatosis through lipidation‐independent nuclear functions. (A) Representative gross images of livers from *Setdb*1 HKO mice fed an alcohol diet and treated with AAV8‐null, AAV8*‐Map1lc3b*, or AAV8*‐Map1lc3b‐G120A*. (B‐C) Quantification of hepatic and serum TG and TC levels in the indicated groups (n = 4 per group). (D) Serum ALT and AST activities (*n* = 4 per group). (E) Serum levels of IL‐1β, IL‐6, CCL2, and TNF‐α measured by ELISA (*n* = 4 per group). (F) qPCR analysis of PPARα target gene expression (*Acadm*, *Acox1*, *Cpt1α*, *Cyp4a*, *Fabp1*) in the indicated AML12 cell groups (*n* = 6 per group). (G‐H) Representative immunofluorescence images and quantification of LC3B co‐localization with Plin2 (G) and LAMP2 (H) in the indicated groups. Scale bars, 20 µm. All data are presented as mean ± SD. For multiple group comparisons, one‐way ANOVA followed by Tukey's post‐hoc test (B, C, D, E, F) was applied. *p < 0.05, **p < 0.01, ***p < 0.001; ns, not significant.

To investigate whether the lipidation‐deficient LC3B‐G120A mutant retains protective effects in vivo, we administered AAV8‐*Map1lc3b* or AAV8‐*Map1lc3b‐G120A* to alcohol‐fed *Setdb1* HKO mice. Gross morphological examination of liver tissues revealed that AAV8‐*Map1lc3b* treatment markedly ameliorated alcohol‐induced liver injury, while the LC3B‐G120A mutant showed partial improvement with noticeable residual lipid deposition (Figure 9A). Consistently, hepatic and serum TG and TC levels were significantly reduced by wild‐type LC3B overexpression but remained unchanged in LC3B‐G120A ‐treated mice compared to controls (Figure 9B,C). Serum ALT and AST activities followed a similar pattern, with wild‐type LC3B significantly attenuating liver injury markers, whereas the LC3B‐G120A mutant showed a modest but non‐significant trend toward reduction (Figure 9D). Interestingly, ELISA analysis of serum inflammatory cytokines revealed that both wild‐type LC3B and the LC3B‐G120A mutant significantly reduced IL‐1β, IL‐6, CCL2, and TNF‐α levels compared to controls, although the LC3B‐G120A mutant exhibited a weaker effect than wild‐type LC3B (Figure 9E). These results suggest that LC3B‐G120A retains partial anti‐inflammatory capacity despite its inability to ameliorate steatosis.

To further explore this dichotomy, we examined PPARα target gene expression in *Setdb1* knockdown AML12 cells. PPARα is a master regulator of hepatic lipid metabolism that promotes fatty acid oxidation and exerts anti‐inflammatory effects, while also enhancing autophagy‐lysosome pathways to facilitate lipid droplet clearance [36, 37]. qPCR analysis revealed that *Acadm*, *Acox1*, *Cpt1α*, and *Cyp4a* mRNA levels were significantly reduced upon *Setdb1* knockdown and restored by wild‐type LC3B overexpression, whereas the LC3B‐G120A mutant failed to rescue their expression (Figure 9F). *Fabp1* levels remained unchanged across groups, consistent with its known tissue‐specific regulation. These data indicate that LC3B‐G120A cannot restore PPARα‐dependent lipid metabolism gene expression, correlating with its inability to ameliorate steatosis.

Immunofluorescence analysis of LC3B co‐localization with the lipid droplet marker Plin2 and the lysosomal marker LAMP2 revealed that *Setdb1* knockdown significantly reduced their co‐localization, indicating impaired LAP‐mediated lipid droplet targeting (Figure 9G,H). Wild‐type LC3B overexpression restored this co‐localization, whereas the LC3B‐G120A mutant failed to do so. Notably, *Atg3* knockdown, which disrupts LC3 lipidation, also abolished LC3B‐Plin2 and LC3B‐LAMP2 co‐localization, further confirming that the LC3B‐G120A mutant's inability to rescue steatosis stems from its lipidation deficiency (Figure 9G,H).

**FIGURE 10:**
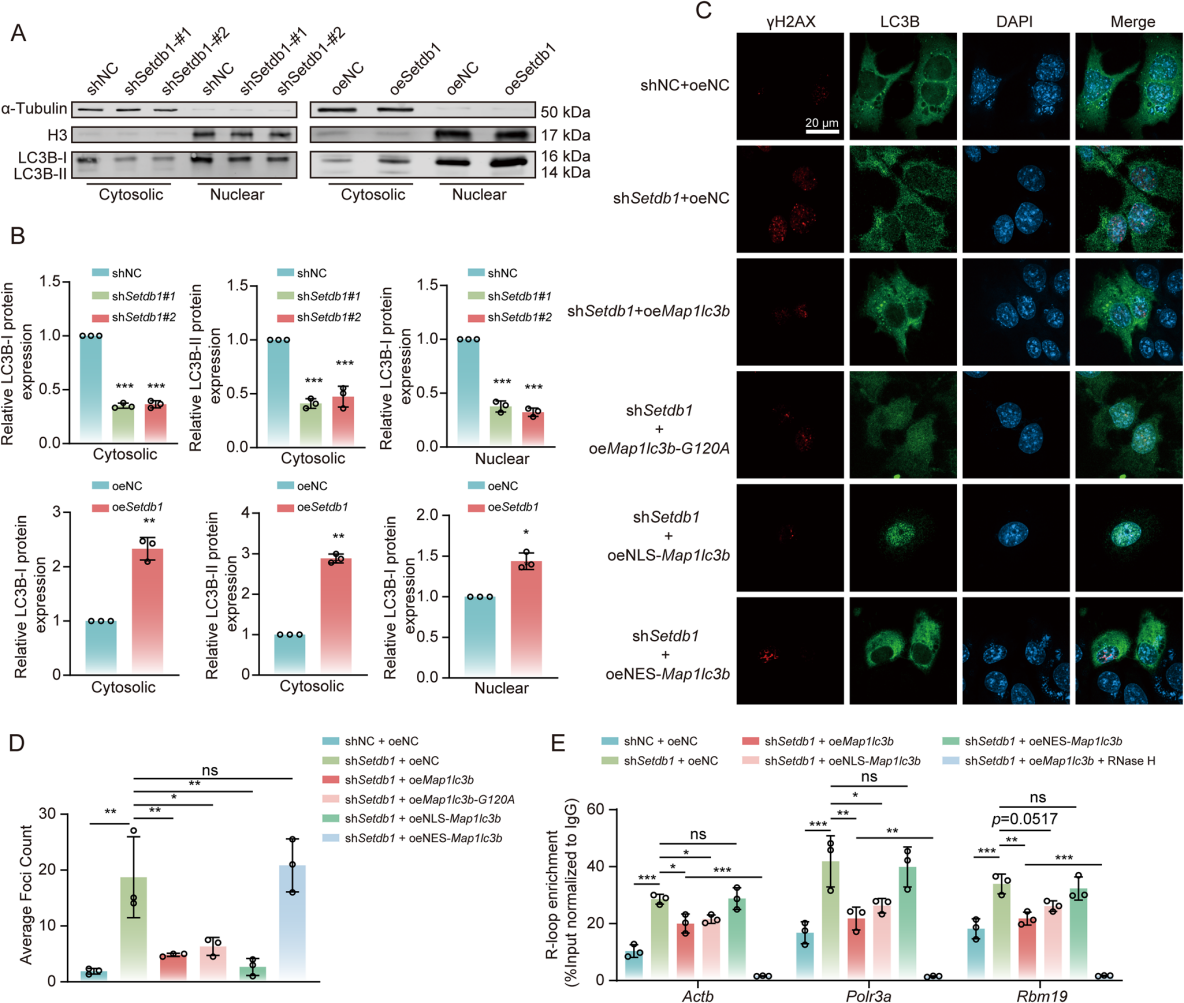
Nuclear LC3B mediates SETDB1‐dependent suppression of cGAS‐STING activation through maintaining genomic stability. (A) Representative Western blot images of LC3B‐I and LC3B‐II in cytosolic and nuclear fractions from shNC, sh*Setdb1*, oeNC, and oe*Setdb1* AML12 cells. α‐Tubulin and Histone H3 served as markers for cytosolic and nuclear fractions, respectively. (B) Quantitative analysis of LC3B‐I and LC3B‐II levels in cytosolic and nuclear fractions(*n* = 3 per group). (C) Representative immunofluorescence images of γH2AX foci in the indicated groups: shNC+oeNC, sh*Setdb1*+oeNC, sh*Setdb1*+oe*Map1lc3b*, sh*Setdb1*+oe*Map1lc3b*‐G120A, sh*Setdb1*+oeNLS‐*Map1lc3b*, and sh*Setdb1*+oeNES‐*Map1lc3b*. Scale bar, 20 µm. (D) Quantification of γH2AX foci per nucleus. (E) DRIP‐qPCR analysis of R‐loop enrichment at *Actb*, *Polr3a*, and *Rbm19* loci in the indicated groups (*n* = 3 per group). RNase H treatment served as a specificity control. All data are presented as mean ± SD. For comparisons between two groups, Student's *t*‐test (B) or Welch's *t*‐test (B) was used. For multiple group comparisons, one‐way ANOVA followed by Bonferroni's post‐hoc test (B), Tukey's post‐hoc test (D, E) was applied. *p < 0.05, **p < 0.01, ***p < 0.001; ns, not significant.

Given that the LC3B‐G120A mutant partially rescued inflammation without affecting steatosis, and emerging evidence suggests that LC3B can translocate to the nucleus and exert non‐canonical functions independent of its lipidation [38, 39], we hypothesized that non‐lipidated LC3B might enter the nucleus to regulate genomic stability and subsequent cGAS‐STING activation. To test this, we performed nuclear‐cytosolic fractionation in sh*Setdb1* and oe*Setdb1* AML12 cells. Western blot analysis revealed that *Setdb1* knockdown significantly reduced both cytosolic and nuclear LC3B‐I levels, as well as cytosolic LC3B‐II, while *Setdb1* overexpression increased these levels (Figure 10A,B). These results demonstrate that nuclear LC3B is regulated by SETDB1, independent of its lipidation status.

We next assessed nuclear stability by quantifying γH2AX foci, a marker of DNA damage. *Setdb1* knockdown markedly increased γH2AX foci number, which was significantly reduced by overexpression of wild‐type LC3B, the G120A mutant, and NLS‐tagged LC3B (targeted to the nucleus) (Figure 10C,D). In contrast, NES‐tagged LC3B (excluded from the nucleus) failed to rescue the increased γH2AX foci caused by SETDB1 deficiency (Figure 10C,D). These findings indicate that nuclear localization, rather than lipidation, is required for LC3B‐mediated maintenance of genomic stability.

To further validate this mechanism, we performed DRIP‐qPCR to assess R‐loop levels at three genomic loci—*Actb*, *Polr3a*, and *Rbm19—*which are known to be prone to R‐loop formation and whose instability can activate the cGAS‐STING pathway through cytosolic DNA leakage [27, 31]. *Setdb1* knockdown significantly increased R‐loop enrichment at all three loci. This accumulation was rescued by overexpression of wild‐type LC3B and NLS‐tagged LC3B, but not by NES‐tagged LC3B (Figure 10E). RNase H treatment, which specifically degrades the RNA moiety of R‐loops, abolished the enrichment in all groups, confirming the specificity of our DRIP‐qPCR signals (Figure 10E).

Collectively, these results demonstrate that LC3B exerts dual functions in ALD: lipidation‐dependent LAP in the cytoplasm mediates lipid droplet clearance and steatosis regulation, while lipidation‐independent nuclear localization maintains genomic stability by limiting R‐loop accumulation, thereby suppressing cGAS‐STING‐driven inflammation. The inability of NES‐tagged LC3B to rescue γH2AX foci and R‐loop accumulation further confirms that nuclear localization is essential for this protective mechanism.

## 4 Discussion

**FIGURE 11:**
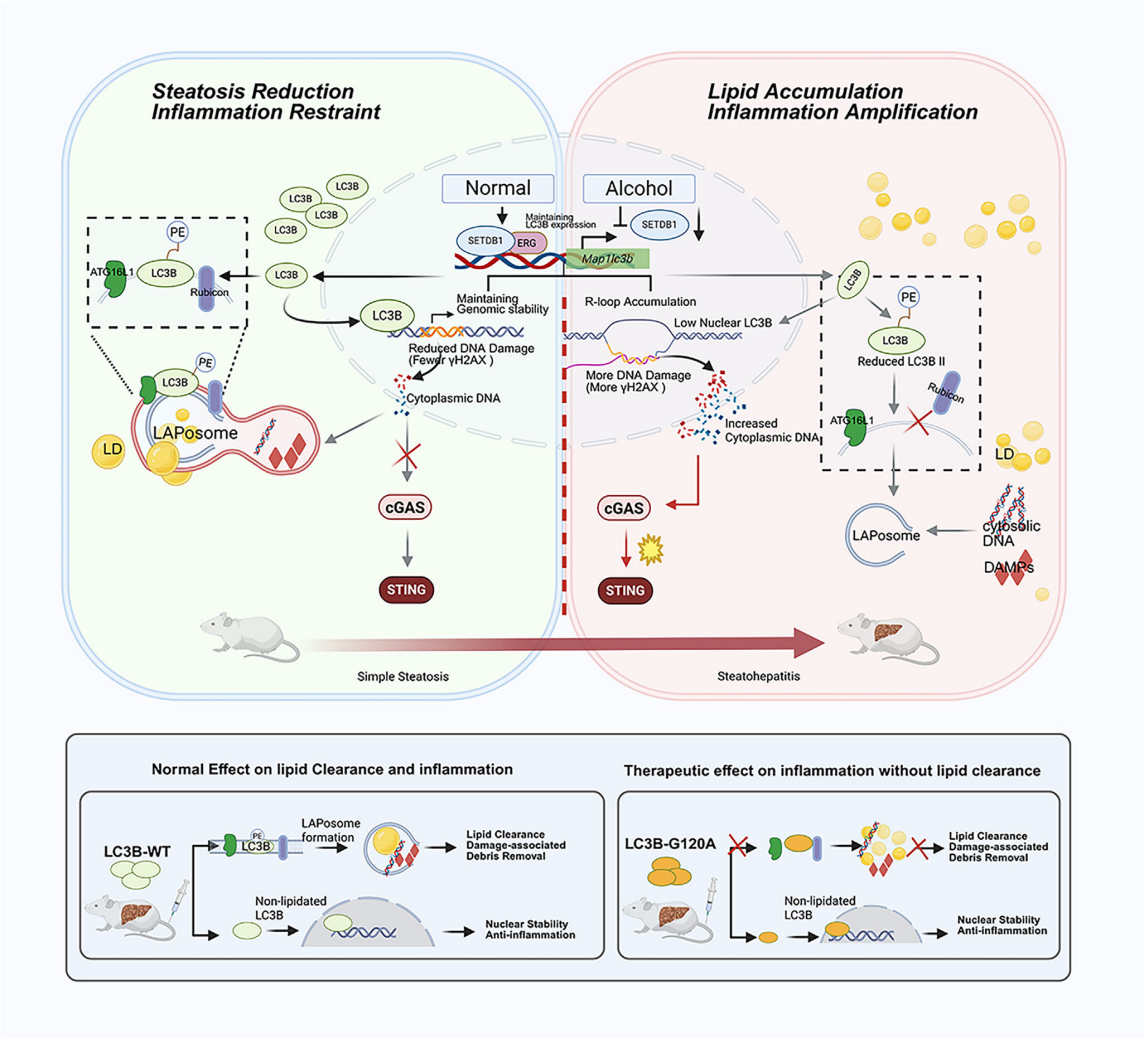
LC3B Mediates SETDB1 Deficiency‑Driven Alcoholic Steatohepatitis via Lipidation‑Dependent LAP and Lipidation‑Independent Nuclear Genomic Stabilization.

The present study identifies a protective SETDB1‐ERG‐LC3B axis in alcoholic liver disease progression. SETDB1 maintains Rubicon membrane localization to facilitate LAP, which mediates lipid droplet clearance and inflammatory suppression via the ATG16L1 WD40 domain. Concurrently, SETDB1 promotes nuclear translocation of non‐lipidated LC3B to preserve genomic stability and suppress cGAS‐STING‐driven inflammation. Thus, SETDB1 integrates epigenetic control, selective autophagy, and innate immunity as a critical molecular switch in the transition from steatosis to steatohepatitis (Figure 11).

We demonstrate that SETDB1 expression is progressively downregulated in livers of alcohol‐fed mice and ethanol‐treated hepatocytes, correlating with disease severity. Hepatocyte‐specific *Setdb1* knockout mice exhibit pronounced hepatic steatosis, inflammation, and fibrosis even under pair‐fed conditions, indicating that SETDB1 deficiency enhances hepatic susceptibility to nutritional stress. This phenotype suggests that loss of SETDB1 lowers the liver's tolerance threshold to basal metabolic stress, providing a molecular basis for the “multiple‐hit” hypothesis in ALD progression.

However, analysis of multiple human ALD datasets (GSE28619, GSE103580, GSE95246, GSE143318) did not reveal a consistent linear downregulation of SETDB1 expression across different disease stages. This discrepancy between animal models and human samples warrants careful consideration. Human ALD is inherently more complex and heterogeneous than controlled animal models. Unlike mice fed a standardized alcohol diet for a defined period (4–8 weeks in our study, representing early to mid‐stage ALD with steatosis and mild inflammation but no significant fibrosis), human patients exhibit wide variations in drinking patterns, duration, and quantity of alcohol consumption, as well as genetic backgrounds and environmental factors that may influence SETDB1 expression. Moreover, early‐stage liver tissue samples from ALD patients are extremely difficult to obtain, as patients typically present only after disease progression to advanced stages. Notably, while 8‐week alcohol‐fed wild‐type mice exhibited only mild fibrosis, SETDB1 deficiency dramatically exacerbated hepatic fibrosis and inflammation, suggesting that SETDB1 downregulation, even if transient, critically accelerates disease progression.

Hepatic SETDB1's expression in humans is apparently active in the embryonic and fetal stage and mild active in adult [40, 41, 42]. We speculate that SETDB1 tethered‐downregulation primarily occurs in early ALD stages, acting as a “first hit” [43, 44] that increases hepatic vulnerability and accelerates disease progression. Indeed, our data in *Setdb1* HKO mice clearly demonstrate that while 8‐week alcohol‐fed wild‐type mice exhibit only mild fibrosis, SETDB1 deficiency dramatically exacerbates hepatic fibrosis and inflammation, supporting its role as a critical modulator of disease susceptibility. This biphasic expression pattern is not unique to ALD, our previous work [17] has also demonstrated SETDB1 downregulation in non‐alcoholic fatty liver disease, suggesting that SETDB1 may serve as a common susceptibility factor in various liver pathologies rather than an ALD‐specific marker. Collectively, our findings position SETDB1 as a critical molecular switch in the transition from alcoholic steatosis to steatohepatitis and fibrosis, where its downregulation, even if transient, can profoundly accelerate disease progression.

Mechanistically, we found that SETDB1 interacts with the transcription factor ERG through its Tudor domain, functioning as a transcriptional cofactor to promote *Map1lc3b* transcription independent of its canonical H3K9me3 methyltransferase activity [35]. The *Map1lc3b* promoter lacks significant H3K9me3 enrichment, indicating this regulation operates through a non‐canonical mechanism involving recruitment of coactivators such as CBP/p300 to promote open chromatin conformation [45, 46]. SETDB1 deficiency leads to reduced LC3B expression, consequently impairing downstream LAP function. Importantly, AAV8‐mediated *Map1lc3b* overexpression in *Setdb1* HKO mice effectively ameliorates ALD pathology, validating the therapeutic potential of targeting this axis.

SETDB1 deficiency does not affect core canonical autophagy components but significantly alters Rubicon subcellular localization, redistributing it from membrane fractions to the cytosol. As a key positive regulator of LAP, Rubicon mislocalization directly impairs LAP function, manifested by reduced LC3B‐II/p62 ratio and defective lipid droplet clearance. Rubicon membrane recruitment is tightly regulated by its interaction with phosphatidylserine‐enriched domains on phagosomal membranes [24], and the WD40 domain of ATG16L1 plays an essential role in facilitating this process [47]. These findings establish that SETDB1 specifically regulates LAP rather than canonical autophagy through modulating Rubicon membrane localization.

SETDB1 deficiency reduces Rubicon membrane association without altering its total protein level, indicating a post‐transcriptional mechanism. Rubicon membrane recruitment depends on phosphatidylserine (PS)‐enriched domains and association with the VPS34 complex [24]. Given SETDB1's role as a methyltransferase and transcriptional cofactor, it may regulate the expression of PS‐metabolizing enzymes or unknown scaffold proteins that anchor Rubicon to membranes. Alternatively, SETDB1 may methylate non‐histone proteins such as VPS34, Beclin1, or Rubicon itself to modulate their lipid‐binding affinity, as reported for SETDB1‐mediated AKT methylation [48, 49, 50]. Future lipidomics, proximity labeling, and methyl‐proteomics studies are needed to dissect this link.

The differential phenotype of the lipidation‐deficient LC3B‐G120A mutant provides critical mechanistic insight: while failing to rescue SETDB1 deficiency‐induced steatosis, it partially suppresses inflammation [22, 51]. This reveals dual functions of LC3B—lipidation‐dependent cytoplasmic LAP mediating lipid droplet clearance through fusion with lysosomes [52, 53], and lipidation‐independent nuclear translocation where it reduces R‐loop accumulation and attenuates γH2AX foci formation, thereby preserving genomic stability. R‐loops are well‐established sources of DNA damage and genomic instability [54, 55]. Nuclear localization signal‐tagged LC3B effectively rescues nuclear instability, whereas nuclear export signal‐tagged LC3B fails to do so, confirming that nuclear residency is essential for this protective function [56, 57].

How does non‐lipidated LC3B regulate R‐loop homeostasis? Yoon et al. recently demonstrated that LC3B directly binds R‐loops via its RNA recognition motif (RRM), promoting transcription‐associated homologous recombination repair [58]. LC3B is recruited to DNA double‐strand breaks in transcriptionally active regions in an R‐loop‐dependent manner, facilitating BRCA1 recruitment and end‐resection. LC3B depletion causes R‐loop accumulation, reduced sister chromatid exchange, and chromosomal instability. Additionally, LC3B binds the 3'UTR of *BRCA1* mRNA via its RRM, enhancing *BRCA1* transcript stability. Beyond DNA repair, LC3B acts as an RNA‐binding protein triggering mRNA degradation [59] and can function as a nuclear transcriptional cofactor for LMX1B [39, 58, 60]. Collectively, these findings support a model in which non‐lipidated LC3B translocates to the nucleus under stress, binds R‐loops via its RRM, facilitates their resolution, and stabilizes DNA repair factors. Future work should identify the signals triggering LC3B nuclear translocation in ALD and its precise interactions with the R‐loop resolution machinery.

AAV8‐mediated *Map1lc3b* overexpression in *Setdb1* HKO mice significantly ameliorates alcohol‐induced liver injury, validating the therapeutic potential of targeting this axis. Given the early downregulation of SETDB1 in ALD, enhancing SETDB1 function or supplementing LC3B in early disease stages may prevent disease progression. However, the potential oncogenic role of SETDB1 in advanced stages [48] necessitates stage‐specific therapeutic strategies.

Future efforts should focus on developing stage‐specific strategies to stabilize SETDB1 or enhance LC3B function in early ALD, while elucidating the regulatory networks controlling Rubicon membrane localization and nuclear LC3B activity. Large‐scale clinical validation of SETDB1 and LC3B as biomarkers will be essential to guide precision interventions targeting the steatosis‐to‐steatohepatitis transition.

## Author Contributions

Yi Zhang and Tan Wei performed major experimental procedures, including reagent preparation and cellular assays, and Yi Zhang is additionally responsible for figure preparation and manuscript drafting. Jiahang Wu, and Chuixu Lin conducted critical cell‐based experiments and validations. Meiqi Song, Pengfei Gao, and Xu Wu carried out animal experiments and established disease models. Hongzhi Wang provided technical support and guidance for the immuno‐EM and cGAS‐STING signaling experiments. Shuting Shi, Shishun Huang, and Leiming Jiang performed statistical analysis and bioinformatics investigations. Lihui Qu provided funding acquisition and supervised animal studies. Zhigang Wang conceived the study hypothesis, designed the experimental framework, and provided overarching project supervision. All authors reviewed and approved the final version.

## Funding

This work was supported by the Guangdong Basic and Applied Basic Research Foundation (2023A1515012419).

## Conflicts of Interest

The authors declare no conflicts of interest.

## Supporting information




**Supporting File**: advs75668‐sup‐0001‐SuppMat.docx.

## Data Availability

The data that support the findings of this study are available from the corresponding author upon reasonable request.
