# Supplementary material for: LC3B Mediated SETDB1‐Accounted Alcoholic Steatohepatitis via Lipidation‐Dependent LAP and Lipidation‐Independent Nuclear Stabilization
Source: Adv Sci (Weinh). 2026 May 19:e13189. Online ahead of print. doi: 10.1002/advs.202513189 (PMC13335790; doi:10.1002/advs.202513189)
Supplement: Supplementary file 1 — Supporting File: advs75668‐sup‐0001‐SuppMat.docx. [file ADVS-9999-e13189-s001.docx]

**LC3B mediated SETDB1-accounted alcoholic steatohepatitis via lipidation-dependent LAP and lipidation-independent nuclear stabilization**


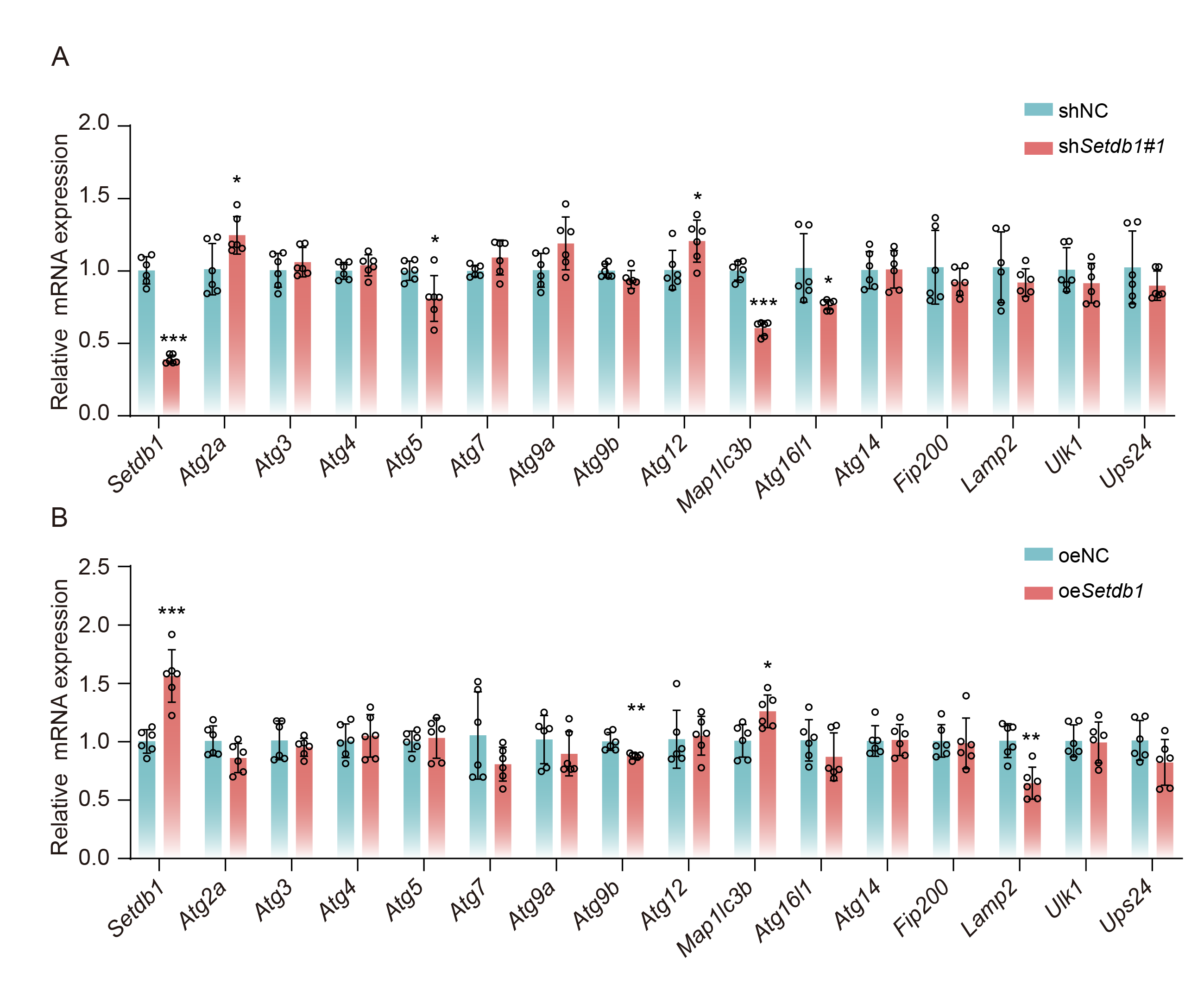


**Figure S1. Screening of autophagy-related genes regulated by SETDB1 in AML12 cells.**

(A) Relative mRNA expression of indicated autophagy-related genes in shNC and sh*Setdb1#1* AML12 cells, determined by qPCR (n=6 per group). (B) Relative mRNA expression of indicated autophagy-related genes in oeNC and oe*Setdb1* AML12 cells. For comparisons between two groups, Student's t-test or Welch's t-test was applied. **p* < 0.05, ***p* < 0.01, ****p* < 0.001.


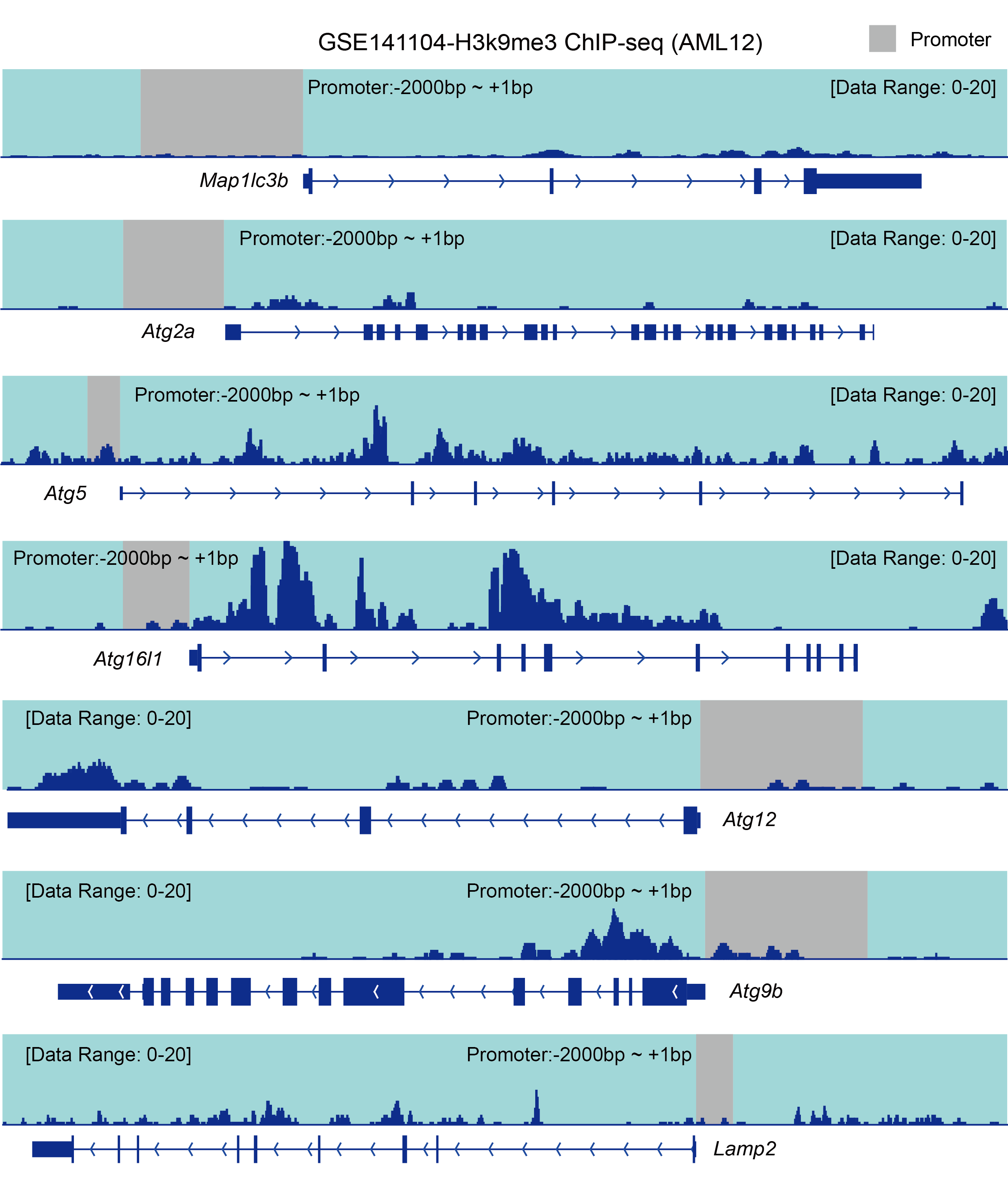


**Figure S2. H3K9me3 enrichment analysis at promoter regions of autophagy-related genes.**

H3K9me3 enrichment at *Setdb1*-regulated autophagy gene promoters in AML12 cells (GSM4195024, GSE141104).


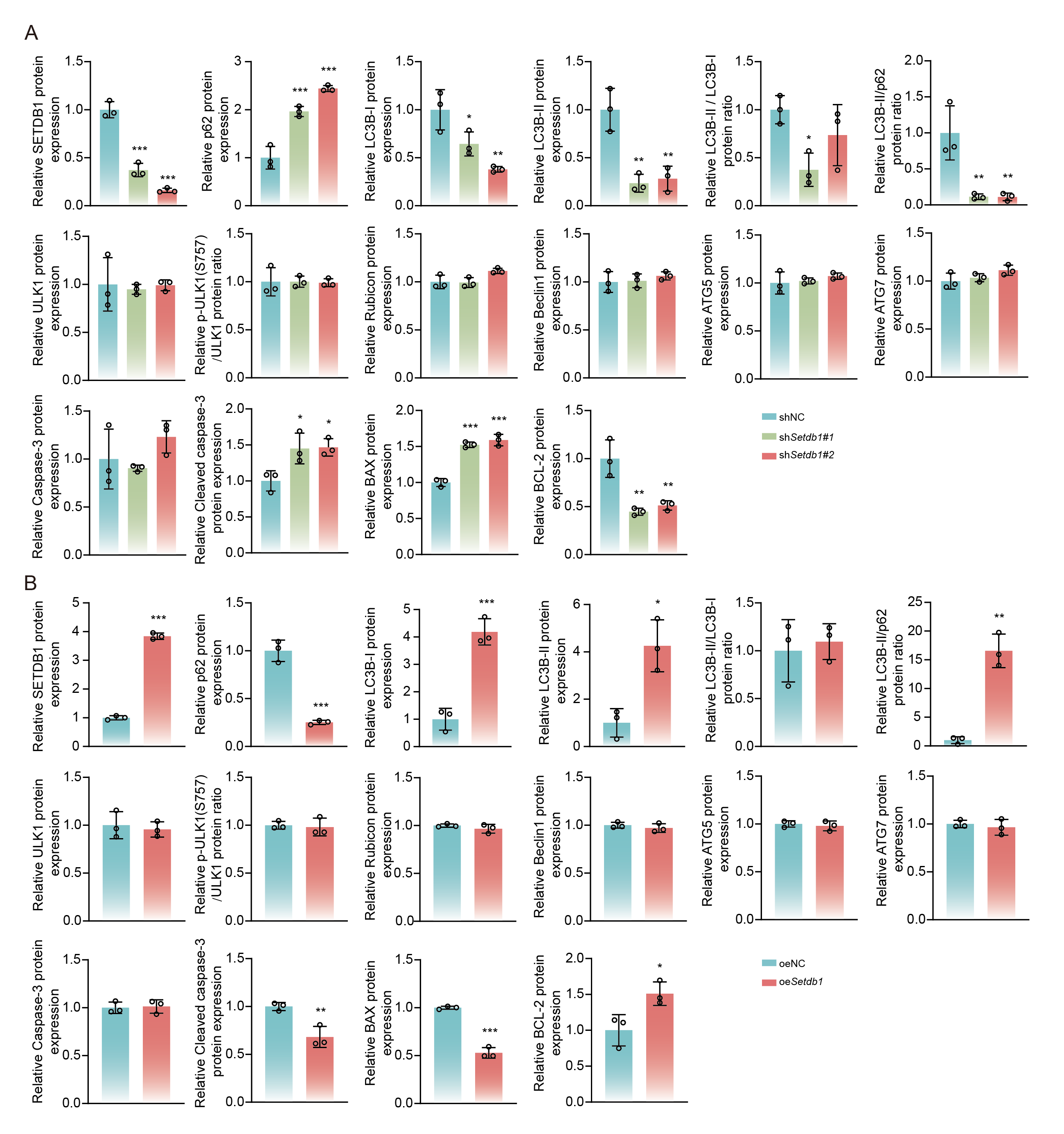


**Figure S3. Quantitative analysis of autophagy and apoptosis-related proteins in SETDB1-manipulated AML12 cells.**

(A) Quantitative analysis of indicated protein levels in shNC, sh*Setdb1#1*, and sh*Setdb1#2* AML12 cells , corresponding to Figure 4D. Data include SETDB1, p62, LC3B-I, LC3B-II, LC3B-II/LC3B-I ratio, LC3B-II/p62 ratio, ULK1, p-ULK (S757), Rubicon, Beclin1, ATG5, ATG7, caspase-3, cleaved caspase-3, BAX, and BCL-2 (n=3 per group). (B) Quantitative analysis of the same protein panels in oeNC and oe*Setdb1* AML12 cells (n=3 per group). For comparisons between two groups, Student's t-test (B) was used. For multiple group comparisons, one-way ANOVA followed by Bonferroni's post-hoc test (A) was applied. **p* < 0.05, ***p* < 0.01, ****p* < 0.001; ns, not significant.


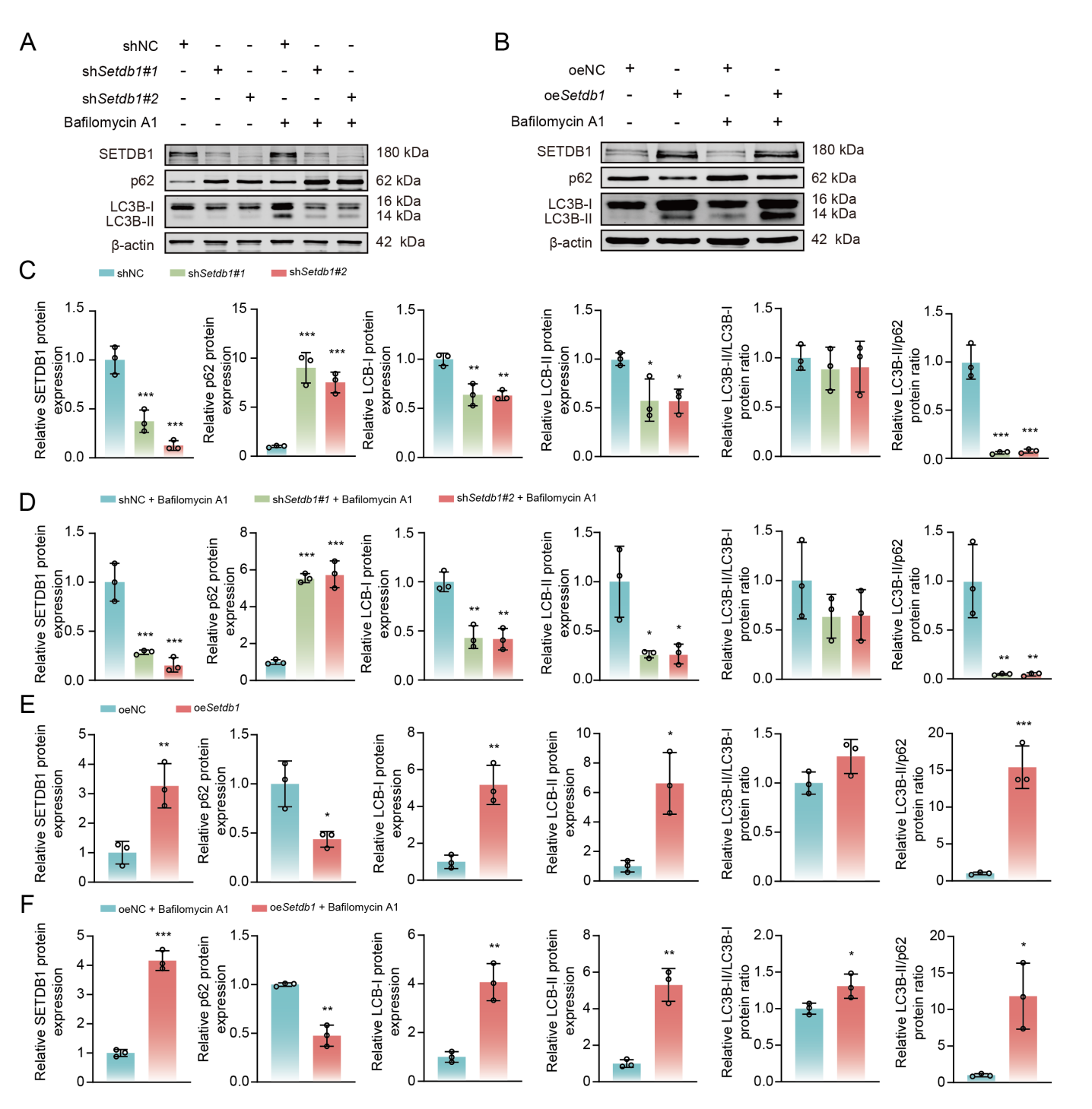


**Figure S4. SETDB1 regulates LC3B lipidation and p62 turnover in a lysosome-dependent manner.**

(A) Representative Western blot images of SETDB1, p62, LC3B-I, and LC3B-II in shNC and sh*Setdb1* AML12 cells treated with or without BAF1 (100 nM, 6 h). β-actin served as loading control. (B) Representative Western blot images of the same proteins in oeNC and oe*Setdb1* AML12 cells with or without BAF1 treatment. (C) Quantitative analysis of SETDB1, p62, LC3B-I, LC3B-II, LC3B-II/LC3B-I ratio, and LC3B-II/p62 ratio in shNC and sh*Setdb1* cells without BAF1 treatment (n=3 per group). (D) Quantitative analysis of the same parameters in shNC and sh*Setdb1* cells with BAF1 treatment. (E-F) Quantitative analysis of the same parameters in oeNC and oe*Setdb1* cells without (E) or with (F) BAF1 treatment (n=3 per group). For comparisons between two groups, Student's t-test (E, F) was used. For multiple group comparisons, one-way ANOVA followed by Bonferroni's post-hoc test (C, D) was applied. **p* < 0.05, ***p* < 0.01, ****p* < 0.001; ns, not significant.


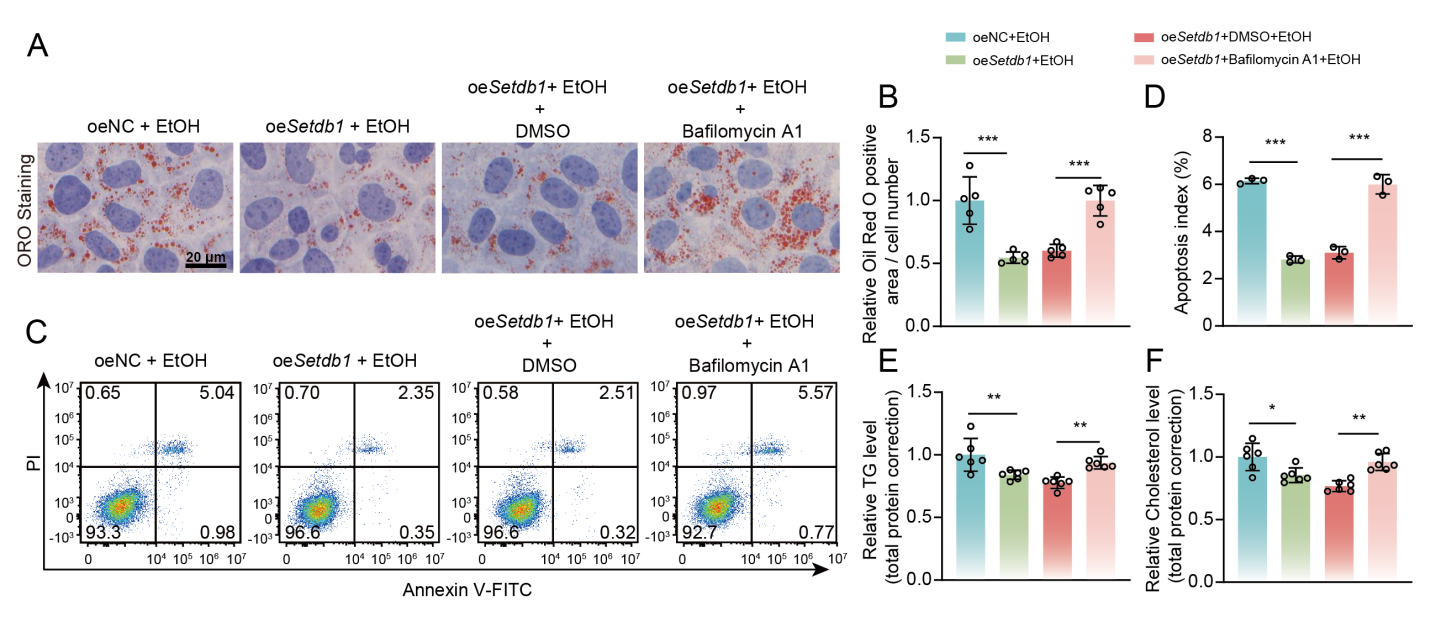


**Figure S5. SETDB1 protects against ethanol-induced lipotoxicity in a lysosome-dependent manner.**

(A) Representative images of Oil Red O staining in AML12 cells from the indicated groups: oeNC + 200 mM EtOH, oe*Setdb1* + 200 mM EtOH, oe*Setdb1* + 200 mM EtOH + DMSO, and oe*Setdb1* + 200 mM EtOH + BAF1. Scale bar, 20 μm. (B) Quantification of Oil Red O-positive areas (n=5 per group). (C) Representative flow cytometry plots of apoptosis in the indicated groups. (D) Quantification of apoptotic cell percentage (n=3 per group). (E-F) Quantification of cellular TG and TC content in the indicated groups (n=6 per group). All data are presented as mean ± SD. For multiple group comparisons, one-way ANOVA followed by Tukey's post-hoc test (B, D, E, F) was applied. **p* < 0.05, ***p* < 0.01, ****p* < 0.001; ns, not significant.

**
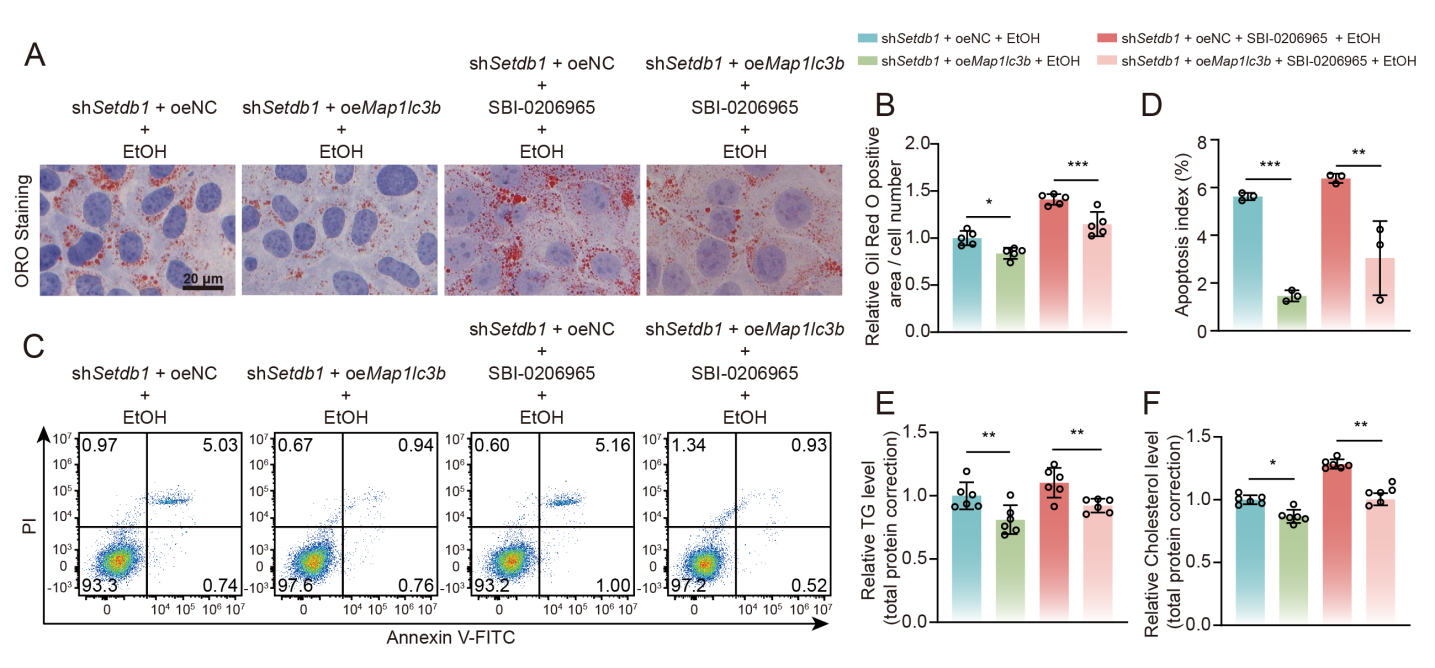
Figure S6. LC3B-mediated rescue of SETDB1 deficiency-induced lipotoxicity is independent of ULK1.**

(A) Representative images of Oil Red O staining in the indicated groups: sh*Setdb1* + oeNC, sh*Setdb1* + oe*Map1lc3b*, sh*Setdb1* + oeNC + SBI-0206965, and sh*Setdb1* + oe*Map1lc3b* + SBI-0206965, all treated with 200 mM ethanol. Scale bar, 20 μm. (B) Quantification of Oil Red O-positive areas (n=5 per group). (C) Representative flow cytometry plots of apoptosis. (D) Quantification of apoptotic cell percentage (n=3 per group). (E-F) Quantification of cellular TG and TC content (n=6 per group). All data are presented as mean ± SD. For multiple group comparisons, one-way ANOVA followed by Tukey's post-hoc test (B, D, E, F) was applied. **p* < 0.05, ***p* < 0.01, ****p* < 0.001; ns, not significant.


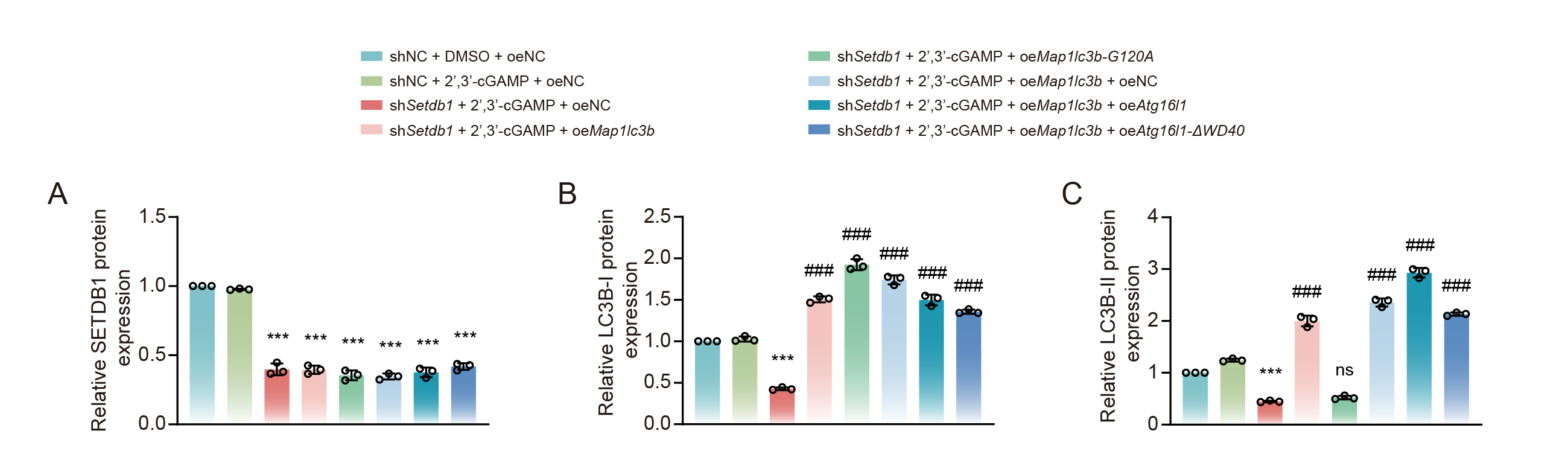


**Figure S7. Validation of SETDB1 and LC3B manipulation in cGAS-STING experiments.**

(A) Quantitative analysis of SETDB1 protein levels in the eight experimental groups from Figure 6H. Statistical comparisons: *p < 0.05 versus group 2 (shNC + 2'3'-cGAMP + oeNC), confirming successful SETDB1 knockdown in groups 3-8 (n=3 per group). (B) Quantitative analysis of LC3B-I protein levels. *p < 0.05 for group 3 versus group 2, indicating reduced LC3B-I upon SETDB1 knockdown; #p < 0.05 for groups 4-8 versus group 2, confirming successful LC3B overexpression in these groups (n=3 per group). (C) Quantitative analysis of LC3B-II protein levels. *p < 0.05 for group 3 versus group 2, indicating reduced LC3B-II upon SETDB1 knockdown; #*p* < 0.05 for groups 4-8 versus group 2, confirming successful LC3B-II production in LC3B-overexpressing groups, with the exception of group 5 (LC3B-G120A), which showed no detectable LC3B-II, confirming its lipidation deficiency (n=3 per group). All data are presented as mean ± SD from three independent experiments. Statistical significance was determined by one-way ANOVA followed by Tukey's post-hoc test.


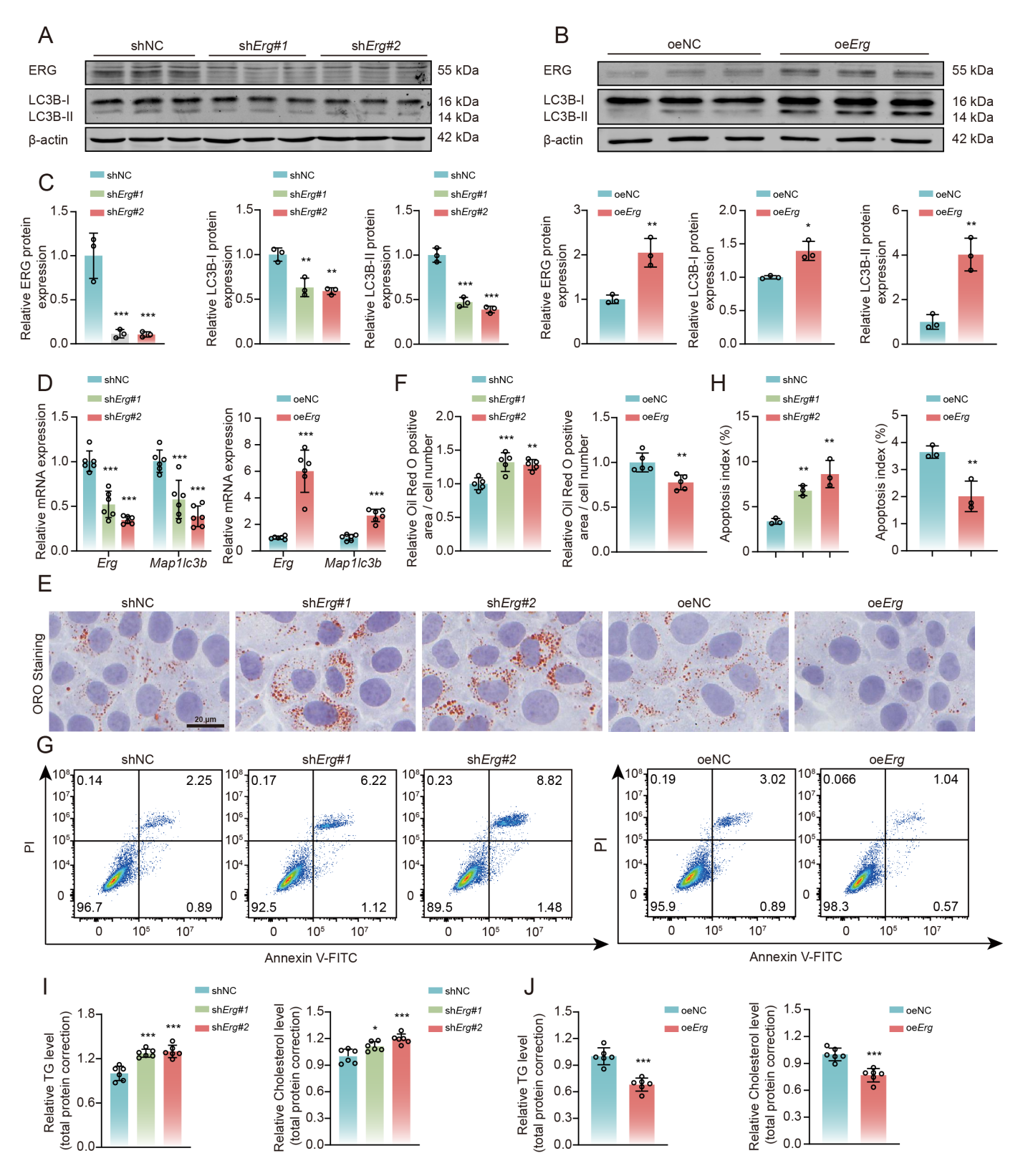


**Figure S8. ERG regulates Map1lc3b expression and modulates ethanol-induced lipotoxicity in AML12 cells.**

(A) Representative Western blot images of ERG, LC3B-I, and LC3B-II in shNC and sh*ERG* AML12 cells. β-actin served as loading control. (B) Representative Western blot images of ERG, LC3B-I, and LC3B-II in oeNC and oe*ERG* AML12 cells. (C) Quantitative analysis of ERG, LC3B-I, and LC3B-II protein levels in sh*ERG* and oe*ERG* cells (n=3 per group). (D) Relative mRNA expression of *ERG* and *Map1lc3b* in sh*ERG* and oe*ERG* cells, determined by qPCR (n=6 per group). (E) Representative images of Oil Red O staining in sh*ERG* and oe*ERG* cells treated with 200 mM ethanol. Scale bar, 20 μm. (F) Quantification of Oil Red O-positive areas (n=5 per group). (G) Representative flow cytometry plots of apoptosis in the indicated groups. (H) Quantification of apoptotic cell percentage (n=3 per group). (I) Quantification of cellular TG and TC content in sh*ERG* and shNC cells (n=6 per group). (J) Quantification of cellular TG and TC content in oe*ERG* and oeNC cells (n=6 per group). All data are presented as mean ± SD. For comparisons between two groups, Student's t-test (C, F, J, H, D) or Welch's t-test (C, D) was used. For multiple group comparisons, one-way ANOVA followed by Bonferroni's post-hoc test (C, F, I, H, D) was applied. **p* < 0.05, ***p* < 0.01, ****p* < 0.001; ns, not significant.


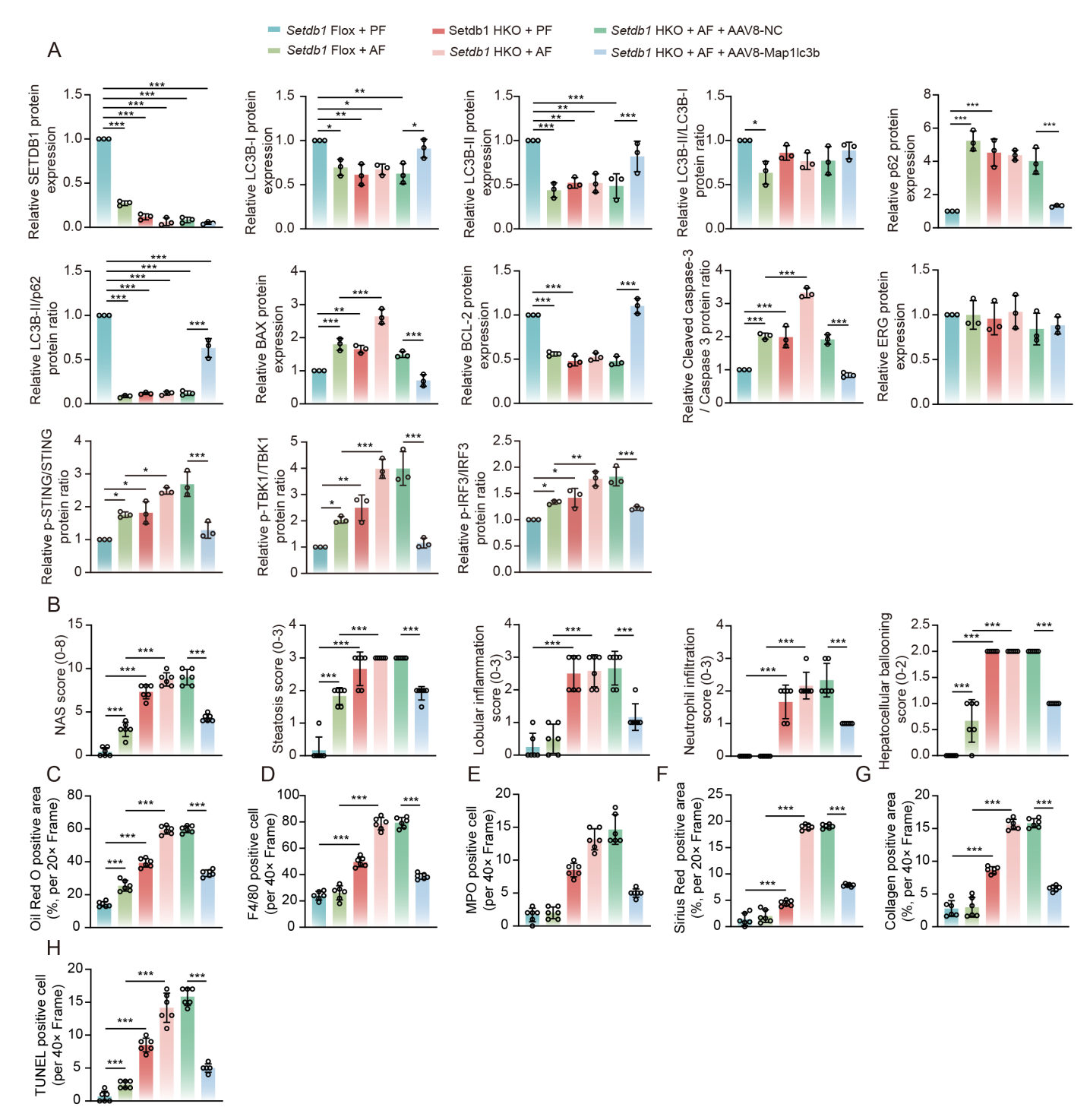


**Figure S9. Quantitative analysis of liver pathology in Setdb1 HKO mice and validation of LC3B-mediated rescue.**

(A) Quantitative analysis of Western blot data from Figure 8B, including SETDB1, LC3B-I, LC3B-II, LC3B-II/LC3B-I ratio, LC3B-II/p62 ratio, BAX, BCL-2, cleaved caspase-3/caspase-3 ratio, p-STING/STING ratio, p-TBK1/TBK1 ratio, and p-IRF3/IRF3 ratio (n=3 per group). (B) Histological scoring including NAS score, steatosis score, lobular inflammation score, and neutrophil infiltration score (n=6 per group). (C-H) Quantification of Oil Red O-positive areas (C), F4/80-positive cells (D), MPO-positive cells (E), Sirius Red-positive areas (F), collagen-positive areas (G), and TUNEL-positive cells (H) (n=6 per group). All data are presented as mean ± SD. For multiple group comparisons, one-way ANOVA followed by Tukey's post-hoc test (A, B, C, D, E, F), Tamhane's T2 post-hoc test (G, H) was applied. **p* < 0.05, ***p* < 0.01, ****p* < 0.001; ns, not significant.

| **Table S1**. Sequence information shRNA | | |
| --- | --- | --- |
| **target Gene** | **product name** | **target sequence** |
| *Setdb1* | sh*Setdb1#1* | CCGG-GCCTTGATCTTCCATGTCATT-CTCGAG-AATGACATGGAAGATCAAGGC-TTTTTT |
| *Setdb1* | sh*Setdb1#2* | CCGG-TGCTATCTGGGAACCATATTG-CTCGAG-CAATATGGTTCCCAGATAGCA-TTTTTT |
| *Erg* | sh*Erg#1* | GATCG-GCCGACATTCTTCTCTCACATCTCGAGATGTGAGAGAAGAATGTCGGC-TTTTTT |
| *Erg* | sh*Erg#2* | GATCG-CCGATGACGTTGATAAGGCTTCTCGAGAAGCCTTATCAACGTCATCGG-TTTTTT |
| *Rubicon* | sh*Rubicon#1* | GATCG-CAGTGTCGCAAGCCGTCAAATCTCGAGATTTGACGGCTTGCGACACTG-TTTTTT |
| *Rubicon* | sh*Rubicon#2* | GATCG-GGACAAAGAAGAGCCATATTCCTCGAGGAATATGGCTCTTCTTTGTCC-TTTTTT |
| *Atg3* | sh*Atg3#1* | GATCG-CATATCACAACACAGGTATTACTCGAGTAATACCTGTGTTGTGATATG-TTTTTT |
| *Atg3* | sh*Atg3#2* | GATCG-GTACATCACTTACGACAAATACTCGAGTATTTGTCGTAAGTGATGTAC-TTTTTT |

| **Table S2**. Primer sequence | |
| --- | --- |
| **primer name** | **primer sequence** |
| *18S* | F:5' GACCTTCAAGCAGCGCCGGA 3' |
|  | R:5' TCAGGGGCCTGTGGTTTCCC 3' |
| *Setdb1* | F:5' AAGGTGGATTTGCTGACTCTGA 3' |
|  | R:5' CTGGCATCTTGTTTCGGTTCT 3' |
| HKO mouse flox | F:5' TCTGTACCTTGCTCAGACTGCCAT 3' |
|  | R:5' CTTCCAGGACCTAATAAGTTCAGTT 3' |
| Cre-amplicon | F:5' GAAGCAGAAGCTTAGGAAGATGG 3' |
|  | R:5' TTGGCCCCTTACCATAACTG 3' |
| HKO mouse *Setdb1* | F:5' GACTTCATCAGGCACATATGAAT 3' |
|  | R:5' GGTTTGTGAGTTTGAGAGCAAT 3' |
| *Map1lc3b* | F:5' GTCCTGGACAAGACCAAGTTCC 3' |
|  | R:5' CCATTCACCAGGAGGAAGAAGG 3' |
| *Map1lc3b*-segment1 | F:5' CCACCACGCTCAGCTTTTTTAA 3' |
|  | R:5' TGAGGACCTGGATTTGGTTCTC 3' |
| *Map1lc3b*-segment2 | F:5' CTTTTAGCCTGGTCTCTTGCACC 3' |
|  | R:5' TGCTGTTTGTCCAAGGGATCTT 3' |
| *Map1lc3b*-segment3 | F:5' GCCCTCAGGATTTCACTCCATA 3' |
|  | R:5' TAGAGTGTGGAGCAGCCACC 3' |
| *Map1lc3b*-segment4 | F:5' GGTGGCTGCTCCACACTCTA 3' |
|  | R:5' GGGTGGCTTTATCACAGGCAT 3' |
| *Map1lc3b*-segment5 | F:5' CAGTCTGATTGCTCACCAACC 3' |
|  | R:5'CCTGTCCACTCTCTCTTCCCA 3' |
| *A-Fabp* | F:5'CCGCAGACGACAGGA 3' |
|  | R:5'CTCATGCCCTTTCATAAACT 3' |
| *F4/80* | F:5' CTTTGGCTATGGGCTTCCAGTC3' |
|  | R:5'GCAAGGAGGACAGAGTTTATCGTG 3' |
| *Cd68* | F:5' CGGATTTGAATTTGGGCTTG 3' |
|  | R:5' GGTAGACAACAGCCGCATC 3' |
| *Tnfa* | F:5' CCCTCACACTCAGATCATCTTCT 3' |
|  | R:5' GCTACGACGTGGGCTACAG 3' |
| *Mcp-1* | F:5' CCACTCACCTGCTGCTACTCA 3' |
|  | R:5' TGGTGATCCTCTTGTAGCTCTCC 3' |
| *l1-1β* | F:5' CTGGTGTGTGACGTTCCCATTA 3' |
|  | R:5' CCGACAGCACGAGGCTT 3' |
| *Ifnb1* | F:5' CCACCACAGCCCTCTCCATCAACTAT 3' |
|  | R:5' CAAGTGGAGAGCAGTTGAGGACATC 3' |
| *Isg15* | F:5' ACGGTCTTACCCTTTCCAGTC 3' |
|  | R:5' CCCCTTTCGTTCCTCACCAG 3' |
| *Cxcl10* | F:5' CCAAGTGCTGCCGTCATTTTC 3' |
|  | R:5' GGCTCGCAGGGATGATTTCAA 3' |
| *Ccl5* | F:5' TGAAGATCTCTGCAGCTGCCC 3' |
|  | R:5' GATTGGAGCACTTGCTGCTGG 3' |
| *Irf7* | F:5' CCTCTTGCTTCAGGTTCTGC 3' |
|  | R:5' GCTGCATAGGGTTCCTCGTA 3' |
| *Rsad2* | F:5' TGGGGATGCTGGTGCCCACT 3' |
|  | R:5' ACCCCGGACCTGTGGCTGTT 3' |
| *Cpt1a* | F:5' GGTCTTCTCGGGTCGAAAGC 3' |
|  | R:5' TCCTCCCACCAGTCACTCAC 3' |
| *Acox1* | F:5' AACCAGAAGGGAATGGGTGTCAAG 3' |
|  | R:5' CCCTTTCGCTCCAATTTGTTTGGG 3' |
| *Acadm* | F:5' AGGGTTTAGTTTTGAGTTGACGG 3' |
|  | R:5' CCCCGCTTTTGTCATATTCCG 3' |
| *Fatp* | F:5' CCGGTGTGGTGGCTGCTCTTCTC 3' |
|  | R:5' GCTGCCATCTCCCCGCCATAAATG 3' |
| *Fabp1* | F:5' TCACCATCACCTATGGACCCA 3' |
|  | R:5' TCCAGTTCGCACTCCTCCC 3' |
| *Cyp4a* | F:5' TCCAGGTTTGCACCAGACTCT 3' |
|  | R:5' TCCTGGCTCCTCCTGAGAAG 3' |
| *ACTB* (for DRIP-qPCR ) | F:5' CGGGGTCTTTGTCTGAGC 3' |
| *ACTB* | R:5' CAGTTAGCGCCCAAAGGAC 3' |
| *POLR3A* (for DRIP-qPCR ) | F:5' TCTCTTGGCCTTGGGCTTTC 3' |
| *POLR3A* | R:5' AGGAGCAGCAGTTAGAATGGG 3' |
| *RBM19* (for DRIP-qPCR ) | F:5' TCCCCGGATAAGTGTCATCCT 3' |
| *RBM19* | R:5' AGCGCATGTTGATCGGGAAA 3' |

| **Table S3.** Antibodies information | | |
| --- | --- | --- |
| Antibodies | source | Identifier |
| Monoclonal anti-β actin antibody（mouse） | Sigma-Aldrich | Cat#A6316; lot#000110618 |
| Beta Actin Monoclonal antibody | Proteintech Group | Cat#66009-1-Ig; lot#10074508 |
| Beta Actin Recombinant antibody | Proteintech Group | Cat#81115-1-RR; lot#23016582 |
| IRDye 800CW Goat anti-Rabbit | LI-Cor | Cat#926-32211 |
| IRDye 680RD Goat anti-Mouse | LI-Cor | Cat#926-68070 |
| HRP-conjugated IgG Fraction Monoclonal Mouse Anti-Rabbit IgG, Light Chain Specific | Proteintech Group | Cat#SA00001-7L; lot#20001251 |
| SETDB1 Polyclonal antibody | Proteintech Group | Cat#11231-1-AP; lot#00174845; |
| Anti-LC3B antibody | Abcam | Cat#ab48394; lot#1005940-1 |
| Anti-ERG Antibody | Abcam | Cat#ab92513; lot#1003022-1 |
| Phospho-TMEM173/STING(Ser366) Antibody | Abmart | Cat#TA7416 |
| Phospho-NAK/TBK1(S172) Antibody | Abmart | Cat#T58364 |
| Phospho-IRF3(S396) Antibody | Abmart | Cat#TU22772 |
| Anti-IRF3 Rabbit pAb | Servicebio | Cat#GB11368-50;  lot#AC260115010 |
| Anti-alpha Tubulin antibody[EPR13478(B)]-Loading Control | Abcam | Cat#ab176560;  lot#GR177622-71 |
| LC3B(E5Q2K) Mouse mAb | Cell Signaling Technology | Cat#83506;  lot#4 |
| Phospho-Histone H2A.X(Ser139)(20E3) Rabbit mAb | Cell Signaling Technology | Cat#9718;  lot#25 |
| ULK1 Monoclonal antibody | Proteintech Group | Cat#68445-1-Ig;  lot#10014818 |
| Rubicon Polyclonal antibody | Proteintech Group | Cat#21444-1-AP;  lot#00147893 |
| TMEM173/STING Monoclonal antibody | Proteintech Group | Cat#66680-1-Ig;  lot#10015913 |
| Bcl2 Polyclonal antibody | Proteintech Group | Cat#26593-1-AP;  lot#00168658 |
| BAX Polyclonal antibody | Proteintech Group | Cat#50599-2-Ig;  lot#00184181 |
| ATP1A1 Polyclonal antibody | Proteintech Group | Cat#14418-1-AP;  lot#00181368 |
| DYKDDDDK tag Monoclonal antibody | Proteintech Group | Cat#66008-4-Ig;  lot#10072487 |
| Anti-APG5L/ATG5 Rabbit pAb | Servicebio | Cat#GB115761-50;  lot#AC251118241 |
| Anti-ATG7 Rabbit pAb | Servicebio | Cat#GB11399-50;  lot#AC251118050 |
| Anti-ULK1(Phospho S757) antibody | Abcam | Cat#ab229909;  lot#1094678-21 |
| Anti-Caspase-3 antibody | Abcam | Cat#ab13585;  lot#1062802-3 |
| Anti-Cleaved Caspase-3 antibody | Abcam | Cat#ab214430;  lot#1002427-97 |
| Anti-LAMP2A antibody | Abcam | Cat#ab18528;  lot#1081330-19 |
| TBK1 Rabbit RecAb | Proteintech Group | Cat#83686-3-RR;  lot#23009415 |
| Anti-SQSTM1/p62 antibody | Abcam | Cat#ab109012;  lot#GR3241806-18 |
| Anti-Histone H3 antibody | Abcam | Cat#ab176916;  lot#GR3218257-6 |
| DNA-RNA hybried mAb (S9.6) | Active Motif | Cat#65983;  lot#25058174-12 |
| Perilipin-2 Rabbit PolyAb | Proteintech Group | Cat#15294-1-AP;  lot#00163610 |
| Beclin1 Mouse McAb | Proteintech Group | Cat#66665-1-Ig;  lot#10008909 |
| Anti-F4/80 Rabbit pAb | Servicebio | Cat#GB113373 |
| Recombinant Anti-Myeloperoxidase antibody (Rabbit mAb) | Servicebio | Cat#GB150006 |
| Anti-Collagen I Rabbit pAb | Servicebio | Cat#GB11022 |
| Cy3-labeled Goat Anti-Rabbit IgG (H+L) | Beyotime Biotechnology | Cat#A0516 |
| AF488-labeled Goat Anti-Mouse IgG (H+L) | Beyotime Biotechnology | Cat#A0428 |

| **Table S4**. Prediction of ERG binding sites in the *Map1lc3b* promoter | | | | | | | |
| --- | --- | --- | --- | --- | --- | --- | --- |
| Matrix ID | Name | Score | Relative score | Start | End | Strand | Predicted Sequence |
| MA0474.3 | ERG | 11.27 | 0.87 | 1041 | 1054 | + | AGATAGGAAGTAGC |
| MA0474.1 | ERG | 10.54 | 0.91 | 887 | 897 | + | GGAGGAAGTCT |
| MA0474.3 | ERG | 10.09 | 0.85 | 885 | 898 | + | AAGGAGGAAGTCTG |
| MA0474.3 | ERG | 9.23 | 0.84 | 1384 | 1397 | + | AGTAAGGAAATGAG |
| MA0474.1 | ERG | 9.04 | 0.89 | 1043 | 1053 | + | ATAGGAAGTAG |
| MA0474.1 | ERG | 8.44 | 0.88 | 1386 | 1396 | + | TAAGGAAATGA |
| MA0474.3 | ERG | 8.21 | 0.82 | 245 | 258 | + | GAAGAGGATGTTAG |
| MA0474.1 | ERG | 7.37 | 0.87 | 766 | 776 | + | AAAGGAAAGTA |
| MA0474.1 | ERG | 6.59 | 0.86 | 247 | 257 | + | AGAGGATGTTA |

| **Table S5**. Recombinant sequence | |
| --- | --- |
| **recombinant name** | **target sequence** |
| pCMV3-MAP1LC3B-His | ATGCCGTCCGAGAAGACCTTCAAGCAGCGCCGGAGCTTTGAACAAAGAGTGGAAGATGTCCGGCTCATCCGGGAGCAGCACCCCACCAAGATCCCAGTGATTATAGAGCGATACAAGGGGGAGAAGCAGCTGCCCGTCCTGGACAAGACCAAGTTCCTGGTGCCTGACCACGTGAACATGAGCGAGCTCATCAAGATAATCAGACGGCGCTTGCAGCTCAATGCTAACCAAGCCTTCTTCCTCCTGGTGAATGGGCACAGCATGGTGAGTGTGTCCACTCCCATCTCCGAAGTGTACGAGAGTGAGAGAGATGAAGACGGCTTCCTGTACATGGTTTATGCCTCGCAGGAGACATTCGGGACAGCAATGGCTGTGGGGGGTGGAGGCTCTCACCATCACCACCATCATCACCACCATCACTAA |
| MG52515-CH-His-Map1lc3b-G120A | GGTACC-ATGCCGTCCGAGAAGACCTTCAAGCAGCGCCGGAGCTTTGAACAAAGAGTGGAAGATGTCCGGCTCATCCGGGAGCAGCACCCCACCAAGATCCCAGTGATTATAGAGCGATACAAGGGGGAGAAGCAGCTGCCCGTCCTGGACAAGACCAAGTTCCTGGTGCCTGACCACGTGAACATGAGCGAGCTCATCAAGATAATCAGACGGCGCTTGCAGCTCAATGCTAACCAAGCCTTCTTCCTCCTGGTGAATGGGCACAGCATGGTGAGTGTGTCCACTCCCATCTCCGAAGTGTACGAGAGTGAGAGAGATGAAGACGGCTTCCTGTACATGGTTTATGCCTCGCAGGAGACATTCGCGACAGCAATGGCTGTGGGGGGTGGAGGCTCTCACCATCACCACCATCATCACCACCATCACTAAACTCGAG-TCTAGA |
| MG52515-CH-Flag-NES-Map1lc3b | GGTACC-ATGCTGCAGCTGCCACCGCTGGAGCGCCTGACGCTGGGCGGCAGCGGCGGCAGCGGCGGCAGCGACTACAAGGACGACGATGACAAGGGCGGCAGCGGCGGCAGCGGCGGCAGCCCGTCCGAGAAGACCTTCAAGCAGCGCCGGAGCTTTGAACAAAGAGTGGAAGATGTCCGGCTCATCCGGGAGCAGCACCCCACCAAGATCCCAGTGATTATAGAGCGATACAAGGGGGAGAAGCAGCTGCCCGTCCTGGACAAGACCAAGTTCCTGGTGCCTGACCACGTGAACATGAGCGAGCTCATCAAGATAATCAGACGGCGCTTGCAGCTCAATGCTAACCAAGCCTTCTTCCTCCTGGTGAATGGGCACAGCATGGTGAGTGTGTCCACTCCCATCTCCGAAGTGTACGAGAGTGAGAGAGATGAAGACGGCTTCCTGTACATGGTTTATGCCTCGCAGGAGACATTCGGGACAGCAATGGCTGTGGGGGGTGGAGGCTCTCACCATCACCACCATCATCACCACCATCACTAAACTCGAG-TCTAGA |
| MG52515-CH-Flag-mus-NLS-Map1lc3b | GGTACC-ATGCCCAAGAAGAAGCGCAAGGTGGGCGGCAGCGGCGGCAGCGGCGGCAGCGACTACAAGGACGACGATGACAAGGGCGGCAGCGGCGGCAGCGGCGGCAGCCCGTCCGAGAAGACCTTCAAGCAGCGCCGGAGCTTTGAACAAAGAGTGGAAGATGTCCGGCTCATCCGGGAGCAGCACCCCACCAAGATCCCAGTGATTATAGAGCGATACAAGGGGGAGAAGCAGCTGCCCGTCCTGGACAAGACCAAGTTCCTGGTGCCTGACCACGTGAACATGAGCGAGCTCATCAAGATAATCAGACGGCGCTTGCAGCTCAATGCTAACCAAGCCTTCTTCCTCCTGGTGAATGGGCACAGCATGGTGAGTGTGTCCACTCCCATCTCCGAAGTGTACGAGAGTGAGAGAGATGAAGACGGCTTCCTGTACATGGTTTATGCCTCGCAGGAGACATTCGGGACAGCAATGGCTGTGGGGGGTGGAGGCTCTCACCATCACCACCATCATCACCACCATCACTAAACTCGAG-TCTAGA |
| pcDNA3.1-Atg16l1 | GCTAGC-ATGGATTACAAGGATGACGACGATAAGGGTGGAGGTGGATCTTCGTCGGGCCTGCGCGCCGCAGACTTTCCCCGCTGGAAGCGTCACATCGCGGAGGAACTGAGGCGCCGGGACCGACTGCAGAGGCAGGCGTTCGAGGAGATCATTCTGCAGTATACCAAGTTGCTGGAAAAGTCAGATCTTCATTCAGTATTGACCCAGAAACTACAAGCAGAAAAGCATGACATGCCAAATAGGCATGAAATAAGTCCTGGACATGATGGTGCGTGGAATGATAGTCAACTACAAGAAATGGCCCAGTTGAGGATCAAACACCAGGAAGAGCTGACCGAACTGCACAAGAAGCGTGGGGAGTTAGCTCAGTTGGTGATTGACCTGAACAACCAAATGCAGCAGAAGGACAAGGAGATACAGATGAATGAAGCAAAGATTTCGGAGTATTTACAGACCATCTCTGACCTGGAGACAAACTGCCTGGACCTGCGCACCAAACTGCAGGACCTCGAGGTAGCCAACCAGACCCTGAAGGATGAGTATGACGCCCTGCAGATTACTTTTACTGCCCTAGAAGAGAAACTGAGGAAAACTACTGAGGAGAACCAGGAACTGGTCACCAGATGGATGGCTGAGAAGGCCCAAGAAGCCAATCGCCTCAATGCAGAGAATGAGAAGGACTCCAGGAGGCGTCAAGCACGGCTGCAGAAGGAGCTTGCAGAAGCAGCAAAGGAACCTCTACCTGTTGAACAGGATGATGACATTGAAGTCATTGTGGATGAGACCTCAGACCACACAGAAGAGACCTCTCCCGTCCGAGCTGTCAGCAGAGCAGCTACTAAGCGACTCTCGCAGCCTGCTGGAGGCCTTCTGGATTCTATCACTAATATCTTTGGGAGACGCTCTGTCTCTTCCATCCCAGTCCCCCAGGATATCATGGACACTCATCCTGCTTCTGGTAAAGATGTGAGAGTCCCAACTACTGCCTCGTATGTCTTCGATGCGCATGACGGAGAGGTCAACGCAGTGCAGTTCAGTCCAGGCTCCCGGTTGCTGGCCACTGGAGGCATGGACCGCAGGGTGAAACTTTGGGAAGCATTCGGAGATAAATGTGAATTCAAGGGCTCCCTGTCTGGCAGTAATGCTGGAATTACAAGCATTGAATTTGATAGTGCTGGAGCTTACCTATTAGCAGCTTCAAATGATTTTGCAAGCCGAATCTGGACTGTGGATGATTATCGATTACGGCACACACTCACAGGCCACAGCGGGAAAGTCCTCTCTGCCAAGTTCCTGCTGGACAATGCACGGATTGTCTCAGGAAGTCACGACCGGACCCTCAAACTCTGGGATCTCCGCAGCAAAGTCTGCATAAAAACAGTGTTTGCAGGATCCAGCTGCAATGACATTGTTTGCACTGAACAATGTGTAATGAGTGGACATTTTGACAAGAAAATTCGTTTCTGGGATATCCGGTCAGAGAGTGTGGTCCGAGAGATGGAACTGTTAGGGAAGATCACTGCTCTGGACCTAAACCCTGAGAGAACTGAGCTCCTGAGCTGCTCCCGTGATGACCTGCTAAAAGTCATCGACCTCCGGACAAATGCAGTCAAACAGACATTCAGTGCGCCTGGATTCAAATGCGGCTCTGACTGGACCCGGGTTGTCTTCAGCCCTGATGGCAGTTACGTGGCAGCAGGCTCAGCCGAGGGTTCTCTTTATGTCTGGAGTGTGCTGACAGGGAAAGTGGAGAAGGTTCTTTCAAAACAGCACAGCTCTTCTATCAATGCGGTGGCGTGGGCCCCCTCGGGCTTACATGTTGTCAGTGTGGACAAAGGAAGCAGAGCTGTGCTGTGGGCACAGCCTTGA-GGTACC |
| pcDNA3.1-Atg16l1-ΔWD40 | GCTAGC-ATGGATTACAAGGATGACGACGATAAGGGTGGAGGTGGATCTTCGTCGGGCCTGCGCGCCGCAGACTTTCCCCGCTGGAAGCGTCACATCGCGGAGGAACTGAGGCGCCGGGACCGACTGCAGAGGCAGGCGTTCGAGGAGATCATTCTGCAGTATACCAAGTTGCTGGAAAAGTCAGATCTTCATTCAGTATTGACCCAGAAACTACAAGCAGAAAAGCATGACATGCCAAATAGGCATGAAATAAGTCCTGGACATGATGGTGCGTGGAATGATAGTCAACTACAAGAAATGGCCCAGTTGAGGATCAAACACCAGGAAGAGCTGACCGAACTGCACAAGAAGCGTGGGGAGTTAGCTCAGTTGGTGATTGACCTGAACAACCAAATGCAGCAGAAGGACAAGGAGATACAGATGAATGAAGCAAAGATTTCGGAGTATTTACAGACCATCTCTGACCTGGAGACAAACTGCCTGGACCTGCGCACCAAACTGCAGGACCTCGAGGTAGCCAACCAGACCCTGAAGGATGAGTATGACGCCCTGCAGATTACTTTTACTGCCCTAGAAGAGAAACTGAGGAAAACTACTGAGGAGAACCAGGAACTGGTCACCAGATGGATGGCTGAGAAGGCCCAAGAAGCCAATCGCCTCAATGCAGAGAATGAGAAGGACTCCAGGAGGCGTCAAGCACGGCTGCAGAAGGAGCTTGCAGAAGCAGCAAAGGAACCTCTACCTGTTGAACAGGATGATGACATTGAAGTCATTGTGGATGAGACCTCAGACCACACAGAAGAGACCTCTCCCGTCCGAGCTGTCAGCAGAGCAGCTACTAAGCGACTCTCGCAGCCTGCTGGAGGCCTTCTGGATTCTATCACTAATATCTTTGGGAGACGCTCTGTCTCTTCCATCCCAGTCCCCCAGGATATCATGGACACTCATCCTGCTTCTGGTAAAGATGTGAGAGTCCCAACTACTGCCTCGTATGTCTTCGATTGA-GGTACC |
| PLVX-Flag-Setdb1 | TCTAGAGCCACCATGGATTACAAGGATGACGACGATAAGGGTGGAGGTGGATCTTCCTCCCTCCCTGGGTGCATGAGTTTGGCTGCAGCGCCAGCTGCAGCTGACTCTGCAGAGATTGCTGAGCTGCAGCAGGCGGTGGTTGAAGAGCTGGGTATCTCTATGGAGGAACTTCGTCAGTACATTGATGAGGAACTGGAAAAGATGGACTGCATACAGCAGCGCAAGAAGCAGCTCGCAGAGCTGGAGACGTGGGTACTACAGAAAGAGTCTGAAGTGGCTTATGTTGATCGGCTGTTTGATGATGCATCCAGGGAAGTGACTAACTGTGAGTCTTTGGTGAAGGATTTCTACTCTAAGCTGGGACTACAGTATCATGACAGTAGCTCTGAGGATGAAGCTTCCCGGCCCACAGAGATCATTGAGATTCCTGATGAAGATGATGATGTCCTCAGTATTGATTCAGGTGATGCTGGGAGCAGAACTCCAAAAGACCAGAAGCTTCGTGAAGCTATGGCTGCCTTAAGAAAATCAGCTCAAGATGTCCAGAAGTTCATGGATGCTGTCAACAAGAAAAGCAGTTCTCAAGATCTACATAAAGGAACCTTGGGTCAGGTGTCTGGAGAACTGAGCAAAGATGGGGACCTGATAGTCAGCATGCGGATTCTGGGCAAGAAGAGGACTAAGACATGGCACAAAGGCACCCTTATTGCCATCCAGACTGTTGGGCTAGGAAAAAAATACAAAGTGAAATTTGACAACAAAGGAAAGAGTCTGCTATCTGGGAACCATATTGCCTATGATTACCACCCTCCCGCTGACAAGCTGTTTGTGGGCAGTCGAGTGGTGGCCAAGTACAAAGATGGAAATCAGGTCTGGCTTTATGCTGGCATTGTAGCTGAGACCCCTAACGTCAAGAACAAGCTCAGATTTTTAATTTTTTTTGATGATGGCTATGCTTCCTATGTCACTCAGTCAGAGCTTTATCCCATTTGCCGACCACTAAAAAAGACTTGGGAGGACATAGAAGATAGCTCCTGCCGAGACTTCATAGAGGAATATATCACTGCCTATCCAAACCGCCCAATGGTACTTCTCAAGAGTGGGCAGCTTATCAAGACTGAGTGGGAAGGCACATGGTGGAAGTCTCGAGTTGAAGAGGTGGATGGCAGCCTAGTCAGGATCCTCTTTCTGGATGACAAAAGATGTGAGTGGATATATCGAGGCTCTACACGCCTGGAACCTATGTTTAGTATGAAGACATCCTCAGCCTCTGCAATGGAGAAGAAGCAAGGGGGGCAACTCAGAACCCGTCCTAATATGGGTGCTGTGAGGAGCAAAGGTCCTGTTGTTCAGTATACACAGGATCTAACTGGTACTGGAATCCAGTTTAAGCCCATGGAGCCCCTACAGCCTATAGCTCCACCGGCCCCACTTCCTATACCTCCTCTTTCCCCCCAAGCAGCTGACACTGACTTAGAAAGCCAACTTGCACAATCACGGAAACAAGTAGCCAAGAAGAGCACATCATTCCGACCAGGATCTGTGGGCTCCGGCCATTCCTCCCCTACTTCATCCACACTCAGTGAAAATGTGTCTGCTGGGAAACTTGGGATAAACCAGACATATCGGTCACCTTTGGCCTCAGTAACATCTACCCCAGCATCTGCAGCCCCTCCAGTCCCTCCAGTCCCACCAGGGCCTCCAACCCCTCCAGGGCCTCCAGCTCCTCCAGGGCCTCTAGCTCCTCCAGCCTTCCATGGCATGTTAGAGCGGGCACCAGCTGAGCCCTCCTACCGAGCCCCCATGGAGAAGCTTTTCTATTTACCTCATGTCTGCAGTTACACTTGTTTGTCCCGGATCAGACCCATGAGAAACGAACAGTATCGGGGCAAGAACCCTCTATTAGTTCCACTTCTGTATGACTTCCGGAGGATGACAGCACGGCGCAGAGTTAACCGCAAAATGGGCTTTCATGTAATCTATAAGACACCCTGTGGTCTCTGCCTTCGGACGATGCAGGAGATAGAGCGCTACCTTTTTGAGACTGGCTGTGACTTTCTGTTCCTGGAGATGTTCTGTTTGGATCCATATGTTCTTGTTGACAGAAAGTTTCAACCCTTTAAGCCTTTTTACTATATTTTGGACATCACCTATGGCAAGGAAGATGTTCCCCTGTCCTGTGTTAATGAGATTGACACAACTCCCCCACCCCAGGTGGCCTACAGCAAGGAACGCATTCCTGGCAAGGGTGTTTTCATTAACACAGGCCCTGAATTTCTGGTTGGCTGTGACTGCAAGGATGGGTGTCGGGATAAATCCAAATGTGCCTGCCACCAGCTAACTATCCAGGCCACAGCCTGTACCCCAGGGGGCCAAGTCAACCCTAACTCTGGCTACCAGTATAAAAGACTAGAAGAGTGTCTGCCCACAGGGGTTTATGAGTGTAACAAACGCTGCAATTGTGACCCAAACATGTGCACAAATCGGTTGGTGCAGCATGGTCTGCAGGTTCGACTACAGCTGTTTAAGACACAGAACAAGGGCTGGGGTATCCGCTGCTTGGATGATATTGCCAAAGGCTCTTTTGTCTGCATTTATGCAGGCAAAATCCTGACAGATGACTTTGCAGACAAAGAAGGCCTGGAGATGGGTGATGAGTACTTTGCAAATCTGGACCACATTGAAAGTGTGGAGAACTTCAAGGAAGGATATGAGAGTGATGTCCCCACTTCCTCTGACAGCAGTGGGGTAGATATGAAGGACCAGGAAGATGGCAACAGCGGTTCAGAGGACCCTGAAGAATCCAATGATGACAGCTCTGATGATAACTTCTGTAAGGATGAGGACTTCAGCACCAGTTCAGTGTGGCGTAGCTATGCTACCCGGAGGCAGACTCGGGGTCAAAAGGAGAATGAATTGTCTGAGATGACTTCCAAGGACTCCCGCCCCCCAGACCTCGGGCCTCCACATGTTCCTATCCCTTCCTCAGTATCTGTAGGGGGCTGCAATCCACCTTCCTCTGAAGAGACACCCAAGAACAAGGTGGCCTCGTGGTTGAGTTGCAATAGTGTCAGTGAAGGTGGATTTGCTGACTCTGACAGCCGTTCTTCCTTCAAGACTAGTGAAGGTGGAGATGGCCGTGCTGGGGGAGGCCGGGGAGAGGCTGAAAGGGCCTCTACCTCAGGATTGAGCTTCAAGGATGAAGGAGACAATAAGCAGCCTAAAAAAGAGGACCCTGAGAACCGAAACAAGATGCCAGTAGTTACTGAAGGCTCTCAGAATCATGGACATAATCCTCCCATGAAGTCTGAAGGGCTTCGCCGATCAGCTAGTAAAATGTCTGTGCTCCAGAGCCAGCGAGTTGTGACTTCTACTCAGTCAAACCCTGATGACATCCTGACACTGTCCAGCAGCACAGAGAGTGAGGGGGAAAGTGGAACCAGCCGAAAGCCCACTGCTGGTCACACTTCAGCCACAGCTGTTGATAGTGATGACATCCAGACCATCTCTTCTGGCTCTGACGGTGATGACTTTGAGGACAAGAAGAACTTGTCAGGACCAACAAAGCGCCAGGTGGCAGTAAAATCAACCCGAGGCTTTGCTCTTAAATCAACCCATGGTATTGCCATTAAATCAACCAACATGGCTTCCGTGGACAAGGGGGAGAGTGCACCAGTTCGTAAGAACACACGCCAGTTCTATGATGGTGAAGAGTCTTGCTACATCATTGATGCCAAACTTGAAGGCAACCTAGGCCGCTACCTCAATCACAGTTGCAGCCCCAACCTGTTTGTCCAGAATGTGTTTGTGGATACCCATGATCTTCGCTTCCCTTGGGTGGCCTTCTTTGCCAGCAAGAGAATCCGGGCTGGAACAGAACTCACTTGGGACTACAACTACGAAGTGGGCAGTGTGGAAGGCAAGGAGCTGCTGTGCTGCTGTGGGGCCATTGAATGCAGAGGGAGACTTCTTTAAGCGGCCGC |
| PLVX-Flag-Setdb1-ΔSET | TCTAGAGCCACCATGGATTACAAGGATGACGACGATAAGGGTGGAGGTGGATCTTCCTCCCTCCCTGGGTGCATGAGTTTGGCTGCAGCGCCAGCTGCAGCTGACTCTGCAGAGATTGCTGAGCTGCAGCAGGCGGTGGTTGAAGAGCTGGGTATCTCTATGGAGGAACTTCGTCAGTACATTGATGAGGAACTGGAAAAGATGGACTGCATACAGCAGCGCAAGAAGCAGCTCGCAGAGCTGGAGACGTGGGTACTACAGAAAGAGTCTGAAGTGGCTTATGTTGATCGGCTGTTTGATGATGCATCCAGGGAAGTGACTAACTGTGAGTCTTTGGTGAAGGATTTCTACTCTAAGCTGGGACTACAGTATCATGACAGTAGCTCTGAGGATGAAGCTTCCCGGCCCACAGAGATCATTGAGATTCCTGATGAAGATGATGATGTCCTCAGTATTGATTCAGGTGATGCTGGGAGCAGAACTCCAAAAGACCAGAAGCTTCGTGAAGCTATGGCTGCCTTAAGAAAATCAGCTCAAGATGTCCAGAAGTTCATGGATGCTGTCAACAAGAAAAGCAGTTCTCAAGATCTACATAAAGGAACCTTGGGTCAGGTGTCTGGAGAACTGAGCAAAGATGGGGACCTGATAGTCAGCATGCGGATTCTGGGCAAGAAGAGGACTAAGACATGGCACAAAGGCACCCTTATTGCCATCCAGACTGTTGGGCTAGGAAAAAAATACAAAGTGAAATTTGACAACAAAGGAAAGAGTCTGCTATCTGGGAACCATATTGCCTATGATTACCACCCTCCCGCTGACAAGCTGTTTGTGGGCAGTCGAGTGGTGGCCAAGTACAAAGATGGAAATCAGGTCTGGCTTTATGCTGGCATTGTAGCTGAGACCCCTAACGTCAAGAACAAGCTCAGATTTTTAATTTTTTTTGATGATGGCTATGCTTCCTATGTCACTCAGTCAGAGCTTTATCCCATTTGCCGACCACTAAAAAAGACTTGGGAGGACATAGAAGATAGCTCCTGCCGAGACTTCATAGAGGAATATATCACTGCCTATCCAAACCGCCCAATGGTACTTCTCAAGAGTGGGCAGCTTATCAAGACTGAGTGGGAAGGCACATGGTGGAAGTCTCGAGTTGAAGAGGTGGATGGCAGCCTAGTCAGGATCCTCTTTCTGGATGACAAAAGATGTGAGTGGATATATCGAGGCTCTACACGCCTGGAACCTATGTTTAGTATGAAGACATCCTCAGCCTCTGCAATGGAGAAGAAGCAAGGGGGGCAACTCAGAACCCGTCCTAATATGGGTGCTGTGAGGAGCAAAGGTCCTGTTGTTCAGTATACACAGGATCTAACTGGTACTGGAATCCAGTTTAAGCCCATGGAGCCCCTACAGCCTATAGCTCCACCGGCCCCACTTCCTATACCTCCTCTTTCCCCCCAAGCAGCTGACACTGACTTAGAAAGCCAACTTGCACAATCACGGAAACAAGTAGCCAAGAAGAGCACATCATTCCGACCAGGATCTGTGGGCTCCGGCCATTCCTCCCCTACTTCATCCACACTCAGTGAAAATGTGTCTGCTGGGAAACTTGGGATAAACCAGACATATCGGTCACCTTTGGCCTCAGTAACATCTACCCCAGCATCTGCAGCCCCTCCAGTCCCTCCAGTCCCACCAGGGCCTCCAACCCCTCCAGGGCCTCCAGCTCCTCCAGGGCCTCTAGCTCCTCCAGCCTTCCATGGCATGTTAGAGCGGGCACCAGCTGAGCCCTCCTACCGAGCCCCCATGGAGAAGCTTTTCTATTTACCTCATGTCTGCAGTTACACTTGTTTGTCCCGGATCAGACCCATGAGAAACGAACAGTATCGGGGCAAGAACCCTCTATTAGTTCCACTTCTGTATGACTTCCGGAGGATGACAGCACGGCGCAGAGTTAACCGCAAAATGGGCTTTCATGTAATCTATAAGACACCCTGTGGTCTCTGCCTTCGGACGATGCAGGAGATAGAGCGCTACCTTTTTGAGACTGGCTGTGACTTTCTGTTCCTGGAGATGTTCTGTTTGGATCCATATGTTCTTGTTGACAGAAAGTTTCAACCCTTTAAGCCTTTTTACTATATTTTGGACATCACCTATGGCAAGGAAGATGTTCCCCTGTCCTGTGTTAATGAGATTGACACAACTCCCCCACCCCAGGTGGCCTACAGCAAGGAACGCATTCCTGGCAAGGGTGTTTTCATTAACACAGGCCCTGAATTTCTGTAAGCGGCCGC |
| PLVX-Flag-Setdb1-ΔTudor | TCTAGAGCCACCATGGATTACAAGGATGACGACGATAAGGGTGGAGGTGGATCTTCCTCCCTCCCTGGGTGCATGAGTTTGGCTGCAGCGCCAGCTGCAGCTGACTCTGCAGAGATTGCTGAGCTGCAGCAGGCGGTGGTTGAAGAGCTGGGTATCTCTATGGAGGAACTTCGTCAGTACATTGATGAGGAACTGGAAAAGATGGACTGCATACAGCAGCGCAAGAAGCAGCTCGCAGAGCTGGAGACGTGGGTACTACAGAAAGAGTCTGAAGTGGCTTATGTTGATCGGCTGTTTGATGATGCATCCAGGGAAGTGACTAACTGTGAGTCTTTGGTGAAGGATTTCTACTCTAAGCTGGGACTACAGTATCATGACAGTAGCTCTGAGGATGAAGCTTCCCGGCCCACAGAGATCATTGAGATTCCTGATGAAGATGATGATGTCCTCAGTATTGATTCAGGTGATGCTGGGAGCAGAACTCCAAAAGACCAGAAGCTTCGTGAAGCTATGGCTGCCTTAAGAAAATCAGCTCAAGATGTCCAGAAGTTCATGGATGCTGTCAACAAGAAAAGCAGTTCTCAAGATCTACATAAAGGAACCTTGGGTCAGGTGTCTGGAGAACTGAGCAAAGATGGGGACCTGATAGTCAGCATGCGGATTCTGGGCAAGAAGAGGACTAAGACATGGCACAAAGGCACCCTTATTGCCATCCAGACTGTTGGGCTAGGAAAAAAATACAAAGTGAAATTTGACAACAAAGGAAAGAGTCTGCTATCTGGGAACCATATTGCCTATGATTACCACCCTCCCGCTGACTCCTCAGCCTCTGCAATGGAGAAGAAGCAAGGGGGGCAACTCAGAACCCGTCCTAATATGGGTGCTGTGAGGAGCAAAGGTCCTGTTGTTCAGTATACACAGGATCTAACTGGTACTGGAATCCAGTTTAAGCCCATGGAGCCCCTACAGCCTATAGCTCCACCGGCCCCACTTCCTATACCTCCTCTTTCCCCCCAAGCAGCTGACACTGACTTAGAAAGCCAACTTGCACAATCACGGAAACAAGTAGCCAAGAAGAGCACATCATTCCGACCAGGATCTGTGGGCTCCGGCCATTCCTCCCCTACTTCATCCACACTCAGTGAAAATGTGTCTGCTGGGAAACTTGGGATAAACCAGACATATCGGTCACCTTTGGCCTCAGTAACATCTACCCCAGCATCTGCAGCCCCTCCAGTCCCTCCAGTCCCACCAGGGCCTCCAACCCCTCCAGGGCCTCCAGCTCCTCCAGGGCCTCTAGCTCCTCCAGCCTTCCATGGCATGTTAGAGCGGGCACCAGCTGAGCCCTCCTACCGAGCCCCCATGGAGAAGCTTTTCTATTTACCTCATGTCTGCAGTTACACTTGTTTGTCCCGGATCAGACCCATGAGAAACGAACAGTATCGGGGCAAGAACCCTCTATTAGTTCCACTTCTGTATGACTTCCGGAGGATGACAGCACGGCGCAGAGTTAACCGCAAAATGGGCTTTCATGTAATCTATAAGACACCCTGTGGTCTCTGCCTTCGGACGATGCAGGAGATAGAGCGCTACCTTTTTGAGACTGGCTGTGACTTTCTGTTCCTGGAGATGTTCTGTTTGGATCCATATGTTCTTGTTGACAGAAAGTTTCAACCCTTTAAGCCTTTTTACTATATTTTGGACATCACCTATGGCAAGGAAGATGTTCCCCTGTCCTGTGTTAATGAGATTGACACAACTCCCCCACCCCAGGTGGCCTACAGCAAGGAACGCATTCCTGGCAAGGGTGTTTTCATTAACACAGGCCCTGAATTTCTGGTTGGCTGTGACTGCAAGGATGGGTGTCGGGATAAATCCAAATGTGCCTGCCACCAGCTAACTATCCAGGCCACAGCCTGTACCCCAGGGGGCCAAGTCAACCCTAACTCTGGCTACCAGTATAAAAGACTAGAAGAGTGTCTGCCCACAGGGGTTTATGAGTGTAACAAACGCTGCAATTGTGACCCAAACATGTGCACAAATCGGTTGGTGCAGCATGGTCTGCAGGTTCGACTACAGCTGTTTAAGACACAGAACAAGGGCTGGGGTATCCGCTGCTTGGATGATATTGCCAAAGGCTCTTTTGTCTGCATTTATGCAGGCAAAATCCTGACAGATGACTTTGCAGACAAAGAAGGCCTGGAGATGGGTGATGAGTACTTTGCAAATCTGGACCACATTGAAAGTGTGGAGAACTTCAAGGAAGGATATGAGAGTGATGTCCCCACTTCCTCTGACAGCAGTGGGGTAGATATGAAGGACCAGGAAGATGGCAACAGCGGTTCAGAGGACCCTGAAGAATCCAATGATGACAGCTCTGATGATAACTTCTGTAAGGATGAGGACTTCAGCACCAGTTCAGTGTGGCGTAGCTATGCTACCCGGAGGCAGACTCGGGGTCAAAAGGAGAATGAATTGTCTGAGATGACTTCCAAGGACTCCCGCCCCCCAGACCTCGGGCCTCCACATGTTCCTATCCCTTCCTCAGTATCTGTAGGGGGCTGCAATCCACCTTCCTCTGAAGAGACACCCAAGAACAAGGTGGCCTCGTGGTTGAGTTGCAATAGTGTCAGTGAAGGTGGATTTGCTGACTCTGACAGCCGTTCTTCCTTCAAGACTAGTGAAGGTGGAGATGGCCGTGCTGGGGGAGGCCGGGGAGAGGCTGAAAGGGCCTCTACCTCAGGATTGAGCTTCAAGGATGAAGGAGACAATAAGCAGCCTAAAAAAGAGGACCCTGAGAACCGAAACAAGATGCCAGTAGTTACTGAAGGCTCTCAGAATCATGGACATAATCCTCCCATGAAGTCTGAAGGGCTTCGCCGATCAGCTAGTAAAATGTCTGTGCTCCAGAGCCAGCGAGTTGTGACTTCTACTCAGTCAAACCCTGATGACATCCTGACACTGTCCAGCAGCACAGAGAGTGAGGGGGAAAGTGGAACCAGCCGAAAGCCCACTGCTGGTCACACTTCAGCCACAGCTGTTGATAGTGATGACATCCAGACCATCTCTTCTGGCTCTGACGGTGATGACTTTGAGGACAAGAAGAACTTGTCAGGACCAACAAAGCGCCAGGTGGCAGTAAAATCAACCCGAGGCTTTGCTCTTAAATCAACCCATGGTATTGCCATTAAATCAACCAACATGGCTTCCGTGGACAAGGGGGAGAGTGCACCAGTTCGTAAGAACACACGCCAGTTCTATGATGGTGAAGAGTCTTGCTACATCATTGATGCCAAACTTGAAGGCAACCTAGGCCGCTACCTCAATCACAGTTGCAGCCCCAACCTGTTTGTCCAGAATGTGTTTGTGGATACCCATGATCTTCGCTTCCCTTGGGTGGCCTTCTTTGCCAGCAAGAGAATCCGGGCTGGAACAGAACTCACTTGGGACTACAACTACGAAGTGGGCAGTGTGGAAGGCAAGGAGCTGCTGTGCTGCTGTGGGGCCATTGAATGCAGAGGGAGACTTCTTTAAGCGGCCGC |

**Data S1.** mouse *Map1lc3b* promoter sequence information

m-*Map1lc3b* promoter-wt：

CCCACCCACCCACCCAGACAGGGTTTCTTTGTAAAGCCCTGGCTGTCTTGGAACTCAGTTTGTAGACCAGGCTGGCCTCGAACTCAGAAATCTGCCTGCCTCTGCCTCCCGAGTGCTGGGATTAAAGTCGTGCACCACCACGCTCAGCTTTTTTAAAGGTTTTTACTTCAAGACTTCATGTGTATGAGTGTTTGCCTGTATGTTTACATATGTACCATGTGTGTGCCCTTGCCTGAGGAGACCAGAAGAGGATGTTAGATCCCCCAGAGTGGGAGTTACAGCTGAGCTGCCATGTGGGTCTGAGAACCAAATCCAGGTCCTCAACTAGAGCAGCCAGTGGTGGTTGGTTTTTTTGTTTTGTTTTTTTTTTTTTTTTTTTGGTTTTTCAAGACAGGGTTTCTCTGGCAGCCAGTGTTCTTAACCACCGGACCATCTCACCAGTCCCATGCGTGCCACCCTTCTTCTCCTTGTGAGACAGGTTCTCACAATGTAGGAATTTGTTGTGTAGAACAGGCTATTTCAGACTCACAGAAACCAGACTGCATCTCCCTTCTGGACACTGAGATAAAGGCCTAGGCACCGAGTCCCACTTGGCCTTGAACTTCTGATAAGCCTGCTTCAGTCTCCAGTGTGCTAGGATGACATGCCTTGGGACACCAGATCTGGCTTCCATTTTGACTTTTAGCCTGGTCTCTTGCACCTCAGACCAGAAGCTTCATACAAAACAACAAAGTTCGGGCAATTAACAATGCAAAGCAAGCAAACAAAGGAAAGTAACCAGCCCTCAGGATTTCACTCCATAATGTGTTTTCAGCTTTTCCCGGTCACAGAGATGTAGAAGATCCCTTGGACAAACAGCACTTGAAGAAGGTTATTTCCATTTAAAGGAGGAAGTCTGGGCTGTAGGGATAGTACAGTGTCAGGTGGTAGGTCTGCAGGGTCACAAGGCTGGCTCAGATGTGGCATCACTGGGGGTGGGATGGGGGTGAGGTGGCTGCTCCACACTCTAGACCTGTCTGTCACCTGCTCCTACAAACTGAAGATAGGAAGTAGCTTGAGGCCCAGAGAAAGTGCTAAACCGTGGCTGACTGACCATTGGCCTGGCCTGAGGAGACTGAAACCTGTCCTAGCCTGTAATCAGGCCCAGGCTCAGCAGGAGTCAAAGTGAGGACATGCCTGTGATAAAGCCACCCTGGGGTCACCTGAGGTAGAAGGATCTGTCTGAGAAGGGACAGCTGAGATAAAGCTCTTGGGACAGAACTGTGCTCTGTGGCTTTCCAAGGTGGATCCTGTCGGGATCAGGCCTCTGCTGCAGTTCCTGGACACAGTCTGATTGCTCACCAACCAAAAAAATCTAAAAAAACAAAAAACGTGATAGGTGGTAGTAAGGAAATGAGGGCACACATGGGCTAGCACCCTGGAAAGATAGGTGAGCCATGCCCCTCAAATTCCATGCTCAGCAAGCTCAGACACATAAAAGGGCTTTTATGGGAAGAGAGAGTGGACAGGTTGCACAGCAGGTGTTGTCATCGTCTTTCTTTTTGCTGGAACAAGTCATTGATCTTGCCCTCCAGTGACAAGTCTGTAGTCCCTAAGACCTTGCTTCTCTGCGACTTCGACTTAGGAGTAAATACTTGTGTTTATGGCAGCACAAGACTCGCAGGGGAAGAGCCACAAGATCAGATCCAACCATAAGTCTCCAGACGTCTCCATAATCGTAAAGATTTAGCAGGCAAGACCACAAGTCAGTTTTTTGTTATTTGGGTTGGGGGTGGGTAGAATTTAGGAATATCAAATACAGCGGCAAGGGAGCGGACATTGGACCACCAGCTGGATCATGCAAGGACTGAAGGGGAATGAAGTCTGCAGGACTCCATCGCACCAGCGCCCGACCCACGCAGACAGTTAACAGATGCTCGCCCAGCGTGTGCCCAGCGAGCGCCCAGCGTAGCTGGCGTAGGCGCAGGCGCATGGCTTGGGCGCGTGCACGCTTGCGCGCCGCCGAGCTTCCGCCACGCCTGTCATGGCCGCTCTCGGGGGCCGCCCTCGGCCCCACCCTCGGTGACGCGCCGCGAGTCACCTGACTCGGCTGCGGACTGAGACACACACAAGGGAAGTGATCGTCGCCGGAGTCAGATCGTCTGGCTCGGGACCCCCGCGCGCCATGCCGTCCGAGAAGACCTTCAAGCAGCGCCGGAGC

m-*Map1lc3b* promoter-mut：

CCCACCCACCCACCCAGACAGGGTTTCTTTGTAAAGCCCTGGCTGTCTTGGAACTCAGTTTGTAGACCAGGCTGGCCTCGAACTCAGAAATCTGCCTGCCTCTGCCTCCCGAGTGCTGGGATTAAAGTCGTGCACCACCACGCTCAGCTTTTTTAAAGGTTTTTACTTCAAGACTTCATGTGTATGAGTGTTTGCCTGTATGTTTACATATGTACCATGTGTGTGCCCTTGCCTGAGGAGACCAtAtGtGcAaGaTtGATCCCCCAGAGTGGGAGTTACAGCTGAGCTGCCATGTGGGTCTGAGAACCAAATCCAGGTCCTCAACTAGAGCAGCCAGTGGTGGTTGGTTTTTTTGTTTTGTTTTTTTTTTTTTTTTTTTGGTTTTTCAAGACAGGGTTTCTCTGGCAGCCAGTGTTCTTAACCACCGGACCATCTCACCAGTCCCATGCGTGCCACCCTTCTTCTCCTTGTGAGACAGGTTCTCACAATGTAGGAATTTGTTGTGTAGAACAGGCTATTTCAGACTCACAGAAACCAGACTGCATCTCCCTTCTGGACACTGAGATAAAGGCCTAGGCACCGAGTCCCACTTGGCCTTGAACTTCTGATAAGCCTGCTTCAGTCTCCAGTGTGCTAGGATGACATGCCTTGGGACACCAGATCTGGCTTCCATTTTGACTTTTAGCCTGGTCTCTTGCACCTCAGACCAGAAGCTTCATACAAAACAACAAAGTTCGGGCAATTAACAATGCAAAGCAAGCAAACtAtGcAtAcTtACCAGCCCTCAGGATTTCACTCCATAATGTGTTTTCAGCTTTTCCCGGTCACAGAGATGTAGAAGATCCCTTGGACAAACAGCACTTGAAGAAGGTTATTTCCATTTAcAcGtGcAtGaCaGGGCTGTAGGGATAGTACAGTGTCAGGTGGTAGGTCTGCAGGGTCACAAGGCTGGCTCAGATGTGGCATCACTGGGGGTGGGATGGGGGTGAGGTGGCTGCTCCACACTCTAGACCTGTCTGTCACCTGCTCCTACAAACTGAtGtTcGcAtGaAtCTTGAGGCCCAGAGAAAGTGCTAAACCGTGGCTGACTGACCATTGGCCTGGCCTGAGGAGACTGAAACCTGTCCTAGCCTGTAATCAGGCCCAGGCTCAGCAGGAGTCAAAGTGAGGACATGCCTGTGATAAAGCCACCCTGGGGTCACCTGAGGTAGAAGGATCTGTCTGAGAAGGGACAGCTGAGATAAAGCTCTTGGGACAGAACTGTGCTCTGTGGCTTTCCAAGGTGGATCCTGTCGGGATCAGGCCTCTGCTGCAGTTCCTGGACACAGTCTGATTGCTCACCAACCAAAAAAATCTAAAAAAACAAAAAACGTGATAGGTGGTtGaAtGcAtAgGtGGGCACACATGGGCTAGCACCCTGGAAAGATAGGTGAGCCATGCCCCTCAAATTCCATGCTCAGCAAGCTCAGACACATAAAAGGGCTTTTATGGGAAGAGAGAGTGGACAGGTTGCACAGCAGGTGTTGTCATCGTCTTTCTTTTTGCTGGAACAAGTCATTGATCTTGCCCTCCAGTGACAAGTCTGTAGTCCCTAAGACCTTGCTTCTCTGCGACTTCGACTTAGGAGTAAATACTTGTGTTTATGGCAGCACAAGACTCGCAGGGGAAGAGCCACAAGATCAGATCCAACCATAAGTCTCCAGACGTCTCCATAATCGTAAAGATTTAGCAGGCAAGACCACAAGTCAGTTTTTTGTTATTTGGGTTGGGGGTGGGTAGAATTTAGGAATATCAAATACAGCGGCAAGGGAGCGGACATTGGACCACCAGCTGGATCATGCAAGGACTGAAGGGGAATGAAGTCTGCAGGACTCCATCGCACCAGCGCCCGACCCACGCAGACAGTTAACAGATGCTCGCCCAGCGTGTGCCCAGCGAGCGCCCAGCGTAGCTGGCGTAGGCGCAGGCGCATGGCTTGGGCGCGTGCACGCTTGCGCGCCGCCGAGCTTCCGCCACGCCTGTCATGGCCGCTCTCGGGGGCCGCCCTCGGCCCCACCCTCGGTGACGCGCCGCGAGTCACCTGACTCGGCTGCGGACTGAGACACACACAAGGGAAGTGATCGTCGCCGGAGTCAGATCGTCTGGCTCGGGACCCCCGCGCGCCATGCCGTCCGAGAAGACCTTCAAGCAGCGCCGGAGC
